# Safety and tolerability of intravenous liposomal GM1 in patients with Parkinson disease: A single-center open-label clinical phase I trial (NEON trial)

**DOI:** 10.1371/journal.pmed.1004472

**Published:** 2025-05-13

**Authors:** Stefan Halbherr, Stefanie Lerch, Sebastian Bellwald, Petra Polakova, Bettina Bannert, Marie Roumet, Roch-Philippe Charles, Martin A. Walter, Corrado Bernasconi, Valérie Lisa Halbherr, Camille Peitsch, Pascal C. Baumgartner, Céline Kaufmann, Vanessa Aires, Heinrich P. Mattle, Alain Kaelin-Lang, Andreas Hartmann, Michael Schuepbach

**Affiliations:** 1 InnoMedica Schweiz AG, Bern, Switzerland; 2 Skin and Soft Tissue Research Center, University Children’s Hospital Zurich, Zurich, Switzerland; 3 Institute of Neurology, Konolfingen, Switzerland; 4 Swiss Pediatric Oncology Group, Bern, Switzerland; 5 Department of Clinical Research, University of Basel, Basel, Switzerland; 6 Department of Clinical Research, University of Bern, Bern, Switzerland; 7 Swiss Group for Clinical Cancer Research, Bern, Switzerland; 8 St. Anna Hospital, University of Lucerne, Lucerne, Switzerland; 9 Limites Medical Research Ltd., Vacallo, Switzerland; 10 University of Bern, Bern, Switzerland; 11 Faculty of Biomedical Sciences, Università della Svizzera Italiana, Lugano, Switzerland; 12 Neurocenter of Southern Switzerland, Ente Ospedaliero Cantonale, Lugano, Switzerland; 13 Département de Neurologie, Assistance-Publique Hôpitaux de Paris, Groupe Hospitalier Pitié-Salpêtrière, Paris, France; University of Cambridge, UNITED KINGDOM OF GREAT BRITAIN AND NORTHERN IRELAND

## Abstract

**Background:**

Parkinson disease (PD) is a chronic progressive neurodegenerative disorder leading to motor and non-motor impairment, often resulting in severe loss of quality of life. There are symptomatic treatments without effect on the progression of PD. A disease-modifying treatment that could ideally stop the neurodegenerative process is direly needed. Monosialotetrahexosylganglioside (GM1) is a promising molecule with neuroprotective effects in preclinical models of PD and has yielded encouraging results in patients with PD in a randomized placebo-controlled trial. Talineuren (TLN) is a liposomal formulation of GM1 that has been shown to cross the blood–brain barrier in animals. We assessed the safety and pharmacokinetics (PK) of TLN in patients with PD.

**Methods and findings:**

We prospectively enrolled 12 patients with PD into a single-center, open-label phase I trial to assess the safety and tolerability of weekly infusions with TLN. The maximum suitable dose of TLN was determined by dose escalation in three patients. All three patients tolerated the predetermined maximal dose of 720 mg. Subsequently, these and nine additional patients received weekly infusions at the maximum suitable dose of 720 mg TLN over two months (1 patient stopped prematurely). PK were determined for the additional nine patients as a secondary outcome measure. *C*_max_ was reached 4 h after infusion start for all but one participant, who reached *C*_max_ after 1 h, while the median plasma half-life was reached at 12.6 h.

All adverse events were continuously assessed as the primary objective and coded according to the Medical Dictionary for Regulatory Activities (MedDRA). Clinical manifestations of PD were assessed as secondary outcomes using the Movement Disorders Society Unified Parkinson’s Disease Rating Scale (MDS-UPDRS), including a levodopa challenge test at baseline and end.

In addition to weekly history taking, scales to measure mood, behavior, quality of life, sleepiness, non-motor symptoms of PD, and cognition were used as further secondary outcomes as well as assessing the Levodopa-Equivalent Daily Dose (LEDD). Overall, 304 adverse events (mean: 25.33; 6–75 events per patient) occurred, 267 of which were mild (mean: 22.25; 3–72 events per patient). 23 were considered related to the study treatment (0–8 events per patient). Very mild-to-severe acute infusion reactions at the second, third, or fourth administration of TLN within the first minutes of the infusion occurred in seven patients. All reported back or neck pain. Other acute infusion reactions were urticaria, plethora, nausea, and chest pain. These adverse reactions disappeared within minutes of stopping the infusion and did not recur when TLN administration was resumed at a very low rate. Beyond the fourth administration, infusions could be given at increased rates up to 370 ml/h, and no acute reaction occurred anymore. The mechanism of this acute infusion reaction remains unclear. Some patients reported mild dizziness for a few hours after TLN following many but not all administrations throughout the study.

Non-motor symptoms of PD, motor parkinsonian signs off medication, and quality of life improved significantly during the treatment phase, including the MDS-UPDRS total score (mean decrease −11.09; 95% Confidence Interval [CI]; −18, −4.1; *p* = 0.006), the Parkinson’s disease Questionnaire-39 (PDQ-39) summary index (mean decrease −2.91; 95% CI; −4.4, −1.4; *p* = 0.005), and the Non-Motor Symptoms Questionnaire (NMS-Quest) (mean decrease −4.27; 95% CI; −6.5, −2.1; *p* = 0.009). No statistically significant improvements were seen in the Montreal Cognitive Assessment (MoCA) (mean decrease −0.73; 95% CI; −2.1, 0.62; *p* = 0.255), Epworth Sleepiness Scale (mean increase 0.09; 95% CI; −2.6, 2.8; *p* > 0.999), Beck Depression Inventory (BDI) (mean decrease −1.27; 95% CI; −3.8, 1.3; *p* = 0.257), and the Starkstein Apathy Scale (mean increase 0.36; 95% CI; −1.6, 2.4; *p* = 0.822). Dopaminergic medications remained stable during the study (LEDD mean increase 8.18; 95% CI; −7.7, 24; *p* = 0.423). While clinical improvements indicate a benefit associated with TLN treatment, the trial design does not allow for definite conclusions regarding efficacy. A randomized, placebo-controlled trial will be required to corroborate our exploratory findings.

**Conclusion:**

TLN is safe and well-tolerated in general. This prospective phase I trial revealed non-allergic habituating acute infusion reactions at the second, third, or fourth treatment that can be prevented by a slower rate of infusion. Importantly, the exploratory results suggest a consistent improvement of signs and symptoms of PD.

**Trial registration:**

The NEON trial is registered at the US National Institutes of Health (ClinicalTrials.gov) #NCT04976127 and in the Swiss National Clinical Trials Portal (SNCTP000004631)

## Author summary

**Why was this study done?:**

- Monosialotetrahexosylganglioside (GM1) is an important component of the neuronal cell membrane. Preclinical research highlights the neurotrophic and immunomodulating potential of GM1.
- A randomized placebo-controlled trial with twice-daily subcutaneous application of GM1 in Parkinson disease (PD) patients has yielded encouraging results.
- Talineuren (TLN) is a liposomal formulation of GM1 and has been designed to improve GM1 biodistribution and to reduce treatment frequency.

**What did the researchers do and find?:**

- An open-label, single-arm interventional Phase I trial is conducted to evaluate the safety, tolerability, and preliminary efficacy of weekly TLN infusions in PD patients.
- No serious adverse event was reported in this study. Some patients experienced mostly mild-to-moderate acute infusion reactions.
- Plasma half-life was reached at 12.6 h.
- After 8 weeks of treatment with 720 mg TLN, assessments for non-motor symptoms of PD, motor symptoms of PD off medication, and quality of life showed a statistically significant improvement compared to the baseline scores. Assessments concerning cognition, sleep quality, depression, and apathy showed no change.

**What do these findings mean?:**

- Weekly intravenous treatment with TLN is generally well tolerated.
- Exploratory efficacy results suggest a benefit of TLN for motor and non-motor symptoms as well as the quality of life of PD patients.
- Randomized, placebo-controlled trials are needed to further evaluate the safety and efficacy of TLN.

## Abstract 3

In this phase 1b study reported by Stefan Halbherr and colleagues, a liposomal formulation of GM1 was assessed in 12 patients with Parkinson disease.

## Introduction

Parkinson disease (PD) is a chronic progressive neurodegenerative disorder with motor signs and non-motor symptoms. Its clinical hallmark is Parkinsonism, i.e., bradykinesia associated with rest tremor or rigidity [1]. Although the motor signs remain the primary defining feature of PD, vegetative, behavioral, and cognitive symptoms may have a predominant effect on quality of life, and often precede motor signs [2]. Histologically, PD is characterized by alpha-synuclein containing neuronal inclusions called Lewy bodies that propagate through the brain resulting in impaired function of numerous systems [3,4]. Some manifestations of PD can symptomatically be relieved with medication or stereotactic procedures, but despite considerable ongoing research efforts [5] there is currently no known disease-modifying treatment available for PD. The most important therapeutic potential currently consists of medications for the substitution of dopamine loss due to degeneration of dopaminergic neurons in the substantia nigra. However, over decades of disease progression levodopa-resistant symptoms become the main cause for disability. A treatment to slow down or even halt the pathological process in PD is direly needed.

The glycosphingolipid GM1 (monosialotetrahexosylganglioside) is an important component of the cell membrane of neurons that is diminished in patients with PD [6]. GM1 has shown neurotrophic and neuroprotective properties in preclinical research [7], and decreased levels of GM1 in patients with PD may contribute to the pathogenesis [8]. Studies in animal models of PD have shown recovery with GM1 treatment [9], and a randomized double-blinded placebo-controlled clinical study in 77 patients treated with twice daily subcutaneous administration of 100 mg GM1 resulted in a significant benefit compared to placebo over 120 weeks [10]. Administration and therapeutic effects of GM1 may be improved by using a neurotropic nanoparticle carrier. Talineuren (TLN) is a liposomal formulation consisting of the Active Pharmaceutical Ingredient (API) GM1 at 6 mg/ml and the carrier liposomes consisting of sphingomyelin and cholesterol. We conducted a phase I safety trial in 12 PD patients receiving weekly TLN infusions over 8 weeks minimum to assess the safety and PK of TLN. Additionally, we analyzed the exploratory efficacy of TLN by comparing clinical assessments at baseline and after 8 weeks of TLN treatment.

## Methods

### Study design

In this single-center, open-label phase I interventional trial, we enrolled 12 patients with PD to assess the safety, tolerability, and preliminary efficacy of liposomal GM1 as add-on medication with weekly intravenous infusions. Three patients received weekly ascending doses for 14 weeks to establish the highest well-tolerated dose (Dose Escalation [DE] group). According to the trial protocol, the highest weekly dose of the API was limited to 720 mg, based on previous use of GM1 in humans [10], and was reached in all three patients. DE patients and 9 additional patients (Dose Consolidation [DC] group) received thereafter weekly TLN with an API dose of 720 mg for 8 weeks between December 13, 2021 and June 20, 2022. The sample size of *n* = 12 patients was based on a previous GM1 safety study [11].

One of these patients stopped participation prematurely. One month after the last administration of TLN, a final safety follow-up visit was performed. In previous use of GM1 in PD patients, no relevant interaction with the dopaminergic medication was reported. Therefore, antiparkinsonian medication was kept stable, but could be adapted at any time, if needed. In two patients with bilateral subthalamic nucleus stimulation, stimulation remained on and parameters unchanged throughout the study.

An independent data safety monitoring board evaluated safety data when the 3 DE patients had reached a GM1 dose of 180 mg and after completion of the DE. The trial was approved by the local ethics committee (Kantonale Ethikkommission Bern) and the competent authority October 30, 2021. The trial is registered at the US National Institute for Health (ClinicalTrials.gov NCT04976127). The trial was conducted according to the principles of the Declaration of Helsinki and Good Clinical Practice guidelines. TLN is provided by the manufacturer and sponsor of the study, InnoMedica Schweiz AG. TLN is produced under GMP conditions at InnoMedica’s Nanofactory in Marly (Fribourg, Switzerland).

### Patients

GM1 use in humans is well studied, and trials using GM1 in PD patients have been performed in the past [10,11]. It was therefore considered appropriate to assess safety in PD patients rather than healthy controls.

Patients aged 40–80 years were eligible if they were diagnosed with PD according to British brain bank criteria [12], had a Hoehn and Yahr Stage 0–2.5 on medication [13], and stable PD treatment for at least 4 weeks. To exclude major cognitive deficits, a score >25 on the Montreal Cognitive Assessment (MoCA) [14] was required. Patients were carefully selected to exclude medical, psychological, and behavioral problems that may have interfered with their compliance for study participation. Patients provided written informed consent and fulfilled all eligibility criteria.

### Dose Escalation (DE)

In patients 1–3 DE was started at 6 mg GM1 and then increased weekly to 12 mg, 60 mg, followed by increases of 60 mg/week up to 720 mg. TLN was provided as a concentrated liposomal suspension containing cholesterol, sphingomyelin, and the API GM1 in 30 ml vials (180 mg GM1) in a phosphate-buffered solution. TLN was diluted in phosphate-buffered saline with a final concentration of 6 mg/ml GM1. TLN was added to 250 ml NaCl 0.9% and administered with 250 ml/h with a perfusor. The speed of infusion could be increased stepwise up to 370 ml/h if tolerated (absence of adverse events), but reduced if necessary. Three days after the infusion, each weekly dose increase of TLN had to be cleared by an internal safety monitoring committee by assessing clinical symptoms and lab values (S1 Text). The second and third patient could start with the lowest dose with a delay of 1 week after confirmation of safety in the first patient. This 1 + 2 schedule was maintained for each weekly increase to allow for modification of the increasing of doses if needed according to a predefined dose modification matrix. Patients were recruited from the Principal Investigator’s (PI) personal consultation and were chosen because of their reliability, motivation, and ability to appropriately describe and report adverse events. All three patients had two clinical and lab assessments each week during the 14 weeks of the DE phase. The average of the three maximal tolerated doses was defined as maximal suitable dose for the DC part of the trial.

### Dose Consolidation (DC)

**Fig 1:**
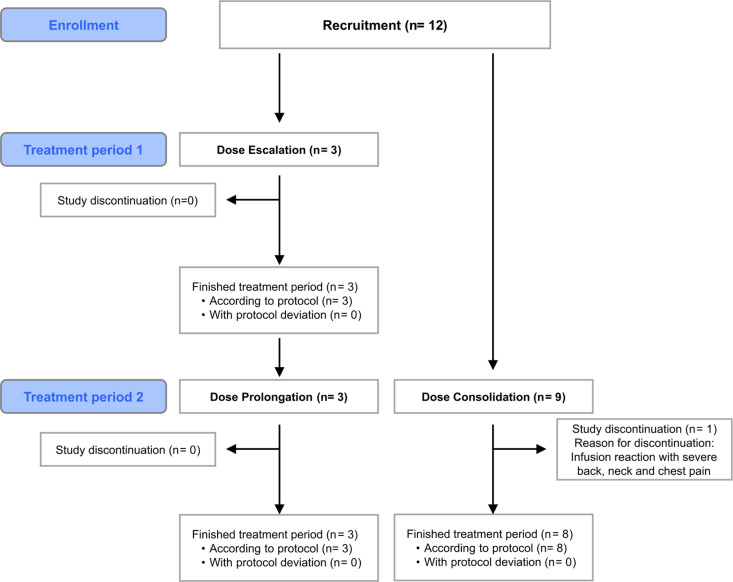
CONSORT diagram of Dose Escalation group (with prolongation) and Dose Consolidation group.

After DE, the initial three and an additional nine patients received weekly infusions of TLN with an API dose of 720 mg and clinical and lab safety assessments over 8 weeks, except for one patient who stopped participation early (Fig 1). A final safety assessment was performed one month after the last administration of TLN.

### Pharmacokinetics

Pharmacokinetic (PK) parameters were determined for GM1 from individual concentration time profiles obtained after the first intravenous infusion administration of 720 mg GM1 in the nine DC patients. Blood samples were drawn before the infusion started and after the start of the infusion (250 ml/h, 370 ml total volume) at 5 min, 1, 4, 8, 24, 48, 72, and 96 h. Samples were immediately centrifuged and frozen at −70 °C. GM1 concentration was measured using liquid chromatography with tandem mass spectrometry.

Non-compartmental PK analysis was performed using Phoenix WinNonlin version 8.0 [Pharsight Corporation, Mountain View, CA, USA] using the intravenous infusion dosing option. PK variables were estimated from the plasma concentration versus time curves: *t*_max_ (time to reach the maximum plasma concentration read directly from the plasma concentration-time curve), *C*_max_ (maximum plasma concentration read directly from the plasma concentration-time curve), AUC_0–∞_ (area under the plasma concentration-time curve from time point zero to infinity, estimated from AUC_0–*t*_ + *C*_*t*_/*λ*_*z*_, where *t* is the last sampling time with a concentration above the limit of quantification and *λ*_*z*_ is the terminal elimination rate constant, estimated by log-linear least squares regression of the plasma concentration versus time data in the terminal phase; AUC_0–∞_ was calculated according to the linear trapezoidal with linear/log interpolation rule), *t*_½_: (apparent terminal half-life calculated as ln2/*λ*_*z*_), CL: (apparent clearance calculated as dose divided by AUC_0–∞_), *V*_*z*_ (apparent volume of distribution calculated as dose divided by *λ*_*z*_ * AUC_0–∞_).

### Outcome measures

The primary outcome was safety defined as the occurrence of adverse events. Adverse events were defined according to the ICH guidelines. In addition, the trial protocol defines that any laboratory value outside the norm range was considered an adverse event, unless the baseline value was already abnormal, and independent of clinical relevance. Safety assessments included a full medical history at baseline, a full general physical, medical, and neurological examination at baseline, a final assessment and last follow-up, and detailed unstructured interviews at each visit and, if needed, by phone. A narrative description of adverse events and their duration, intensity, seriousness, and relation to TLN were recorded by the site investigators. Adverse events were coded by an independent expert with the Medical Dictionary for Regulatory Activities (MedDRA).

Adverse events were considered unrelated to TLN if a plausible other explanation for the observation was available. Otherwise, adverse events without plausible causal relation to TLN but lacking a different explanation were considered to be unlikely related to TLN. Safety data were weekly reviewed by an internal safety board during the DE period. Any out-of-range laboratory result was counted as an adverse event.

Parkinsonian motor signs and non-motor symptoms were further explored weekly using the Movement Disorders Society-Unified Parkinson’s Disease Rating Scale (MDS-UPDRS) [15] parts 1 (non-motor experiences of daily living), 2 (motor experiences of daily living), 3 (motor examination), and 4 (motor complications). The MDS-UPDRS-2 was assessed for best and worst condition in the preceding week in patients with motor fluctuations. The MDS-UPDRS-3 was assessed before and after each administration of TLN and also at baseline and final assessment in a Levodopa Challenge Test (LCT). For the LCT patients paused their dopaminergic medications for at least 12 h before the assessment “off” medication. The usual morning dose of levodopa equivalence plus 50 mg levodopa was then given as liquid formulation of levodopa/benserazide (in one patient levodopa/carbidopa) for the ensuing assessment “on” medication. Levodopa-equivalent daily doses (LEDDs) were noted weekly and calculated according to standard procedures [16]. The number of PD-related non-motor symptoms was assessed with the Non-Motor Symptoms Questionnaire (NMS-Quest) [17] at baseline and weekly during the course of the trial. Questionnaires at baseline and final assessment included the Parkinson’s disease Questionnaire-39 (PDQ-39) [18] for disease-related quality of life, the Epworth Sleepiness Scale [19], the MoCA [14] for mental performance, the Beck Depression Inventory (BDI) [20] to assess mood, and the Starkstein Apathy Scale [21]. Patients were not specifically asked to abstain from novel activities during the study period, but daily routines and general habits did not change.

### Statistical analysis

After completion of data entry, data validation and cleaning were performed. Data analysis was started. All patients enrolled in this study received at least one dose of study medication and were considered in the safety and tolerability analysis.

PK analysis plasma concentrations and PK parameters were analyzed for the nine patients of the consolidation group and reported in a descriptive fashion. Descriptive statistics include arithmetic mean, SD, minimum, median, maximum, geometric mean, and coefficient of variation of arithmetic and geometric means.

Other study outcomes, including scores measuring the Parkinsonian motor signs and non-motor symptoms, disease-related quality of life, LEDD, and mental performance were assessed in the 11 patients who received at least two full-dose infusions and completed at least the MDS-UPDRS at these two visits. For patients included in the escalation part, we used the values assessed at the start of the trial as baseline value (i.e. before the start of the escalation part).

We used summary statistics to describe the outcomes values at baseline, after 8 weeks of treatment, and the observed changes. For each outcome, we reported the mean observed change and the associated 95% Confidence Interval (CI) and assessed the significance of the change from baseline using a paired Wilcoxon signed-rank test. No correction for multiplicity was applied.

Few outcomes, including the blood lab values of cholesterol (total, LDL, HDL, ratio total/HDL), triglycerides, and apolipoprotein B were defined, post-hoc, as additional outcomes. For these outcomes, we used summary statistics to describe the weekly assessed values. At each time point, the significance of the change from baseline was assessed using a paired Wilcoxon signed-rank test.

## Results

### Trial population

There were no screening failures. Twelve patients (including three women) were recruited. At inclusion, the median age was 65 (range 46–75) years old and had had motor parkinsonian signs for 7.9 ± 5.2 (range 2–19, median 6.5) years. Three patients had only akinetic-rigid signs, and nine patients also rest tremor. All patients had been clinically diagnosed with PD. For the patient characteristics, see Table 1.

**Table 1:** Patient characteristics.

Characteristic	Overall, *N* = 12	Dose Consolidation, *N* = 9	Dose Escalation, *N* = 3
Age at registration (years)			
Median (range)	65.0 (46.0–75.0)	65.0 (51.0–75.0)	63.0 (46.0–68.0)
Sex—Female, *n* (%)	3 (25%)	2 (22%)	1 (33%)
Height (cm)			
Median (range)	172.0 (159.0–183.0)	172.0 (160.0–183.0)	174.0 (159.0–182.0)
Weight (kg)			
In	78.5 (60.0–99.0)	78.0 (64.0–99.0)	88.0 (60.0–99.0)
BMI (kg/m^2^)			
Median (range)	26.2 (21.6–32.7)	25.8 (21.6–30.8)	26.6 (23.7–32.7)
Hoehn and Yahr stage at screening (on medication), *n* (%)			
Stage 0 (no signs of disease)	0 (0%)	0 (0%)	0 (0%)
Stage 1 (unilateral involvement only)	3 (25%)	2 (22%)	1 (33%)
Stage 1.5 (unilateral and axial involvement)	5 (42%)	4 (44%)	1 (33%)
Stage 2 (bilateral involvement without impairment of balance)	4 (33%)	3 (33%)	1 (33%)
Stage 2.5 (mild bilateral disease with recovery on pull test)	0 (0%)	0 (0%)	0 (0%)
Drugs			
l-DOPA, *n* (%)	12 (100%)	9 (100%)	3 (100%)
Non-ergot-derived dopamine receptor agonist, *n* (%)	8 (67%)	6 (67%)	2 (67%)
MAO-B inhibitor, *n* (%)	4 (33%)	3 (33%)	1 (33%)
COMT inhibitor, *n* (%)	3 (25%)	3 (33%)	0 (0%)
NMDA agonist, *n* (%)	1 (8.3%)	1 (11%)	0 (0%)
Other, *n* (%)	1 (8.3%)	1 (11%)	0 (0%)
LEDD (mg)			
Median (range)	650.0 (340.0–1,275.0)	750.0 (340.0–1,275.0)	550.0 (375.0–1,010.0)

### Safety

#### Dose Escalation.

Weekly DE of TLN from 6 mg to 720 mg GM1 intravenously (i.v.) at a rate of 250 ml/h was well tolerated without major adverse events. Mild neck and lumbar pain occurred for 15 min in one patient during the second infusion (12 mg) and reappeared very mildly for a few minutes during the third (60 mg) infusion with TLN. Otherwise, DE was unremarkable for all three patients and reached 720 mg with good tolerance. One patient reported transient beneficial effects of TLN treatment on sleep, mood, and general energy levels during DE lasting longer with increasing doses. At the end of the DE, the subjective beneficial effects of the weekly infusions lasted almost a week in this patient.

#### Pharmacokinetics.

**Fig 2:**
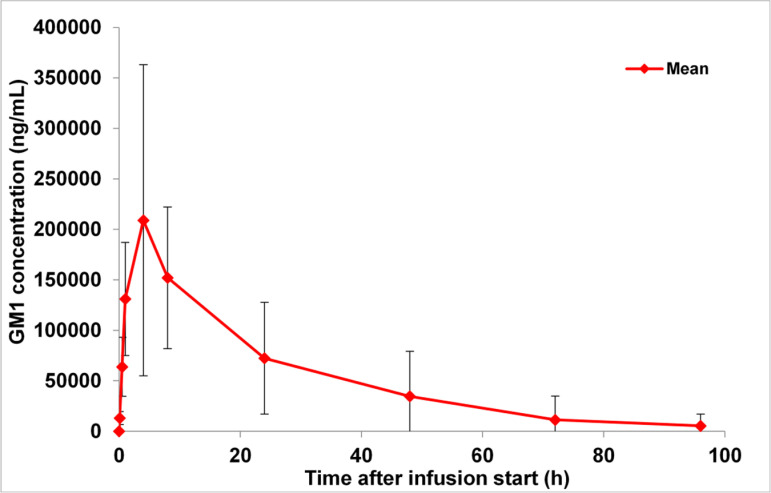
Mean ( ±SD) GM1 plasma concentration-time profiles after intravenous infusion administration of 720 mg GM1 (linear).

After the first intravenous application of TLN with an API dose of 720 mg, repeated blood sampling showed the plasma peak of TLN in the sample taken 4 h after start of the infusion (Fig 2) in all, but one patient in whom the peak was reached at 1 h after start of the infusion. This was the same patient who had the severe acute infusion reaction at the second administration. Median plasma half-life was reached at 12.6 h. PK variables are given in Tables 2 and S1.

**Table 2:** Pharmacokinetic variables for GM1 after intravenously infusion administration of 720 mg GM1 in the Dose Consolidation patients’ group (*n* = 9).

*n* = 9	*C*_BL_ (ng/ml)	*C*_max_ (µg/ml)	*t*_½_ (h)	AUC_0–∞_ (h*µg/ml)	*V*_*z*_ (ml)	CL (ml/h)
Mean	52.8	212	15.0	5,011	4,188	210
SD	30.8	153	6.48	4,035	2,666	113
Minimum	0	84	10.2	1,920	2,020	49
Median	65.4	147	12.6	3,860	3,290	187
Maximum	84.5	582	30.7	14,700	10,000	375

*C*_BL_, baseline plasma concentration before TLN administration; *C*_max_, maximum plasma concentration read directly from the plasma concentration-time curve; *t*_½_, apparent terminal half-life; AUC_0–∞_, area under the plasma concentration-time curve from time point zero to infinity, *V*_*z*_, apparent volume of distribution; CL, apparent clearance.

#### Dose Consolidation.

During the DC phase, acute infusion reactions occurred in six patients shortly after the start of the second (six patients), third (one patient), and fourth (one patient) administration of TLN (see Table 3). One patient with a severe infusion reaction did not receive any further TLN treatment. During all following treatments with TLN, no acute infusion reactions occurred. The maximum dose of GM1,720 mg/week could be maintained throughout the study.

**Table 3:** Acute infusion reactions and the presented symptoms early during the infusion from *n* = 12 patients.

Acute infusion reaction (number of patients)	Second administration	Third administration	Fourth administration
Lower back pain	7^*^	2^*^	1
Neck pain	2^*^	1^*^	0
Itching, urticaria	1	1	1
Nausea, chest pain	2	0	0
Hypotension	1	0	0
Thoracic congestion, plethora	1	0	0
Eye flickering	1	0	0

The number of patients presenting with acute symptoms early during the infusion is given.

*One patient from the Dose Escalation group, all others are from the Dose Consolidation group.

### Adverse events

**Fig 3:**
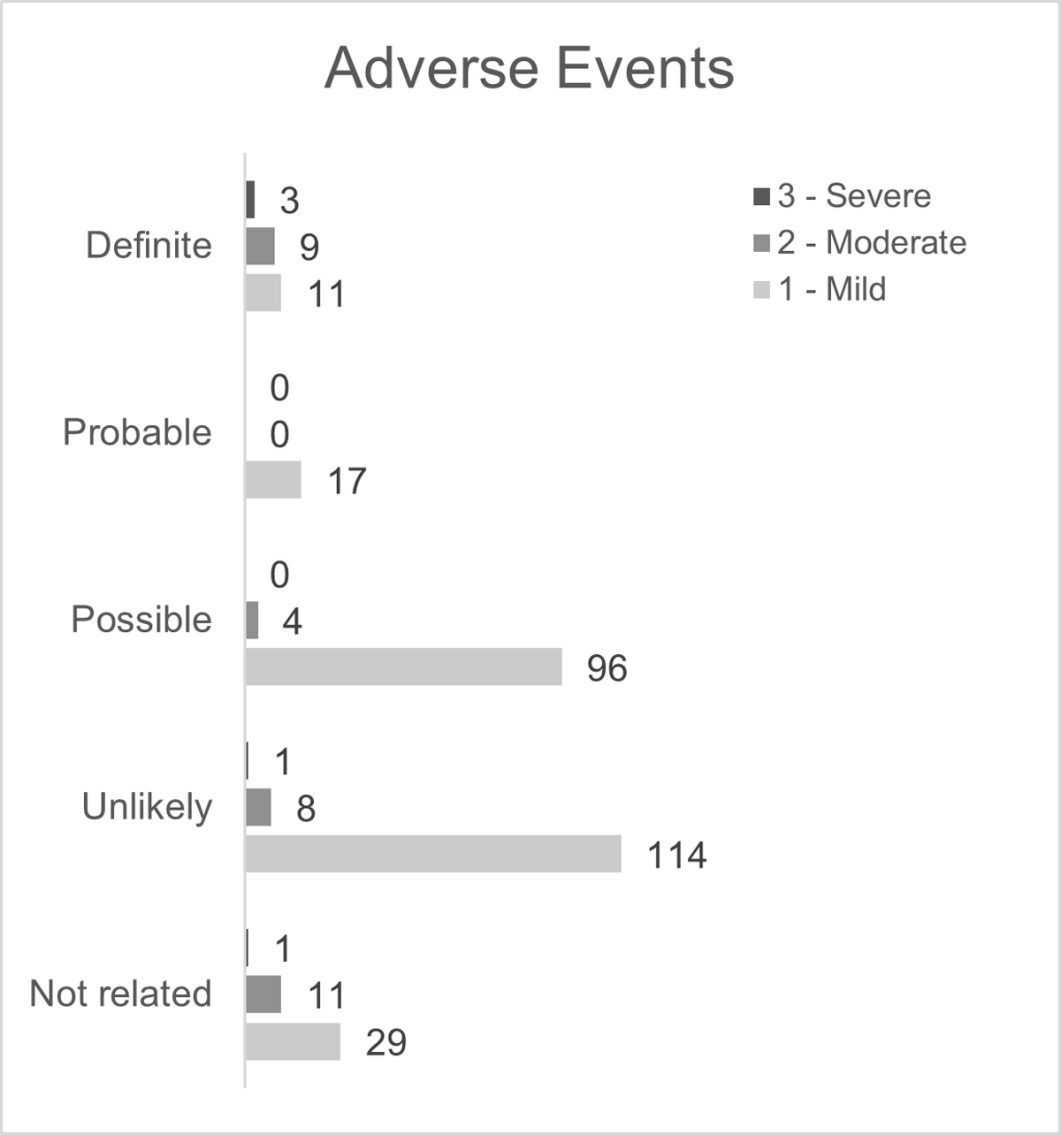
Observed adverse events (*n* = 304) during Dose Escalation and Dose Consolidation with TLN.

Overall, 304, mostly mild adverse events occurred (see Fig 3 and S2 and S3 Tables). No serious adverse event was reported in this study. Twenty-three adverse events were definitely related to the study treatment (S4 Table). The causal relation to the study treatment was considered probable in 17 (all mild), possible in 100, and unlikely in 123 observations. 41 adverse events were unrelated to the study treatment.

Adverse events definitively or probably related to TLN can be separated into a group of immediate mild-to-severe infusion reactions that abated after halting the administration of TLN, and a group of more diffuse, always mild reactions often occurring with some hours delay after TLN infusion. The latter group comprises mild tension headache (*n* = 3), nausea (*n* = 1), and arterial hypertension during emotional tension (*n* = 1) during the infusion, and dizziness (*n* = 6), inner tension and tremulousness (*n* = 2), and tension headache (*n* = 4) occurring within hours after the infusion, spontaneously remitting within less than a day. One patient suffered from a mildly depressed mood during the withdrawal of TLN after the DE phase, partly because he missed the beneficial effects of the weekly infusions. This symptom remitted spontaneously before TLN was resumed in the consolidation phase of the study.

Acute infusion reactions occurred in one patient during DE (second and third administration), and in six patients during DC, always within minutes of the second administration of TLN. In two patients, an acute infusion reaction was observed at the third administration, in one very mildly during the fourth administration. Symptoms occurred after as little as 3 mg of liposomal GM1 was administered and as early as within 1 min after the start of the infusion. Symptoms built up at variable speeds and with mild-to-severe intensity among different patients. All symptoms abated within minutes after stopping the infusion. Infusion could then be resumed at a low speed (10 ml/h) without recurrence of symptoms. In most cases, infusion speed could be increased swiftly up to the planned 250 ml/h except in one patient who required several hours for the second infusion. However, later the patient tolerated administrations of TLN at 250 ml/h without any adverse events. In one patient, the acute infusion reaction at the beginning of the second administration quickly led to severe back, neck, and chest pain. According to the study protocol, the patient had to be removed from the study and could not be re-exposed at a lower infusion rate despite the patients’ explicit request to continue study participation. The acute infusion reactions (Table 3) included lower back, neck, and chest pain as the most common symptoms. Lower back pain was present in all patients with an acute infusion reaction. Lower back pain was very mild in two patients during three administrations and did not require the infusion to be halted. One patient had itching during second, third, and fourth administrations of TLN, and urticaria on the trunk and the thighs (Table 3) during the third infusion. Tryptase was normal before the start of TLN and within 30 min of the appearance of urticaria. Clemastine 2 mg was given i.v., and TLN could be resumed without worsening of symptoms. Urticaria disappeared within a day and did not recur during the following administrations of TLN. After the last occurrence of an acute infusion reaction, patients received 19 (*n* = 1, DE), 6 (*n* = 4), and 4 (*n* = 1) infusions without experiencing an acute infusion reaction. One patient was withdrawn from the study (Fig 1). In the remaining five patients, overall 68 TLN infusions were given without an acute infusion reaction.

Adverse events possibly related to TLN (Table 4) comprised 46 mild out-of-normal range measures of cholesterol, triglycerides, or apolipoprotein B. These observations were considered possibly related to TLN because the study drug contains cholesterol and sphingomyelin. However, lipid assessments were performed in a non-fasting state for which no ranges of normality were available. The lipid levels of all study participants were fluctuating over the study period but did not consistently change except for HDL cholesterol that was slightly but significantly reduced on all visits compared to baseline (see S5 Table). In one patient, one observation of a slightly elevated level of anti-GM1 IgM antibodies was clinically asymptomatic and followed by normal levels in consecutive assessments. Many adverse events possibly related to TLN could also be explained as motor (e.g., muscle cramps, tremor, dysarthria, dysphagia) or non-motor (e.g., fatigue, sleep disorders, constipation, dysphoria) signs and symptoms of PD. During the 4-week pause after the DE phase, one patient reported 25 adverse events of worsening parkinsonian motor and non-motor problems beyond baseline level. This rebound remitted spontaneously to baseline for all reported aspects before TLN was resumed in the DC phase. Mild tension headache, dizziness, nausea, and neck and lumbar pain were noted as possible infusion reaction symptoms.

**Table 4:** Adverse events possibly related to the study treatment (*n* = 100 events from *n* = 12 patients). Categories are based on the judgment of the Principal Investigator.

Category of adverse events	*n*	System organ class terms	Preferred terms of grade 2 adverse events
Abnormal blood lipids	46	Investigations	
Elevated anti-GM1-IgM antibodies	1	Investigations	
Neurologic and psychiatric events	25	Nervous system disorders. Psychiatric disorders. Eye disorders. Renal and urinary disorders. Reproductive system and breast disorders	
Gastrointestinal disorders	7	Gastrointestinal disorders	Nausea
Asthenia and fatigue	5	General disorders and administration site conditions	Fatigue
Possible infusion reactions	7	Injury, poisoning, and procedural complications	Procedural dizziness
Musculoskeletal problems	8	Musculoskeletal and connective tissue disorders	Muscle spasms
Skin disorders	1	Skin and subcutaneous tissue disorders	

System organ class terms and preferred terms were used according to the MedDRA coding system for adverse events.

Adverse events unlikely related to TLN (Table 5) comprised mainly lab results mildly outside of the normal range, which typically normalized spontaneously. While TLN treatment could formally be excluded in these cases, no patterns were observed in recurring lab value deviations. Therefore, out-of-range lab values were considered unlikely related to the treatment in this trial.

**Table 5:** Adverse events unlikely related to the study treatment (*n* = 123 events from *n* = 12 patients). Categories are based on the judgment of the Principal Investigator.

Category of adverse events	n	System organ class terms	Preferred terms of grade 2 and 3 adverse events
Out of range laboratory findings	79	Blood and lymphatic system disorders. Investigations. Metabolism and nutrition disorders	Increased blood potassium, pseudohyperkalemia
Ear, nose, and labyrinth disorder	4	Ear and labyrinth disorders. Respiratory, thoracic and mediastinal disorders	Acute vestibular syndrome
Gastrointestinal disorders	18	Gastrointestinal disorders	Nausea, upper abdominal pain
Infections and infestations	2	Infections and infestations	Post-viral fatigue syndrome
Cardiovascular disorders	2	Investigations. Eye disorders	Amaurosis fugax
Musculoskeletal and connective tissue disorders	9	Musculoskeletal and connective tissue disorders	Back pain
Nervous system and psychiatric disorders	8	Nervous system disorders. Psychiatric disorders	Tension headache
Skin and subcutaneous tissue disorders	1	Skin and subcutaneous tissue disorders	

Two measures of hyperkalaemia (5.5 mmol/l and 5.8 mmol/l) were clinically asymptomatic and occurred without other lab abnormalities; an artifact due to erroneous sample handling (samples were not frozen at −80 °C since this information was accidentally only given to the study team at a later timepoint) was assumed. A self-remitting episode of acute vestibular vertigo was the only severe adverse event considered unlikely to be related to TLN.

System organ class terms and preferred terms were used according to the MedDRA coding system for adverse events.

### Secondary outcome measures

At an early stage, 1 patient discontinued the study and was removed from the efficacy analysis, meaning that the present analysis was performed on 11 patients. At the final assessment, parkinsonian motor signs off medication improved 6.73 points (95% CI, −12, −1.9; *p* = 0.01) on the MDS-UPDRS-3 assessed after 12 h of withdrawal of dopaminergic medication (see Table 6). This corresponded with the observation that pausing medication for the final assessment was much better tolerated than at baseline. With medication (“on” state), no significant change in motor signs was found during the study. TLN treatment did not result in a consistent and clinically relevant immediate beneficial effect on parkinsonian motor signs. Motor complications of dopaminergic treatment (MDS-UPDRS-4) did not change significantly during the study. There were fewer non-motor symptoms at follow-up compared to baseline as assessed with the NMS-Quest (8.64 versus 4.63, *p* = 0.009). Non-motor parkinsonian symptoms (MDS-UPDRS-1) and activities of daily living in the best condition (MDS-UPDRS-2 best) were significantly improved at follow-up compared to baseline (mean difference from baseline −1.91; 95% CI, −3.7, −0.12; *p* = 0.057 and −2.82; 95% CI, −5.1, −0.57; *p* = 0.02; respectively).

**Table 6:** Motor signs and non-motor symptoms, quality of life, and LEDD for the 11 patients.

Assessment (*n* = 11)	Baseline mean ± SD	Follow-up mean ± SD	Mean change from baseline mean (95% CI)	Baseline median (min,max)	Follow-up median (min,max)	Median change from baseline (min,max)	*p*-value (Wilcoxon)
MDS-UPDRS-1	7.82 ± 4.92	5.91 ± 4.11	−1.91 (−3.7, −0.12)	6.00 (2.00, 17.00)	7.00 (1.00, 13.00)	−2.00 (−5.00, 4.00)	0.057
MDS-UPDRS-2 best	8.00 ± 6.91	5.18 ± 7.22	**−2.82** (−5.1, −0.57)	7.00 (0.00, 23.00)	3.00 (0.00, 21.00)	**−2.00 (−9.00, 1.00)**	**0.017**
MDS-UPDRS-2 worst	8.18 ± 7.36	6.45 ± 7.55	−1.73 (−4.4, 0.94)	7.00 (0.00, 25.00)	3.00 (0.00, 22.00)	−1.00 (−9.00, 6.00)	0.122
MDS-UPDRS-3 off	16.09 ± 8.32	9.36 ± 5.55	**−6.73** (−12, −1.9)	17.00 (6.00, 36.00)	8.00 (1.00, 19.00)	**−7.00 (−24.00, 2.00)**	**0.009**
MDS-UPDRS-3 on	6.45 ± 3.42	6.00 ± 4.98	−0.45 (−2.7, 1.8)	5.00 (2.00, 12.00)	5.00 (1.00, 17.00)	−1.00 (−6.00, 5.00)	0.561
MDS-UPDRS-4	2.18 ± 3.52	1.45 ± 3.30	−0.73 (−1.5, 0.01)	1.00 (0.00, 12.00)	0.00 (0.00, 11.00)	0.00 (−3.00, 0.00	0.098
MDS-UPDRS total	34.27 ± 21.79	23.18 ± 16.86	**−11.09** (−18, −4.1)	29.00 (10.00, 90.00)	16.00 (5.00, 58.00)	**−5.00 (−32.00, 0.00)**	**0.006**
NMS-Quest	8.64 ± 3.98	4.36 ± 3.29	**−4.27** (−6.5, −2.1)	8.00 (2.00, 15.00)	5.00 (1.00, 10.00)	**−5.00 (−9.00, 0.00)**	**0.009**
MoCA	28.18 ± 1.33	27.45 ± 2.16	−0.73 (−2.1, 0.62)	29.00 (26.00, 30.00)	28.00 (24.00, 30.00)	−1.00 (−4.00, 3.00)	0.255
PDQ-39-summary index	20.11 ± 8.32	17.21 ± 7.02	**−2.91 (−4.4, −1.4)**	16.25 (9.69, 33.91)	15.52 (9.17, 28.65)	**−2.92 (−6.30, 1.77)**	**0.005**
PDQ-39 activities of daily living	15.91 ± 13.41	8.71 ± 9.21	**−7.20 (−13, −1.6)**	12.50 (0.00, 33.33)	4.17 (0.00, 25.00)	**−4.17 (−20.83, 4.17)**	**0.035**
PDQ-39 bodily discomfort	34.85 ± 17.41	30.30 ± 18.36	−4.55 (−13, 3.5)	41.67 (0.00, 58.33)	33.33 (0.00, 58.33)	0.00 (−25.00, 16.66)	0.309
PDQ-39 cognitive impairment	22.73 ± 18.39	19.32 ± 13.24	−3.41(−14, 7.1)	25.00 (0.00, 56.25)	18.75 (0.00, 50.00)	0.00 (−37.50, 12.50)	0.518
PDQ-39 communication	18.18 ± 22.92	21.97 ± 27.46	3.79(−4.3, 12)	8.33 (0.00, 66.67)	16.67 (0.00, 75.00)	0.00 (−16.67, 25.00)	0.339
PDQ-39 mobility	12.05 ± 7.40	8.86 ± 7.93	−3.18(−7.8, 1.5)	12.50 (2.50, 22.50)	5.00 (0.00, 22.50)	−2.50 (−15.00, 10.00)	0.066
PDQ-39 social support	12.12 ± 10.78	7.58 ± 6.93	−4.55 (−10, 1.3)	8.33 (0.00, 33.33)	8.33 (0.00, 16.67)	0.00 (−25.00, 8.34)	0.203
PDQ-39 stigma	20.45 ± 17.48	21.59 ± 20.98	1.14 (−3.0, 5.3)	12.50 (0.00, 50.00)	18.75 (0.00, 62.50)	0.00 (−6.25, 12.50)	0.588
PDQ-39 emotional wellbeing	24.62 ± 17.33	19.32 ± 12.54	−5.30 (−14, 3.0)	20.83 (0.00, 58.33)	16.67 (8.33, 41.67)	−4.17 (−29.16, 8.33	0.332
Epworth Sleepiness Score	7.73 ± 5.20	7.82 ± 4.85	0.09 (−2.6, 2.8)	6.00 (3.00, 19.00)	8.00 (1.00, 16.00)	0.00 (−7.00, 8.00)	>0.999
Beck Depression Inventory	10.55 ± 4.27	9.27 ± 5.20	−1.27 (−3.8, 1.3)	10.00 (4.00, 19.00)	8.00 (3.00, 21.00)	0.00 (−7.00, 4.00)	0.257
Starkstein Apathy Scale	11.64 ± 4.90	12.00 ± 4.29	0.36 (−1.6, 2.4)	12.00 (3.00, 19.00)	10.00 (4.00, 18.00)	1.00 (−4.00, 6.00)	0.822
LEDD	745.91 ± 330.59	754.09 ± 327.61	8.18 (−7.7, 24)	750.00 (340.00, 1,275.00)	750.00 (340.00, 1,300.00)	0.00 (−10.00, 75.00)	0.423

MDS-UPDRS, Movement Disorders Society-Unified Parkinson’s Disease Rating Scale; NMS-Quest, Non-Motor Symptoms Questionnaire; MoCA, Montreal Cognitive Assessment; PDQ-39, Parkinson’s disease Questionnaire-39; LEDD, Levodopa-Equivalent Daily Dose.

The total MDS-UPDRS (including the MDS-UPDRS-2 in the worst condition and the MDS-UPDRS-3 off medication) improved by 11.1 from baseline to follow-up (95% CI; −18, −4.1; *p* = 0.006).

Quality of life related to PD as measured with the PDQ-39-summary index improved by 2.91 (95% CI; −4.4, −1.4; *p* = 0.005), mainly driven by an improvement of quality of life for activities of daily living. The other subdomains of the PDQ-39 did not improve significantly. No significant change in mood (BDI), motivation (Starkstein Apathy Scale), overall cognition (MoCA), and dopaminergic medication (LEDD) was observed.

During dose-escalation, one patient reported a general effect of well-being and more energy that lasted longer with increasing doses up to 1 week with 720 mg GM1. All patients reported mild-to-moderate reduction of the previously perceived beneficial effects during the month between the last infusion and the follow-up safety assessment. No lasting worsening beyond baseline was observed after one month’s withdrawal of TLN. In the first three patients, total GM1 dose was resumed at 720 mg weekly after a month’s pause following DE; within one or two infusions, the previous level of well-being was re-attained. All patients emphatically requested to prolong TLN treatment at the end of the study. An amendment for study prolongation was therefore submitted.

## Discussion

In this open phase I clinical safety trial, 12 PD patients received weekly infusions with liposomal GM1 for 8–22 weeks. Tolerability was overall very good with no or mild non-specific adverse reactions to the infusion. Some fatigue and dizziness occurred repeatedly in some but not all patients after the infusion and lasted for a few hours, so a causal link to TLN seems probable. However, there were acute infusion reactions in 7 of the 12 patients at the second, third, or fourth exposure to TLN. The intensity was mild-to-moderate and in one case severe leading to exclusion from further study treatment. These reactions were most pronounced at second exposure, consisted mostly of neck and lumbar back pain, and swiftly abated within minutes after TLN infusions were paused. Per trial protocol, IgE measurement were not considered. Still, an allergic IgE-mediated anaphylactic reaction seems highly unlikely as re-exposure did not lead to reappearance of the symptoms but to habituation. We therefore considered a Complement Activation-Related Pseudo-Allergy (CARPA) to the liposome as an explanation for the acute infusion reactions [22]. However, CARPAs typically occur with the first exposure and habituate thereafter, whereas in our patients the first exposure was unproblematic in all 12 patients. Moreover, the clinical manifestation of CARPAs is not typically reported as back pain which was the main manifestation in our patients. The occurrence of the acute infusion reaction only at the second administration is suggestive of a sensitization to TLN, however, the unproblematic tolerance of re-exposure makes autoantibodies to GM1 an unlikely explanation, especially as anti-GM1-IgM levels remained normal throughout the study in these patients and did not increase. Although we cannot explain the mechanism of the observed acute infusion reactions, slowing the infusion rate and possibly progressively increasing doses over the first weeks of treatment with TLN seem to prevent such reactions. Free GM1 has been administered intravenously for over three decades in several neurological disorders for up to 2,500 mg/d without observing acute infusion reactions [23]. In particular, in the randomized placebo-controlled trial with subcutaneous free GM1 patients received a single intravenous loading dose of 1,000 mg GM1. The subsequent subcutaneous administration of GM1 in this study was a route of administration with a much higher risk of triggering an allergic reaction to GM1 than intravenous administration, but no allergic side effects were reported. Therefore, we suspect the acute infusion reactions in our patients to be related to the liposomal carrier of GM1. Further analyses of possible mechanisms for an acute reaction to TLN are ongoing outside of this trial. Other safety assessments of TLN will include lab analyses of blood samples drawn after potential further acute infusion reactions to assess tryptase, thromboxanes, complement factors, activation of factor XII, and measures of basophile activation. However, we try to completely avoid further adverse reactions of this nature by gradually increasing the dose of TLN over the first weeks and by starting the speed of infusion at a very low rate and ramping up as tolerated, especially for the second and third administration of TLN.

Although hyperlipidemia was present in some of our patients already at baseline, there was no consistent increase of lipid levels throughout the trial as had been observed with the administration of very high doses of free GM1 previously [23]. Hyperlipidemia was likely overestimated in our patients as blood was drawn in non-fasting conditions. However, HDL cholesterol was slightly but significantly lowered under TLN treatment which may consist in a vascular risk factor. There were no adverse events related to atherosclerosis in this study, but the observation period was very short. For more conclusive results, lipid levels must be assessed systematically and under standardized fasting conditions in patients treated with TLN in future studies.

Although this is a single-arm phase I safety trial, we gathered exploratory data on the possible therapeutic effects of TLN as a basis for power calculations for a therapeutic trial. In terms of PD symptoms, all patients felt stable or better over the study period. Improvements of sleep quality, motivation, sense of smell, and overall energy were reported, gradually occurring over several weeks. The drug withdrawal for the second LCT was much better tolerated by most patients than the first, and several participants reported that accidental omission of a dose of levodopa was much less perceived than before the treatment with TLN. These encouraging observations are reflected in an improvement of non-motor (MDS-UPDRS-1, NMS-Quest) and motor aspects of experiences of daily living (MDS-UPDRS-2), and of parkinsonian motor signs (MDS-UPDRS-3) resulting in an improvement of disease-related quality of life (PDQ-39). The observed improvements of the MDS-UPDRS-1 and -2 are below the minimal clinically relevant difference of 2.64 and 3.05 for these scales [24]. However, for the motor signs, the minimal clinical improvement on the MDS-UPDRS-3 has been suggested at 3.5 points [25], and our patients improved by 6.73 points. The minimal clinically relevant improvement on the MDS-UPDRS total score has been estimated at 7.1 points [26], whereas our patients improved by 11.09 points. However, for disease-related quality of life measured by the PDQ-39 the minimal clinically important improvement of 4.72% [27] is not reached in our patients. These exploratory observations are encouraging, especially as they uniformly point toward an improvement on different established scales despite a very small number of patients. Further, the putative beneficial effects of TLN might not only be limited to a specific domain but seem to act on multiple clinical aspects of PD, since motor and non-motor symptoms improved significantly. No immediate clinical effect on motor signs was found before and after TLN infusions. A rapid symptomatic beneficial effect of TLN on parkinsonian signs and symptoms is therefore most unlikely. Moreover, as there was no selection of patients based on minimum disease severity, regression to the mean is unlikely to explain the observed improvement in this study. If there is indeed a therapeutic effect of TLN, either a long-term symptomatic effect or a disease-modifying effect seems possible. As the annual progression of PD in its natural course has been estimated at 5.05 points in the untreated and 2.13 points in the treated condition for the sum of the MDS-UPDRS-2 and -3 [28], the observed improvement among our patients may point to a long-term symptomatic or even a neurorestorative effect. Indeed, GM1 can stabilize alpha-synuclein [7] and has a neurorestorative effect on neurons in animal models of PD [29]. Suffering neurons in patients with PD may therefore be rescued by GM1 treatment, and a liposomal formulation may be an effective approach to deliver GM1 into the central and peripheral nervous system. This may explain a gradual and long-lasting reparative effect of TLN despite its short plasma half-life of 12.6 h. However, all this remains speculative at present. As this trial is not designed to show a therapeutic effect of TLN given the open design without placebo control, a placebo effect may explain the observed improvements. An adequately powered randomized placebo-controlled trial of TLN in PD patients is needed to further explore this promising new treatment.

## Supporting information

S1 Checklist Transparent Reporting of Evaluations with Nonrandomized Designs (TREND) statement checklist. (PDF)

S1 Protocol Clinical trial protocol. (PDF)

S1 Text Laboratory analysis list. (PDF)

S1 Table PK data of DC cohort. (PDF)

S2 Table Adverse event list. (XLSX)

S3 Table Adverse event severity per patient (*n* = 304 events from *n* = 12 patients). (PDF)

S4 Table Causal relationship of adverse events to study treatment per patient (*n* = 304 events in *n* = 12 patients). (PDF)

S5 Table Observed lab values and weekly change. (PDF)

S6 Table Clinical assessments data. (PDF)

## Abbreviations

API: Active Pharmaceutical Ingredient
BDI: Beck Depression Inventory
CARPA: Complement Activation-Related Pseudo-Allergy
CI: Confidence Interval
DC: Dose Consolidation
DE: Dose Escalation
GM1: Monosialotetrahexosylganglioside
i.v.: intravenously
LCT: Levodopa Challenge Test
LEDD: Levodopa-Equivalent Daily Dose
MDS-UPDRS: Movement Disorders Society-Unified Parkinson’s Disease Rating Scale
MedDRA: Medical Dictionary for Regulatory Activities
MoCA,: Montreal Cognitive Assessment
NMS-Quest: Non-Motor Symptoms Questionnaire
PD: Parkinson disease
PDQ-39: Parkinson’s disease Questionnaire-39
PK: Pharmacokinetics
TLN: Talineuren
