# Supplementary material for: Safety and tolerability of intravenous liposomal GM1 in patients with Parkinson disease: A single-center open-label clinical phase I trial (NEON trial)
Source: PLoS Med. 2025 May 13;22(5):e1004472. doi: 10.1371/journal.pmed.1004472 (PMC12101738; doi:10.1371/journal.pmed.1004472)
Supplement: S1 Protocol — (PDF) [file pmed.1004472.s002.pdf]

Clinical Trial Protocol

Safety evaluation of intravenous Talineuren (TLN) in patients with Parkinson's disease

NEON

An open-label single arm interventional trial

Study Type:	Clinical trial with Investigational Medicinal Product (IMP)
Study Categorisation:	Risk category C according to the Human Research Act (HRA)
Study Registration:	www.clinicaltrials.gov and Swiss National Clinical Trials Portal (SNCTP) at www.kofam.ch
Study Identifier:	TLN/PD/1
Acronym:	*NEON*
Sponsor:	InnoMedica Schweiz AG<br>Gesellschaftsstrasse 16<br>3012 Bern, Switzerland<br>Tel.: +41 31 311 04 27<br>www.innomedica.com
Investigational Product:	*Talineuren*[redacted] or TLN (Ganglioside GM1)
Protocol Version and Date:	Version 2.0, 11.03.2022

CONFIDENTIAL
The information contained in this document is confidential and the property of the sponsor InnoMedica. The information may not - in full or in part - be transmitted, reproduced, published, or disclosed to others than the applicable Competent Ethics Committee(s) and Competent Authority(is) without prior written authorisation from the sponsor except to the extent necessary to obtain informed consent from those who will participate in the trial.
Copyright © 2021 by InnoMedica

Signature Page

Trial number	TLN/PD/1 *NEON*
Trial Title	Safety evaluation of intravenous Talineuren (TLN) in patients with Parkinson's disease

The sponsor and trial statistician have approved this protocol version, and confirm hereby to conduct the trial according to the protocol, current version of the World Medical Association Declaration of Helsinki, ICH-GCP guidelines and the local legally applicable requirements.

SPONSOR: INNOMEDICA

Bern, 11.3.2022	
Place/Date	Signature (Stefan Halbherr, Country manager)
Bern 11.3.2022	
Place/Date	Signature (Jonas Zeller, CFO)
Abr, 14.3.2022	
Place/Date	Signature (Stefanie Lerch, Medical Affairs)
Bern 11/03/2022	
Place/Date	Signature (Roch-Philippe Charles, Medical Affairs Manager)

TRIAL STATISTICIAN

BERN 14 MAR 2022	
Place/Date	Signature (Corrado Bernasconi, Limites-Research)

PRINCIPAL INVESTIGATOR:

I have read and understood this trial protocol and agree to conduct the trial as set out in this study protocol, the current version of the World Medical Association Declaration of Helsinki, ICH-GCP guidelines and the local legally applicable requirements.

Principal investigator	PD Dr.med. Michael Schüpbach
Site	Neurologisches Institut Konolfingen<br>Burgdorfstrasse 11<br>CH-3510 Konolfingen

Bern, 14. III. 2022	
Place/Date	Signature

Table of Contents

SPONSOR: INNOMEDICA **2**
TRIAL STATISTICIAN **2**
PRINCIPAL INVESTIGATOR: **2**
STUDY SYNOPSIS **6**
ABBREVIATIONS **9**
STUDY SCHEDULE **10**
1. STUDY ADMINISTRATIVE STRUCTURE **12**
1.1 Sponsor 12
1.2 Principal Investigator 12
1.3 Statistician ("Biostatistician") 12
1.4 Laboratory 12
1.5 Monitoring institution 13
1.6 Internal Monitoring Committee (IMC) 13
1.7 Data Safety Monitoring Board (DSMB) 13
2. ETHICAL AND REGULATORY ASPECTS **14**
2.1 Trial registration 14
2.2 Categorisation of trial 14
2.3 Competent Ethics Committee (CEC) 14
2.4 Competent Authorities (CA) 14
2.5 Ethical Conduct of the Study 14
2.6 Declaration of interest 14
2.7 Patient Information and Informed Consent 15
2.8 Participant privacy and confidentiality 15
2.9 Early termination of the trial 15
2.10 Protocol amendments 15
3. BACKGROUND AND RATIONALE **17**
3.1 Background and Rationale 17
3.1.1 Parkinson's disease 17
3.1.2 Gangliosides (GM1 and related metabolites): 17
3.1.3 GM1 and PD: 17
3.1.4 Therapy background 18
3.2 Investigational Product and Indication 18
3.3 Preclinical Evidence 18
3.3.1 Preclinical efficacy 18
3.3.2 Preclinical biodistribution 19
3.3.3 Pharmacokinetics 19
3.3.4 Toxicology 19
3.4 Clinical Evidence to Date 20
3.4.1 Clinical data on GM1 20
3.4.2 Pharmacokinetics for GM1 20
3.4.3 Clinical safety of GM1 20
3.4.4 Clinical safety of TLN's excipients 21
3.4.5 Products containing the API GM1 currently marketed worldwide 21
3.5 Dose Rationale 21
3.6 Explanation for choice of comparator (or placebo) 22
3.7 Risks / Benefits 22
3.8 Justification of choice of study population 22
3.9 Justification for optional continued TLN infusions after the follow-up visit after the dose escalation part 23
4. STUDY OBJECTIVES **24**
4.1 Overall Objective 24
4.2 Primary Objective 24
4.3 Secondary Objectives 24
4.4 Safety Objectives 24
5. STUDY OUTCOMES **25**
5.1 Primary Outcomes 25
5.2 Secondary Outcomes 25
5.3 Other Outcomes of Interest 25
5.4 Safety Outcomes 25
6. STUDY DESIGN **26**
6.1 General study design and justification of design 26

6.1.1 Dose escalation (Part 1) ... 27
6.1.2 Dose consolidation (Part 2) ... 27
6.1.3 Continued TLN infusions ... 27
6.2 Methods of minimising bias ... 28
6.2.1 Randomisation ... 28
6.2.2 Blinding procedures ... 28
6.2.3 Other methods of minimising bias ... 28
7. STUDY POPULATION ... 29
7.1 Eligibility criteria ... 29
7.2 Recruitment and screening ... 29
7.3 Criteria for withdrawal / discontinuation of participants ... 29
8. STUDY INTERVENTION ... 30
8.1 Identity of Investigational Products and administration ... 30
8.1.1 Experimental Intervention treatment ... 30
8.1.2 Control Intervention ... 30
8.1.3 Packaging, Labelling and Supply (re-supply) ... 30
8.1.4 Storage Conditions ... 31
8.2 Administration of experimental and control interventions ... 31
8.2.1 Experimental Intervention ... 31
8.2.2 Control Intervention Experimental Intervention ... 32
8.3 Dose modifications ... 32
8.4 Compliance with study intervention ... 33
8.5 Data Collection and Follow-up for withdrawn participants ... 33
8.6 Trial specific preventive measures ... 33
8.7 Concomitant Interventions (treatments) ... 34
8.8 Study Drug Accountability ... 34
8.9 Return or Destruction of Study Drug ... 34
9. ASSESSMENTS ... 35
9.1 Table of trial procedures and assessments ... 35
9.2 Assessments of outcomes ... 37
9.2.1 Assessment of primary outcome ... 37
9.2.2 Assessment of secondary outcomes ... 38
9.2.3 Assessment of other outcomes of interest ... 39
9.2.4 Assessment of safety outcomes ... 39
9.2.5 Assessments in participants who prematurely stop the study ... 39
9.3 Procedures at each visit ... 39
9.3.1 Visit 1: Screening (between day -28 until day -1) ... 39
9.3.2 Visit 2: Baseline (day 0) ... 40
9.3.3 Visit 3, 5, 7, 9, 11 and every other visit until visit 29: Treatment ... 40
9.3.4 Visit 4, 6, 8, 10, 12 and every other until visit 28: Assessment ... 40
9.3.5 Visit 30: Final assessment (7 days after last TLN infusion) ... 40
9.3.6 Visit 31: Follow-up (28 days after TLN infusion) ... 40
9.3.7 Visit 1: Screening (between day -28 until day -1) ... 41
9.3.8 Visit 2: Baseline (day 0) ... 41
9.3.9 Visit 3: Treatment (day 1 - this visit takes place at the CI Bern) ... 41
9.3.10 Visit 4 – 7: PK (days 2 to 5 - these visits take place at the CI Bern) ... 41
9.3.11 Visit 8 – 14: Treatment (days 8, 15, 22, 29, 36, 43 & 50) ... 41
9.3.12 Visit 15: Final assessment (7 days after last TLN infusion) ... 41
9.3.13 Visit 16: Follow-up (28 days after last TLN infusion) ... 42
9.3.14 Visit 0: “Re-consent” (day 0) ... 42
9.3.15 Visit 1-8: Treatment (day 1, 8, 15, 22, 29, 36, 43 & 50) ... 42
9.3.16 Visit 9: Final assessment (7 days after last TLN infusion) ... 42
9.3.17 Visit 10: Follow-up (28 days after last TLN infusion) ... 42
10. SAFETY (DRUG STUDY) ... 43
10.1 Definition and assessment of (serious) adverse events and other safety related events ... 43
10.1.1 Reporting of SAE and other safety related events ... 45
10.1.2 Follow up of (S)AEs ... 45
11. STATISTICAL METHODS ... 46
11.1 Hypothesis ... 46
11.2 Determination of Sample Size ... 46
11.3 Statistical criteria of termination of trial ... 46
11.4 Planned Analyses ... 46

11.4.1 Datasets to be analysed, analysis populations 46
11.4.2 Primary Analysis 46
11.4.3 Secondary Analyses 46
11.4.4 Interim analyses 47
11.4.5 Safety analysis 47
11.4.6 Deviation(s) from the original statistical plan 47
11.5 Handling of missing data and drop-outs 47
12. QUALITY ASSURANCE AND CONTROL 48
12.1 Data handling and record keeping / archiving 48
12.1.1 Case Report Forms 48
12.1.2 Specification of source documents 48
12.1.3 Record keeping / archiving 48
12.2 Data management 48
12.2.1 Data Management System 48
12.2.2 Data security, access and back-up 48
12.2.3 Analysis and archiving 49
12.2.4 Electronic and central data validation 49
12.3 Monitoring 49
12.4 Audits and Inspections 49
12.5 Confidentiality, Data Protection 49
12.6 Storage of biological material and related health data 49
13. PUBLICATION AND DISSEMINATION POLICY 50
14. FUNDING AND SUPPORT 50
14.1 Funding 50
14.2 Other Support 50
15. INSURANCE 50
16. REFERENCES 51
17. APPENDICES 53

STUDY SYNOPSIS

**Sponsor**	InnoMedica AG Switzerland
**Study Title:**	Safety evaluation of intravenous Talineuren (TLN) in patients with Parkinson's disease
**Short Title / Study ID:**	TLN/PD/1 (*NEON*)
**Protocol Version and Date:**	Version 2.0, 11.03.2022
**Trial registration:**	The sponsor is registered at www.clinicaltrials.gov under the following number NCT04976127 and will be registered on the Swiss National Clinical Trials Portal (SNCTP) at www.kofam.ch
**Study category and Rationale**	Clinical trial with Investigational Medicinal Product (IMP).<br>The IMP is a medication without marketing authorization in Switzerland nor anywhere in the world.<br>According to the Swiss Human research act (HRA) and its corresponding Ordinance (ClinO) on clinical trials, this trial is classified as category C.
**Clinical Phase:**	Phase I, Safety evaluation
**Background and Rationale:**	The pharmacologically active ingredient of TLN is the membranal lipid Monosialotetrahexosylganglioside or GM1. TLN consists of GM1 associated to a proprietary lipid formulation assembled together as liposomes. Free injections of GM1 have shown to improve the UPDRS-score and therefore the condition of patients with Parkinson's disease. TLN has been developed to improve the delivery of GM1 and its bioavailability and therefore improve the care of Patients with Parkinson's disease.
**Objective(s):**	The primary objective is:<br>• To demonstrate the safety of TLN administration intravenously in patients with Parkinson's disease.<br>Secondary objectives are:<br>1. Determination of the maximal suitable dose based on safety profile and preliminary efficacy.<br>2. Determination of the PK profile.
**Outcome(s):**	<u>Primary outcome: Safety, measured as:</u><br>• Occurrence of AEs<br>• Occurrence of SAEs<br><u>Secondary outcomes:</u><br>• Parkinson disease (PD) patients' condition assessed by: Levodopa challenge test (LCT), MDS-UPDRS, PDQ-39, ESS and change in Parkinson's medication (LEDD).<br>• Patients' general/mental condition assessed by: BDI, SAS, NMSQuest and MoCA.<br>• PK analyses
**Study design:**	Open-label, single ascending dose escalation followed by a multiple administration dose at the maximal suitable dose.

**Inclusion / Exclusion criteria:**	**Inclusion criteria:**<br>1. Informed consent as documented by signature.<br>2. Male and female subjects, aged 40 to 80 years.<br>3. Confirmed PD according to British brain bank criteria.<br>4. Hoehn and Yahr Stage 0 – 2.5 on medication.<br>5. Stable PD treatment for a month at least.<br>6. Absence of dementia confirmed by cognitive testing (MoCA >25).<br>**Exclusion criteria:**<br>1. Contraindications to the class of drugs under study, e.g., known hypersensitivity or allergy to class of drugs or the investigational product.<br>2. Women who are pregnant or breast feeding, or planning to become pregnant during the course of the trial or in the 12 weeks following the trial.<br>3. Lack of safe contraception, defined as:<br>• Female participants of childbearing potential, not willing to use double method of contraception (hormonal and mechanical) for the entire study duration. Female participants who are surgically sterilised / hysterectomised or post-menopausal for longer than 2 years are not considered as being of child bearing potential.<br>• Male participants, not using and not willing to use a medically reliable method of contraception for the entire study duration, such as condoms or who are not using any other method considered sufficiently reliable by the investigator in individual cases.<br>4. Other clinically significant concomitant disease states (e.g., renal failure, hepatic dysfunction, cardiovascular disease etc.) that is not under stable control.<br>5. Known or suspected non-compliance, drug or alcohol abuse.<br>6. Inability to follow the procedures of the trial, e.g., due to language problems, psychological disorders etc. of the participant.<br>7. Participation in another trial with an investigational drug within the 30 days preceding and during the present trial.<br>8. Previous enrolment into the current trial.<br>9. Enrolment of the investigator, his/her family members, employees and other dependent persons.<br>10. Subject has an atypical parkinsonian syndrome or secondary parkinsonism.<br>11. Patients with comorbidity that may interfere with the course of the trial.<br>12. Patients who are not considered to be eligible to participate in clinical trial by the investigator.

**Measurements and procedures:**	Monocentric clinical trial phase I conducted with ascending escalating doses with the option of treatment prolongation, followed by a dose consolidation part with weekly TLN infusions in patients affected by PD.<br><br><u>Examinations before first trial treatment:</u><br>Medical history, Clinical Assessment, vital signs, routine blood analysis, pregnancy test (if indicated).<br>Assessment of baseline symptoms and LCT, MDS-UPDRS, PDQ-39 and ESS. Assessment of patient's psychological and cognitive condition: BDI, SAS, NMSQuest and MoCA.<br><br><u>Examinations during treatment:</u><br>Assessment of AE and SAE. Vital signs, MDS-UPDRS and SAS, NMSQuest. LEDD, routine blood analysis.<br><br><u>Examinations after last trial treatment:</u><br>Assessment of AE and SAE. Clinical assessment and vital signs, routine blood analysis.<br>Assessment of final symptoms by LCT, MDS-UPDRS, PDQ-39 ESS and LEDD. Assessment of patient's psychological and cognitive condition: BDI, SAS, NMSQuest and MoCA.
**Study Product / Intervention:**	*Talineuren*[redacted] (TLN)
**Control Intervention:**	Not applicable
**Number of Participants with Rationale:**	12 patients in total:<br>- 3 for the dose ascending part<br>- 9 for the multiple dose administration part
**Study Duration:**	8 to 12 months
**Study Schedule:**	First patient in: Q4 2021<br>Last patient in: Q2 2022<br>Last patient out: Q4 2022
**Investigator(s):**	PD Dr. med. Michael Schüpbach
**Study Centre(s):**	Neurologisches Institut Konolfingen<br>Burgdorfstrasse 11<br>CH-3510 Konolfingen
**Statistical Considerations:**	The analysis will be exploratory and primarily employ descriptive statistical methods. Inferential methods will be used to highlight interesting aspects of the data. Unless otherwise specified statistical tests will be two sided and conducted at the 5% significance level. Corresponding 95%-confidence intervals will be presented and no correction for multiplicity will be applied.
**GCP Statement:**	This trial will be conducted in compliance with the protocol, the current version of the Declaration of Helsinki, the ICH-GCP as well as all national legal and regulatory requirements.

ABBREVIATIONS

AE	*Adverse Event*
[redacted]	[redacted]
API	*Active Pharmaceutical Ingredient*
ASR	*Annual Safety Report*
AUC	*Area Under the Concentration Time Curve*
BASEC	*Business Administration System for Ethical Committees*
BD	*Biodistribution*
BDI	*Beck’s Depression Inventory*
b.i.d.	*Bis in die (twice a day)*
CA	*Competent Authority*
CEC	*Competent Ethics Committee*
CI Bern	*Clinical Investigation Bern*
ClinO	*Ordinance on Clinical Trials in Human Research*
eCRF	*Electronic Case Report Form*
DB	*Database*
DSMB	*Data Safety Monitoring Board*
EDC	*Electronic Data Capture*
ESS	*Epworth Sleepiness Scale*
GBS	*Guillain-Barré Syndrome*
GCP	*Good Clinical Practice*
GM1	*Monosialotetrahexosylganglioside*
HRA	*Human Research Act*
IB	*Investigator’s Brochure*
IMC	*Internal Monitoring Committee*
IMP	*Investigational Medicinal Product*
i.m.	*Intramuscular (injection)*
ISF	*Investigator Site File*
i.v.	*Intravenous (injection)*
LEDD	*Levodopa Equivalent Daily Dose*
LCT	*Levodopa Challenge Test*
MDS - UPDRS	*Movement Disorder Society’s Unified Parkinson's Disease Rating Scale*
MAD	*Multiple Administration Dose*
MSD	*Maximal Suitable Dose*
MoCA	*Montreal Cognitive Assessment*
NMSQuest	*Non-Motor Symptoms Questionnaire*
PDQ39	*Parkinson's Disease Questionnaire 39*
PI	*Principal Investigator*
PK	*Pharmacokinetics*
SAD	*Single Ascending Dose*
SAE	*Serious Adverse Event*
SAS	*Starkstein Apathy Scale*
SMP	*Safety Management Plan*
SNCTP	*Swiss National Clinical Trials Portal*
s.c.	*Subcutaneous (injection)*
SOP	*Standard Operating Procedure*
SUSAR	*Suspected Unexpected Serious Adverse Reaction*
TLN	*Talineuren*
TMF	*Trial Master File*
UPN	*Unique Patient Number*

STUDY SCHEDULE

Trial Periods	Screening	Baseline	Treatment (T)	Assessment (A)	T	A	T	A	T	A	T	A	T	A	T	A	T	A	T	A	T	A	T	A	T	A	T	A	T	Final assessment	Follow-up
Visit	1	2	3	4	5	6	7	8	9	10	11	12	13	14	15	16	17	18	19	20	21	22	23	24	25	26	27	28	29	30	31
Time (weeks)	-4 to -1	1	1	1	2	3	3	4	4	5	5	6	6	7	7	8	8	9	9	10	10	11	11	12	12	13	13	14	14	+1	+4
Time (days)	-28 to -1	0	1	4	8	11	15	18	22	25	29	32	36	39	43	45	49	52	56	59	63	66	70	73	77	80	84	87	91	+7	+28
Patient information & consent	x																														
In- /Exclusion Criteria	x																														
Pregnancy Test	x																														
Medical History	x																														
Clinical Assessment	x																													x	x
Vital Signs	x	x	x	x	x	x	x	x	x	x	x	x	x	x	x	x	x	x	x	x	x	x	x	x	x	x	x	x	x	x	x
MoCA	x																													x	
Lab tests	x	x		x		x		x		x		x		x		x		x		x		x		x		x		x		x	x
Levodopa Challenge Test		x																												x	
TLN Infusion			6		12		60		120		180		240		300		360		420		480		540		600		660		720		
MDS-UPDRS			x		x		x		x		x		x		x		x		x		x		x		x		x		x	x	x
PDQ-39		x																												x	
ESS		x																												x	
BDI		x																												x	
SAS		x																												x	
NMSQuest		x		x		x		x		x		x		x		x		x		x		x		x		x		x		x	
LEDD		x		x		x		x		x		x		x		x		x		x		x		x		x		x		x	x
Adverse events recording			x	x	x	x	x	x	x	x	x	x	x	x	x	x	x	x	x	x	x	x	x	x	x	x	x	x	x	x	x

Table 1: Schedule for dose escalating part (part 1)

Trial Periods	Screening	Baseline	Treatment (T)	PK 24h	PK 48h	PK 72h	PK 96h	T	T	T	T	T	T	T	Final assessment	Follow-up
Visit	1	2	3	4	5	6	7	8	9	10	11	12	13	14	15	16
Time (weeks)	-4 to -1	1	1	1	1	1	1	2	3	4	5	6	7	8	+1	+4
Time (days)	-28 to -1	0	1	2	3	4	5	8	15	22	29	36	43	50	+7	+28
Patient information & consent	x															
In- /Exclusion Criteria	x															
Pregnancy Test	x															
Medical History	x															
Clinical Assessment	x														x	x
Vital Signs	x	x	x					x	x	x	x	x	x	x	x	x
MoCA	x														x	
Lab tests	x	x						x	x	x	x	x	x	x	x	x
Levodopa Challenge Test		x													x	
TLN Infusion			x					x	x	x	x	x	x	x		
Blood collection (PK)			x	x	x	x	x									
MDS-UPDRS			x					x	x	x	x	x	x	x	x	x
PDQ-39		x													x	
ESS		x													x	
BDI		x													x	
SAS		x													x	
NMSQuest		x						x	x	x	x	x	x	x	x	
LEDD		x						x	x	x	x	x	x	x	x	x
Adverse events recording			x	x	x	x	x	x	x	x	x	x	x	x	x	x

Table 2: Schedule for dose consolidating part (part 2)

Trial Periods	Re-consent	T	T	T	T	T	T	T	T	Final assessment	Follow-up
Visit	0	1	2	3	4	5	6	7	8	9	10
Time (weeks)	-4 to -1	1	2	3	4	5	6	7	8	+1	+4
Time (days)	-28 to 0	1	8	15	22	29	36	43	50	+7	+28
Patient information & consent	x*										
Clinical Assessment										x	x
Vital Signs		x	x	x	x	x	x	x	x	x	x
MoCA										x	
Lab tests		x	x	x	x	x	x	x	x	x	x
Levodopa Challenge Test										x	
TLN Infusion		x	x	x	x	x	x	x	x		
Blood collection (PK)											
MDS-UPDRS		x	x	x	x	x	x	x	x	x	x
PDQ-39										x	
ESS										x	
BDI										x	
SAS										x	
NMSQuest		x	x	x	x	x	x	x	x	x	
LEDD		x	x	x	x	x	x	x	x	x	x
Adverse events recording		x	x	x	x	x	x	x	x	x	x

Table 3: Schedule of assessment prolongation (optional)

1. STUDY ADMINISTRATIVE STRUCTURE

1.1 Sponsor

InnoMedica AG Switzerland
Gesellschaftsstrasse 16
3012 Bern
Tel.: +41 31 311 04 27
Website: www.innomedica.com

As sponsor, Innomedica is responsible for the following areas:
- trial design
- data collection
- monitoring
- data analyses
- reporting

The following tasks are outsourced to collaborating partners:
- statistical analysis
- data management
- safety re-assessments on behalf of InnoMedica (safety management)

All collaborating partners are qualified according to ICH-GCP to perform the requested tasks.

1.2 Principal Investigator

PD Michael Schüpbach, MD
Facharzt für Neurologie FMH

Site:
Neurologisches Institut Konolfingen
Burgdorfstrasse 11
3510 Konolfingen
[redacted]

Satellite site (mainly for PK sampling):
Clinical Investigation (CI) Bern
Department of Clinical Research
sitem-insel
Freiburgstrasse 3
3010 Bern
[redacted]

1.3 Statistician ("Biostatistician")

Corrado Bernasconi, MD, PhD
Limites Medical Research Ltd.
Via Fornasette 2
6833 Vacallo
[redacted]

1.4 Laboratory

Routine lab assays:
Viollier AG
Hagmattstrasse 14
4123 Allschwil
[redacted]
Website: https://www.viollier.ch

Pharmacokinetics:
Swiss BioQuant AG
Kägenstrasse 18
4153 Reinach
[redacted]
Website: http://swissbioquant.com/

1.5 Monitoring institution

InnoMedica AG Switzerland
Gesellschaftsstrasse 16
3012 Bern
[redacted]
Website: www.innomedica.com

1.6 Internal Monitoring Committee (IMC)

The IMC is the executive board concerning decisions during the trial (e.g., dose escalation procedure) and consists of the PI, the manager medical affairs, and an external Medical Doctor as an independent medical monitor. The sponsor guarantees the independence of the later as he/she has an important role within the IMC. Every discussion/decision of the IMC will be documented and filed in the trial master file (TMF). Decisions to pursue the trial for each patient will be taken unanimously by the three members. This guaranties that the external member's opinion will have the same value as of the other members.
In principle, the IMC will meet on a weekly basis during the treatment phase in order to evaluate all safety data reported until that timepoint. All data will be verified and SAEs re-assessed before.

1.7 Data Safety Monitoring Board (DSMB)

The Data Safety Monitoring Board (DSMB) is an advisory board, that is independent from the sponsor and which has no competing interests. Its main objective is to ensure the integrity and safety of participants of the NEON trial by providing unbiased review of raw (not aggregated) clinical trial data. The DSMB consists of 3 independent experts in the field. More details are provided in the trial specific DSMB charter, that serves as guideline of operating principles for DSMB members to oversee patient safety and data quality, to review trial efficacy and progress and to ensure the validity and integrity of the trial on a regular basis.
The DSMB will meet after dose level 5, 10 and 15 during part 1 of the trial, after the maximal suitable dose (MSD) has been defined and after last patient last visit.

2. ETHICAL AND REGULATORY ASPECTS

Before the trial will be conducted, the protocol, the patient information and consent forms as well as other trial-specific documents shall be submitted to the Bernese cantonal ethics committee (CEC) and Swissmedic (CA). The decision of the CEC and CA concerning the conduct of the trial will be made in writing to InnoMedica before commencement of this trial. The clinical trial can only begin once approval from all required authorities has been received. Any additional requirements imposed by the authorities shall be implemented.

2.1 Trial registration

The study will register the trial at www.clinicaltrials.gov and the Swiss National Clinical Trials Portal (SNCTP) at www.kofam.ch.

2.2 Categorisation of trial

This is a clinical trial with Investigational Medicinal Product (IMP) without marketing authorization in Switzerland nor anywhere in the world. According to the Swiss HRA and its corresponding Ordinance ClinO on clinical trials, this trial is classified as category C.

2.3 Competent Ethics Committee (CEC)

The sponsor ensures that approval from the CEC is sought for this trial.
The responsible investigator should not implement any deviation from, or changes of the protocol without agreement by the sponsor and prior review and documented approval/favourable opinion from the CEC of an amendment, except where necessary to eliminate an immediate hazard to trial subjects, or when the change(s) involves only logistical or administrative aspects of the trial (non-substantial amendments).
If immediate safety and protective measures have to be taken during the conduct of the trial, Innomedica will notify the CEC of these measures, and of the circumstances necessitating them, within 7 days.
Premature trial end or interruption of the trial is reported within 15 days. The regular end of the trial is reported to the CEC within 90 days, the final trial report shall be submitted within one year after trial end. Amendments are reported according to section **2.10 Protocol amendments**.
Completion of a clinical trial is marked by the last participant's final follow-up visit.

2.4 Competent Authorities (CA)

The sponsor will obtain approval from Swissmedic before the start of the clinical trial as this trial is classified as risk category C.
If immediate safety and protective measures have to be taken during the conduct of the trial, InnoMedica will notify Swissmedic of these measures, and of the circumstances necessitating them, within 7 days.
Premature trial end or interruption of the trial is reported within 15 days. The regular end of the trial is reported to Swissmedic within 90 days, the final trial report shall be submitted within one year after trial end. Amendments are reported according to section **2.10 Protocol amendments**.
Completion of a clinical trial is marked by the last participant's final follow-up visit.

2.5 Ethical Conduct of the Study

The trial will be carried out in accordance to the protocol and with principles enunciated in the current version of the Declaration of Helsinki, the guidelines of Good Clinical Practice (GCP) issued by ICH, the Swiss Law and Swiss CA's requirements. The CEC and CA will receive annual safety and interim reports and be informed about trial stop/end in agreement with local requirements.

2.6 Declaration of interest

InnoMedica is the sponsor, the manufacturer and patent holder of Talineuren (TLN).
The other parties involved declare no conflict of interest regarding the present trial. The principal investigators and the clinical investigation Bern are compensated for their work on a per-patients basis (investigator's grants). The biostatistician is compensated by hours of his work. The data management performed by the CTU Bern is compensated by hours of their work and according to the database

size. They are not financially involved in the company Innomedica or compensated by any other means.

2.7 Patient Information and Informed Consent

This trial will not include vulnerable patients nor healthy volunteers. The investigators will explain to each candidate the nature of the trial, its purpose, the procedures involved, the expected duration, the potential risks and benefits and any discomfort it may entail. Each candidate will be informed that the participation in the trial is voluntary and that he/she may withdraw from the trial at any time and that withdrawal of consent will not affect his/her subsequent medical assistance and treatment.
The candidate must be informed that his/her medical records may be examined by authorised individuals other than their treating physician.
All potential participants for the trial will be provided with a participant information sheet and a consent form describing the trial and providing sufficient information for her/him to make an informed decision about their participation in the trial. They will have up to 28 days to decide if they wish to enter the trial or not.
The formal consent of a participant, using the approved consent form, will be obtained before the participant is submitted to any trial procedure. The patient will be informed about pre-screening assessments needed for the evaluation of the eligibility criteria prior to trial enrolment. Patients will thus know, that if pre-screening results do not meet eligibility criteria, they might not be enrolled to the trial. In that case, this will be judged as screening failure.
The participant should read and consider the statement before signing and dating the informed consent form, and should be given a copy of the patient information and informed consent. The consent form must also be signed and dated by the investigator (or his/her designee) at the same time (or before) as the participant signs, and it will be retained as part of the trial records.

After having completed the regular treatment schedule including the follow-up visit, all patients from part 1 (patient 1 to 3) will have the opportunity to continue receiving weekly TLN infusions for an additional 8 weeks according. All patients wishing to prolong their TLN treatment will be provided with an amended patient information and consent form describing the additional treatments and assessments. Written informed consent will be obtained before the participant is submitted to any amended trial procedure.

2.8 Participant privacy and confidentiality

The investigator affirms and upholds the principle of the participants' right to privacy and that they shall comply with applicable privacy laws. Especially, anonymity of the participants shall be guaranteed when presenting the data at scientific meetings or publishing them in scientific journals. Individual subject medical information obtained as a result of this trial is considered confidential and disclosure to third parties is prohibited. Subject confidentiality will be further ensured by utilising subject identification code numbers to correspond to treatment data in the computer files.
For data verification purposes, authorised representatives of InnoMedica, the CA, or the CEC may require direct access to parts of the medical records relevant to the trial, including participants' medical history.

2.9 Early termination of the trial

InnoMedica may terminate the trial prematurely according to certain circumstances, for example:

- ethical concerns,
- insufficient participant recruitment,
- when the safety of the participants is doubtful or at risk, respectively,
- alterations in accepted clinical practice that make the continuation of a clinical trial unwise,
- early evidence of benefit or harm of the experimental intervention

2.10 Protocol amendments

InnoMedica may initiate protocol modifications in agreement with the principal investigator if the data acquired justify it.
Substantial amendments are only implemented after approval of the CEC and CA respectively.
Under emergency circumstances, deviations from the protocol to protect the rights, safety and well-being of the study participants may proceed without prior approval of the sponsor and the CEC/CA.

Such deviations shall be documented and reported to InnoMedica and the CEC/CA as soon as possible.
All non-substantial amendments are communicated to the CA as soon as possible if applicable and to the CEC within the Annual Safety Report (ASR).

3. BACKGROUND AND RATIONALE

3.1 Background and Rationale

3.1.1 Parkinson's disease

Parkinson's disease (PD) is a neurodegenerative disease affecting progressively different parts of the brain. In particular, the dopamine-producing neurons of the *substantia nigra* undergo apoptosis resulting in clinical signs of the movement disorder defining PD. However, PD results in more widespread signs and symptoms than movement disorders, including disorders of mood, cognition, and vegetative functions. The exact causes of PD remain elusive, however, progressive neurodegeneration with a caudo-rostral gradient has been linked to different mechanisms:

1. Aging of the membranes (lipidic composition change[1] and most notably lipid rafts)
2. Protein aggregation (e.g.: alpha-synuclein[2])
3. Neuroinflammation[3]

A feature commonly observed *post-mortem* in PD patients is the presence of Lewy bodies (protein aggregates rich in alpha-synuclein) in the affected neurons.

According to the Parkinson Switzerland association, roughly 15'000 persons with PD live in Switzerland. PD is one of the most common neurodegenerative diseases with almost 10 million people affected worldwide. Most diagnoses are made after 60 years of age underlining the importance of aging and/or degenerative processes of the brain in the disease. Less than 10% of the cases are considered as genetically inheritable but this might be underestimated due to the fact that the disease is most likely multifactorial and age related.

The disease can take many forms and is very variable between patients. The main motor symptoms include:

1. Bradykinesia (slowness of movements) as the hallmark and mandatory clinical sign, and
2. rigidity of the limbs,
3. resting tremor,
4. impairment of gait and balance.

Tremor is present only in a part of the patients, and the clinical manifestations may vary from patient to patient. In addition to the listed motor signs, vegetative signs and symptoms are common (constipation, urinary urgency, sleep disorders etc.) as well as changes in mood, cognition and sense of smell. Signs and symptoms that are a consequence of the dopaminergic deficit may improve with medications containing levodopa; this substitution, however, covers only a part of the signs and is hampered by fluctuations in its effects over the day, resulting in motor fluctuations ("on" versus "off") and excessive involuntary movements (dyskinesia). Those clinical manifestations of PD that are due to neurodegeneration of brain structures other than the dopamine system are much more difficult to treat and may affect quality of life of patients even more that the motor signs. Typically, the levodopa-resistant signs and symptoms develop later during the course of PD, but the clinical manifestation and course over time of PD varies greatly among patients. Interestingly, life expectancy of affected patients is not drastically reduced. The impact, however, is huge on the quality of life for the patients and their relatives and caregivers.

3.1.2 Gangliosides (GM1 and related metabolites):

Lipids and more specifically membranal lipids are essential for neuronal function. Several families of lipids compose neuronal membranes, among them the gangliosides. They are complex glycosphingolipids composed of a ceramide backbone, an oligosaccharide polar group containing neutral sugars (such as glucose or galactose), and one or more negatively charged sugars (sialic acid). The first member of this family, GM1 is a glycosphingolipid ubiquitous in the brain that belongs to the subgroup of gangliosides together with GD1a, GD1b and GT1b[4]. GM1 contains one sialic acid residue and displays an amphiphilic behaviour that allows to establish strong both hydrophobic and hydrophilic interactions. The dynamism of the GM1 oligosaccharide head allows it to adopt different conformations and thereby to interact through hydrogen or ionic bonds with a wide range of membrane receptors and extracellular ligands[5] and improve their membranal insertion most particularly into the lipid rafts. Although gangliosides in humans and mammals are generally detectable in most tissue types, their highest concentration is found in the *substantia grisea* of the brain. As part of the glycocalyx, gangliosides are particularly involved in essential functions such as cell differentiation and signal transduction. Because of its pleiotropic effect, GM1 has even been referred to, in the scientific literature, as the factotum of nature[6].

3.1.3 GM1 and PD:

At the molecular level, GM1 has shown several positive effects that could be relevant for PD. It allows the stabilization of several kinase receptors in the lipid rafts improving survival pathways activation

(e.g. neurotrophic tyrosine kinase receptors 1&2, nerve growth factor [NGF])[7,8]. GM1 with its amphiphilic property could reduce α-synuclein aggregation preventing its deleterious effect[9]. Finally, it was also suggested that GM1 can reduce inflammation[10]. Based on these effects, GM1 has been considered as a neuro-protective agent and its potential for the treatment of several neurological diseases has been investigated. A considerable amount of data is available regarding GM1 and neurological diseases. Most relevant studies are summarized in section **3.4.1 Clinical data on GM1**. More specific to this protocol, PD seems to stand out as a disease in which GM1 could play a key role. Experimentally, it was shown that mice deficient of GM1 displayed a phenotype compatible with PD that could be reversed by GM1 tretament[11,12]. Furthermore, PD patients present a reduced level of GM1 in the brain[13].

3.1.4 Therapy background

Currently, the main treatment of PD is directed towards compensating for the loss of dopaminergic neurons with levodopa[14] that is later metabolized to dopamine. Recent progress has been made with non-medical approaches, e.g., deep brain stimulation using electrodes placed in the brain to modify neuronal activity of the basal ganglia, thereby improving some signs of PD. All so far available treatments in PD do improve the condition of the patients, but to date no treatment has been found to modify the progression of the disease. Furthermore, many late motor and non-motor symptoms remain unaffected by the treatments, and patients are confronted with side effects of existing treatments. To this day, there continues to be a large unmet medical need in PD.
This trial is aiming at testing a new IMP for PD patients with an improved benefit/risk profile that aims at protecting neurons that would be impaired by the disease and therewith retaining their functionality. Thus, progression of the disease could be curbed and patients' overall condition and quality of life improved.

3.2 Investigational Product and Indication

InnoMedica is a company specialized in developing innovative drugs using liposomal formulations of known active compounds. GM1 has shown to be efficacious in PD patients (see section **3.4 Clinical Evidences to Date**) and we therefore propose the use of a liposomal formulation of GM1 for the treatment of PD.
Talineuren is a liposomal formulation of the API GM1 ganglioside at 6 mg/ml. The two other lipids used to form the liposome are sphingomyelin and cholesterol. Both of these excipients are metabolites like GM1 and have been previously used in other marketed liposomal drugs. Indeed, the anti-cancer drug Marqibo[redacted] uses a very similar lipidic excipient formula to build its liposomal "shell".
TLN appears as a translucent white dispersion and can be administered orally or by injection. Talineuren is supplied in 30 ml glass vials (180 mg of GM1 in phosphate buffer saline at pH = 6.8). Please refer to the IB for more details.
Liposomes in general provide a longer systemic circulation of the API making it more bioavailable for the targeted tissues. Moreover, it is expected to reduce the risk of an immunogenic response since the API is integrated in a lipid droplet. This potential elongated circulation time allows the increase of administration intervals, meaning avoidance of high frequency injections (e.g., daily s.c. as previously done in patients with free drug GM1). This might result, in the end, in improved cost efficiency (secondary added benefit of liposome). Finally, GM1 is naturally found in lipid rafts (specialized membrane microdomains in cells specialized in cell-cell communication) where its action might be more beneficial for the neuron's survival. Embedded in sphingomyelin and cholesterol that are also lipid raft constituents, GM1 in TLN is provided its natural raft-like configuration the way it appears in nature.

3.3 Preclinical Evidence

GM1 efficacy and safety have been well documented in human clinical trials. The pre-clinical studies aimed at evaluating the efficacy and safety of TLN in animals. Talineuren has been evaluated in several preclinical studies by the sponsor: on one hand, for efficacy in two mice models [redacted], on the other hand, for pharmacokinetics/biodistribution (PK/BD). Furthermore, another PK/BD study was performed in rats.

3.3.1 Preclinical efficacy

Therapeutic efficacy of TLN has been first investigated *in vivo* in the MPTP mouse model of PD. The neurotoxin 1-methyl-4-phenyl-1,2,3,6-tetrahydropyridine (MPTP) "attacks" the *substantia nigra* and results in reduced level of dopamine and its metabolites 3,4-dihydroxyphenylacetic acid (DOPAC) and homovanillic acid (HVA) in the striatum of the brain after repeated i.p. injections. This model is widely

used in preclinical research to evaluate the efficacy of new drugs in the context of PD. Several studies were run and are summarized in the IB. One particular study (named CR1 in the IB), where TLN was administered daily for 14 days (7.5 and 15 mg/kg TLN p.o.) showed partial rescue of the dopamine, DOPAC and HVA (particularly at 15 mg/kg p.o.) levels in the striatum. While modest, this effect was superior to the effect obtained when mice were treated with an equivalent dose of free GM1 i.p. Furthermore, there was a tendency of increased tyrosine hydroxylase-positive, i.e., dopamine producing cells in the *substantia nigra* in the TLN (15 and 30 mg/kg TLN p.o.) treated groups compared to controls and free GM1. These data showed a clear effect of TLN that was superior to that of the free drug.

[redacted]

3.3.2 Preclinical biodistribution

Several BD studies have been performed using TLN (listed and detailed in the IB). Optical imaging studies in mice (named VOX1-3 in the IB) with fluorescently labelled liposomes revealed that TLN's shell/carrier delivered the fluorescent label to the brain within 5 min after intravenous administration and remained at the same levels up to 48 h. In the four organs analysed *ex vivo*, the strongest fluorescent signal was found in the liver, followed by the spleen, spinal cord and brain.

[redacted]

3.3.3 Pharmacokinetics

The PK study in rats showed that TLN (daily injection of 13.6 mg/kg over 4 days) administered i.v. showed increased plasma levels of GM1 at 4 and 8 h after drug administration, that was cleared after 24 h and did not show accumulation over the 4 consecutive repeated administrations. TLN i.v. produced an Area Under the Concentration Time Curve (AUC) 2.5-fold higher than free drug GM1 administered i.v. in comparison. At the end of the experiment, total-GM1 levels in the brain were about two-fold higher when it was delivered in the form of TLN compared to when delivered as non-liposomal GM1 (free-drug). Altogether, these data indicate that TLN prolongs the bioavailability of GM1 and increases the delivery to the brain.

3.3.4 Toxicology

No apparent signs of toxicity were found in any of the *in vivo* studies that have been conducted with TLN. All treated animals (mice, rats) gained bodyweight comparable to untreated controls and appeared well and healthy at all times during the treatment (including chronic treatment up to 50 days, UBE2). As data are available for the non-liposomal API used in TLN (GM1), no GLP-compliant preclinical toxicity study has been performed with TLN. The supplier of TLN's GM1 conducted a number of GLP-compliant chronic toxicity studies with GM1 in different species, including a 4-week repeat i.v.-dose toxicity study in CD-rats with 14-day recovery period (n=80). In the latter study no adverse events (AE) were reported in the group receiving 10 mg/kg/day. Higher doses resulted in mild (55 mg/kg/day) and moderate (300 mg/kg/day) AE, mostly evident in histopathological findings and blood and chemistry lab value changes. Findings of these studies can be bridged to TLN, as in both cases the API is the GM1 molecule.
In conclusion, all the pre-clinical studies so far show that:

1- TLN is able to deliver GM1 (or other payload like the fluorescent dyes) to the targeted organ (brain or moto-neurons) as demonstrated by the biodistribution/efficacy studies, even if the liver is still clearing a large amount of TLN.
2- TLN allows longer circulation of GM1 in blood (as expected from liposomal formulation)
3- TLN does not induce obvious toxicity in animals

The limitations are the comparability of mice versus human in the context of neurodegenerative studies. Pharmacokinetics are hard to extrapolate from mice, furthermore all read-out tested so far are focused on the disease markers/features and may not evidence subtle AE or human specific issues.

3.4 Clinical Evidence to Date

To date there are no clinical data on TLN, but significant clinical data on the API (GM1) exist.

3.4.1 Clinical data on GM1

GM1 as monotherapy or as part of a mixture with other gangliosides has been investigated in various neurological indications in a large number of clinical and preclinical studies. 51 published clinical trials that used the API GM1 as a “free drug” were analysed and indicate good tolerability (complete list of publications in the IB). Moreover, there are numerous earlier clinical trials with GM1 which are not electronically available. Indications treated include ALS, PD, Alzheimer’s disease, peripheral neuropathies, cerebrovascular disease including cerebral infarction and subarachnoid haemorrhage, spinal cord injuries, sensory and motor neuronal degenerations, postherpetic neuralgia, tardive dyskinesia, and chorea. GM1 has also been studied in healthy volunteers.
Doses of GM1 administered range from 8.4 mg/day up to 2500 mg/day via varying routes of administration (i.v., i.m., s.c. or intraventricular) with treatment durations up to 1825 days (5 years[15]). In the above mentioned 51 trials 2658 patients were treated in total. The cumulative exposure amount calculated by number of patients multiplied by treatment days is 179’560 treatment days resulting in 491.9 patient-years. When restricting the criteria to intravenously administered pure GM1 (excluding ganglioside-mixtures and non-intravenous-routes of administration), literature search still yields a total of 24 clinical trials comprising nearly 37’000 treatment days and covering a dose spectrum of 80 to 2500 mg/day (see IB).
In the context of PD, we have singled out a few key publications that demonstrated the efficacy of GM1 that could be relevant for the present trial:

1- The neuroprotective effect seen *in vitro* and *in vivo* in a number of species was first reproduced in a clinical study published in 1998. In an open label clinical trial including 34 patients with PD, the UPDRS score was significantly improved[16]. In this trial the AE recording shows a similar profile among the GM1 treated patients and the controls.
2- A follow-up trial showed in a non-controlled way that the patients (N = 26) could be safely treated over a 5-year period of time and presented an overall significant decrease in their UPDRS score compared to the baseline, indicating an improvement of the functionality[15].
3- The same research group performed a larger randomized, blinded trial where they showed that progression of disease was almost completely halted over the total duration of the trial (2.5 years)[17]. The investigations were thereafter not followed up because the bi-daily s.c. injections caused local AEs (e.g., skin rash at the site of injection) disqualifying this means of administration for chronic long-term treatment.

In conclusion, GM1 has a strong potential for treating PD patients. With improved bioavailability and longer circulating time, TLN is expected to perform similarly or better than GM1 in PD patients.

3.4.2 Pharmacokinetics for GM1

Pharmacokinetics (PK) of GM1 has been studied in various set-ups. This is complicated by the fact that GM1 can be also synthetized/catabolised by the body. One study using radiolabelled GM1 showed that after i.v. injection, 90% of circulating GM1 is removed before 10 h and that it accumulates first in liver and second in the brain of rats[18]. In humans, the endogenous blood concentration is in the range of 100 ng/ml therefore not affecting measurements when 100, 200 or 300 mg where injected i.v. as a single bolus[19]. In this study, the PK analysis done by non-compartmental analysis showed a bi-phasic decrease, a relatively long mean resident time (43 h) and a very low clearance (below 3 ml/min). This means that GM1 is almost exclusively metabolised and either used in cells or further degraded. Finally, blood concentration of GM1 has been studied after i.m or s.c administration allowing a longer half-life in the blood which was postulated to obtain longer circulation time to increase brain delivery[20]. Indeed, when injected s.c. the delivery is delayed and the peak in blood reached after a couple of days. Interestingly, even if GM1 presents a rather long life in blood, the most successful studies used daily or even bi-daily GM1 injections to maintain high levels of GM1 in blood. This might be a pre-requisite for free GM1 to “pass” the blood brain barrier. PK of lipids cannot be analysed like small molecules or other active substances. In addition, liposomal formulations have a PK that is mainly driven by the liposome shell and less dependent from the API (inside). Clinical studies in humans are therefore necessary in order to understand the PK of TLN.

3.4.3 Clinical safety of GM1

The clinical tolerability profile of GM1 is extremely positive. So far, there are no indications of acute or long-term cumulative toxicity. Intravenous single doses of 2500 mg GM1 showed no acute clinical side effects. The repeated parenteral administration of high doses of GM1 (up to 2500 mg/day on 5 days per week each) resulted in increased cholesterol and triglyceride levels and an increase in

apolipoprotein B in healthy volunteers after 4 weeks. All changes were regredient after treatment was stopped. Apart from the above-mentioned laboratory readouts changes, no other effects were observed[21]. Over the total of the above mentioned 51 clinical studies the AEs reported are local pain during i.m. injections, rash and other skin reactions, exfoliative dermatitis, severe allergic reaction in one patient, hyperlipidaemia without a clear causal relationship to GM1. Furthermore, these AEs were only observed sporadically and inconsistently. Among all of the patients, one patient developed a symptom consistent with Guillain-Barré syndrome (GBS). This patient suffered a stroke before treatment with GM1 and had a long medical history including alcohol abuse, atrial fibrillation, congestive heart failure, angina, liver insufficiency, diabetes and chronic symmetric sensory neuropathy. The most reproductible AEs during long-term treatment of PD patients so far were found with 100 mg b.i.d. GM1 s.c. over a period of 2.5 to 5 years and were limited to local injection reactions such as redness and swelling[15,17].
The GBS is the only potential severe adverse effect discussed in the context of GM1. In the 1980s, ganglioside mixtures of bovine origin were used for the treatment of various neurological conditions in several European countries, especially in Italy and Spain. Following a number of GBS associated with ganglioside-mixture-treatments, the corresponding products were withdrawn from the European market in the early 1990s. Noteworthy, this withdrawal coincided in Europe with the peak time of bovine spongiform encephalopathy (BSE) through a newly discovered type of infection caused by prions, raising scrutiny for any human use of products derived from bovine brain. Meanwhile, bovine-free ganglioside products were developed from porcine brain material. BSE has never been documented in pigs and is almost eradicated in 2019, limiting the potential for a spread across species. Neither epidemiological studies[22,23] nor post-marketing safety data in over 1 million patients exposed to a GM1-product from porcine brain worldwide support the incidence of GBS to be associated with GM1 use. Additionally, the injection of GM1 alone had no immune-stimulating effects[24] and no anti-GM1 antibodies were detected after long-term treatment with GM1 doses of 1000 mg intravenous followed by 200 mg/day s.c. for 18 weeks[25]. GM1 derived from pigs, manufactured by TRB Chemedica, remains in clinical use as a mono-substance in several countries.

3.4.4 Clinical safety of TLN's excipients

Eventual excessive cholesterol or sphingomyelin enrichment are expected to be identified through collected lab values and should be reversible once the dose is lowered or treatment is suspended. In the routine lab analyses, liver enzymes will be monitored. Provided no enzymatic deficiencies like lipid storage disorders (e.g., gangliosidosis) are present, which would lead to a study exclusion in the first place, all molecules of TLN will enter the natural turnover of the respective molecular species. A 30 mL vial of TLN contains 180 mg of liposomal GM1[redacted]. The USFDA recommends a maximum dose of 300 mg of cholesterol daily, while no recommendation exists for sphingomyelin, daily dietary uptake of sphingolipids is evaluated at 400 mg. Use of TLN could then be compared to dietary uptake of these lipids. At the maximum planned dose of TLN, [redacted] will be injected, compared to weekly dietary uptake and/or recommended dose (2800 and 2100 mg respectively) both excipients remain in the range of a weekly dietary consumption and should not overly imbalance these two metabolites.

3.4.5 Products containing the API GM1 currently marketed worldwide

The manufacturer TRB Chemedica is authorized to provide GM1 under the name Sygen in Brazil, Sinaxial in Argentina and Shijieyin in China. The dose administration varies from 20 to 100 mg daily in i.m. or i.v. injections. The indications are initial or early stages of traumatic, acute vascular lesions of the central nervous system (brain and spinal cord), peripheral neuropathies and PD.

3.5 Dose Rationale

Four publications are used as reference points for the dose rationale of this trial.

1- Best efficacy results in PD patients were achieved by Schneider *et. al.* in 2013[17] in a controlled/randomized trial setting with a daily dose of 100 mg *b.i.d.* via s.c. injections over a period of 2.5 years.
2- The highest dose given to humans was in a trial published by Roberts *et. al.*[21] in 1993 where healthy volunteers received a daily dose of 2500 mg daily 5/7 days via i.v. injection for 6 weeks.
3- Treatment with GM1 via direct infusion into the brain was performed in patients suffering from Alzheimer's disease[26,27]. The daily dose was 20 to 30 mg of GM1 by intracerebroventricular infusion.

It was shown that 2x100 mg of free GM1 daily had a beneficial effect on the UPDRS of treated PD patients. The goal is to avoid daily-repeated injections because of the side effects they generate. In this trial the drug will be delivered i.v. as a weekly dose taking advantage of the longer circulation time of the liposomes. The known therapeutic dose, used in the study from Schneider *et. al*. in 2013, corresponds to a weekly exposure of 1400 mg of GM1. In China, for PD patients, the authorised dose is 80 mg daily (in addition to dopamine substitution therapy) resulting in 560 mg weekly GM1 administration. For TLN we expect a higher bioavailability due to the liposomal formulation. The starting dose is set to 6 mg TLN, which corresponds to 3% of the dose of GM1 that showed an effect when given s.c. and is less than 0.25% of the maximum that was ever applied i.v. at once (2500 mg). It is foreseen to increase the dose progressively to 12 and 60 mg then increasing by equal steps of 60 mg up to 720 mg. This planned maximum dose is 51% of the weekly exposure that was shown to be efficacious (s.c.) and 29% of the maximum dose injected at once (i.v.). Here a deliberately low dose was chosen to start with to ensure good tolerability at this level before incrementing the dose levels. At these doses and as a liposomal formulation we expect TLN to be efficacious, although the exposure is lower compared to that of the trial of Schneider *et. al.* in 2013. We moreover expect TLN to remain safe at these doses.
With the liposomal formulation of TLN there might be a more efficient blood brain barrier crossing and therefore more free-drug in the brain.
For the consolidation part, a 8 weeks treatment length has been selected since the UPDRS improvements have been described after 6 weeks in a previous study[17].

3.6 Explanation for choice of comparator (or placebo)

Not applicable

3.7 Risks / Benefits

According to the literature and non-clinical data of TLN, the profile of TLN is expected to be very safe. All components of TLN are metabolites that can be produced and broken down by the human body. There is very little risk of accumulation based on current knowledge. The known risks associated with GM1 from the literature are detailed in section **3.4.3 Clinical safety**. For the patients following the escalation schedule, the main risk is that TLN will be given for the first time in human as i.v. infusion. This is mitigated by a very low starting dose and a careful follow-up and escalation. The main risk potentially associated with TLN is an over performance compared with free GM1. Talineuren could reach the brain cells more efficiently than the free drug GM1 and might cause cerebral symptoms with disorientation, confusion, and headache similarly to what was observed in the trial using 50 mg intracranial injections on GM1[26]. With a liposomal formulation i.v. injected by continuous infusion that could be stopped if encephalopathic side effects appeared, sudden local increase of GM1 (e.g., in the brain) will be avoided and therefore this risk is considered minimal.
There are also risks inherent to the weekly i.v. injections and the local pain caused by this repeated procedure over 8 or 14 weeks.
The potential benefit is a disease-modifying treatment that may slow down or even halt the progression of the disease by reduction of the neuronal decay, which could preserve functioning neurons and prevent symptoms from appearing or worsening. GM1 has been shown to increase neuronal survival and reduce neuroinflammation. This has been observed and documented *in vitro* and *in vivo*. First beneficial effects (in terms of UPDRS score reduction, with 6 points improvement) may be noticeable as early as after 6 weeks of treatment[17]. The patients might clinically benefit from the drug even though this is only a phase I trial.
One potential risk identified is the potentially reduced need for levodopa in case that there was a significant therapeutic effect of TLN over the course of this trial. If the patients' condition improves the prescribed dose of levodopa might become too high. The levodopa treatment (or other similar medication) will then be continuously re-assessed during the trial by the PI and adjusted as clinically needed for the best care of the patient.
Although this is a safety trial and no potential benefits can be claimed to justify the risk for the patients, such a potential benefit does seem to be plausible (slowing of disease progression or even improvement) in this trial. However, the main benefit for the patients will be the participation in clinical research that may eventually improve the treatment of their condition.

3.8 Justification of choice of study population

In this phase I safety trial we wish to enrol PD patients only and do not plan the involvement of healthy volunteers.

The rationale for that is the following:

1- The API GM1 has been extensively used in humans over several years.
2- GM1 has already been administered to PD patients in other trials.
3- We expect a good safety profile, mainly because GM1 is a metabolite. The other compounds of TLN are also metabolites and should not present any safety issues.
4- The patients' current PD treatment (e.g., levodopa) will not be discontinued and therefore they will not miss out on any symptomatic treatment.
5- Lowered levels of GM1 have been shown in PD patients[13], therefore the risk of GM1 overdosing is lower in PD patients as compared to healthy volunteers.
6- TLN has not shown any sign of concern during extensive preclinical efficacy testing *in vivo*.
7- PD patients could potentially benefit significantly from the trial, hinting towards a better benefit/risk ratio in this population.

3.9 Justification for optional continued TLN infusions after the follow-up visit after the dose escalation part

If patients experience a benefit on their condition from the study treatment, and since TLN will not be readily available as a medication at the conclusion of the patients' planned participation in the trial, InnoMedica wishes to give the patients extended access to the IMP in a safe and controlled manner. Based on the patient's wish, the three patients from dose escalation part will have the option to continue further with weekly TLN infusions after their follow-up visit up for an additional 8 weeks. This will allow the continuation of an eventual benefit from TLN for the patients whilst gathering further safety data on the IMP from repeated dosing.

4. STUDY OBJECTIVES

4.1 Overall Objective

The overall objective of this trial is to define the safety profile of TLN, to find a recommended dose for a phase II trial and validate the schedule of TLN administration in the described patient population. The aim is to ensure that TLN can be repeatedly administered safely to patients, in a weekly dose by i.v. route at a dose that is expected to produce a medical improvement of the patient's condition. The dose will be gradually increased until the targeted dose reaches to the corresponding weekly exposure that previously showed a beneficial clinical response for the free-drug GM1. Preliminary efficacy will be assessed at these different doses in order to later decide which dose might be recommended for further phase II trials.

4.2 Primary Objective

The primary objective is to assess the safety and tolerability of TLN i.v. as weekly administration in patients receiving either escalating doses (up to 14) or multiple equal doses over a period of 8 weeks.

4.3 Secondary Objectives

The secondary objectives of this trial are to assess, if any disease improvement can be observed over the course of the administration of TLN. In addition, information on PK will be collected.

4.4 Safety Objectives

There are no additional safety objectives as the primary objective focuses on safety.

5. STUDY OUTCOMES

The outcomes focus on the safety profile of the IMP and a preliminary assessment of the clinical benefit of the drug.

5.1 Primary Outcomes

The primary outcome of this trial is safety, designed as the measurement of:

- Occurrence of AEs
- Occurrence of SAEs
- Occurrence of any routine lab test values out of normal range including anti-GM1 antibodies and apolipoprotein B.

5.2 Secondary Outcomes

The secondary outcomes are PD related and will consist in assessing the overall clinical performance during the course of the trial.
They will consist of the following measurements:

- Cognitive assessment (MoCA) to be assessed at baseline and the final assessment (1 week after the last TLN infusion). Two different versions of the MoCA will be used to limit test-retest-learning bias.
- Levodopa challenge test (LCT): Change in LCT between baseline assessment and the final assessment (1 week after the last TLN infusion).
- MDS-UPDRS: Evolution with time of the MDS-UPDRS performed at the time of each infusion (weekly), at the final assessment (1 week after the last TLN infusion) and during follow-up visit (4 weeks after the last TLN infusion).
- MDS-UPDRS-3 motor assessment (as part of the MDS-UPDRS) before and after the TLN infusion to assess if there is an immediate (symptomatic) effect of TLN on parkinsonian motor signs.
- PDQ-39: Difference in PDQ-39 summary index between baseline assessment and the final assessment (1 week after the last TLN infusion).
- ESS: Change in ESS between baseline assessment and the final assessment (1 week after the last TLN infusion).
- Mood/depression assessment by BDI. Measured at baseline and the final assessment (1 week after the last TLN infusion).
- Assessment of motivation/apathy with the SAS at baseline and the final assessment (1 week after the last TLN infusion).
- The Non-Motor Symptoms Questionnaire (NMSQuest) will be measured at baseline and weekly during the course of the trial.
- Levodopa equivalent daily dose (LEDD) will be calculated at each assessment visit.
- Pharmacokinetic (PK) of total GM1 in plasma over the first 96 h after the first infusion (only in part 2 of the trial).
- Determination of a maximum suitable dose (MSD).

5.3 Other Outcomes of Interest

Not applicable.

5.4 Safety Outcomes

Safety is the primary outcome of this trial (see section **5.1 Primary Outcome**).

6. STUDY DESIGN

6.1 General study design and justification of design

This trial is a phase I interventional trial consisting of a dose escalation part (1) and a dose consolidation part (2):

1- A single ascending dose part in 3 patients for up to 14 weeks plus a follow-up 4 weeks after the last infusion.
2- A repeated dose administration part in 9 patients for up to 8 weeks plus a follow-up 4 weeks after the last infusion.
3- Optional: The 3 patients form part 1, if they wish, could continue their treatment beyond the planned final assessments for up to 8 weekly TLN infusions.

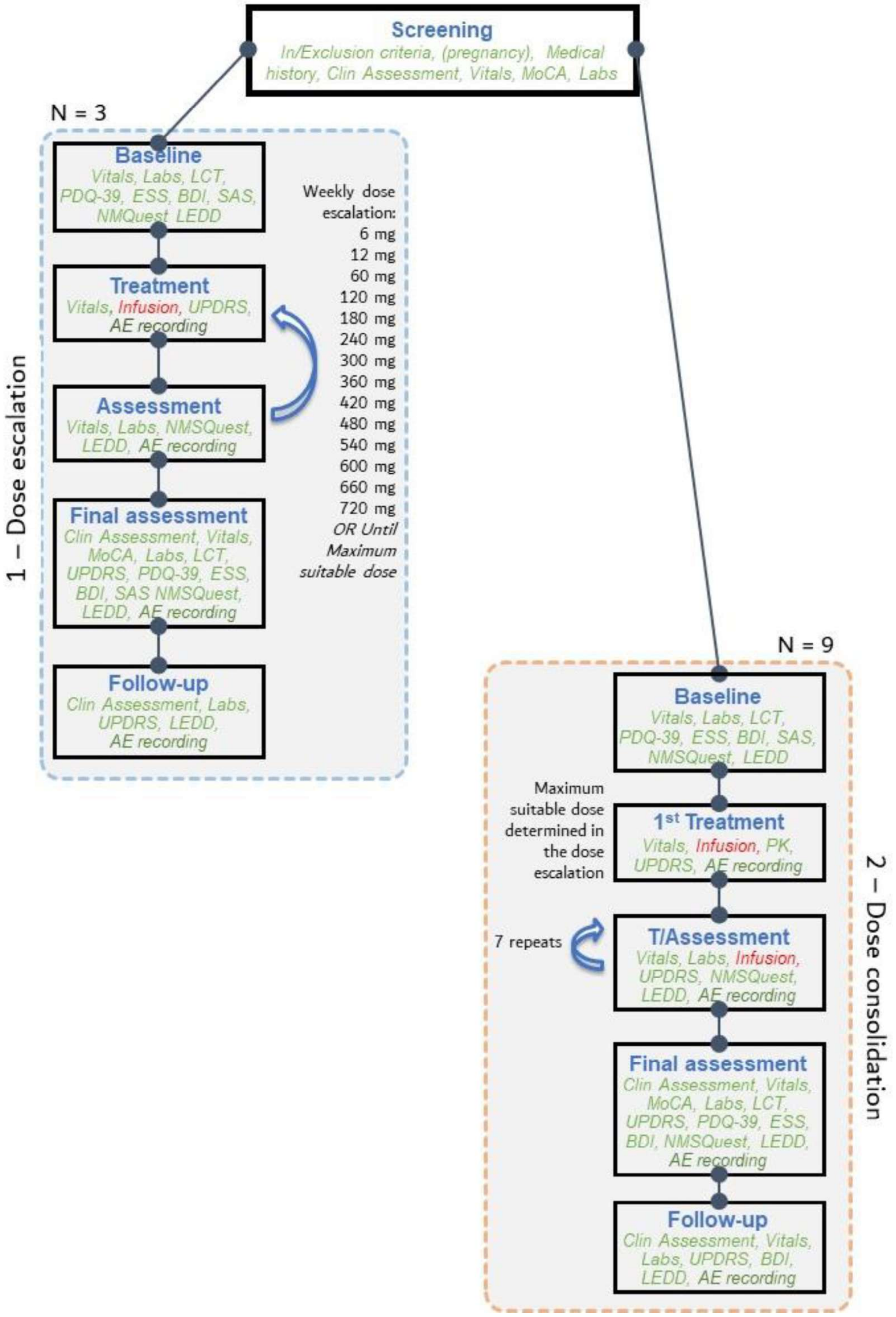

All data will be analysed in a descriptive manner in order to collect first clinical data with this new liposomal formulation of GM1.

6.1.1 Dose escalation (Part 1)

In this part a group of 3 patients will receive a single weekly dose that will be increased according to the schedule defined. In parallel, meaning that the first patient will receive a dose X on week Y, if the safety assessment is positive (see **Section 8.3 Dose modifications**) then the following week (Y+1) this patient will receive the next dose (X+1), on that week (Y+1) two other patients will receive the same dose X to confirm the safety assessment. *In fine*, every week patient 1 will receive one dose at the same time that patients 2 & 3 receive the preceding dose.
This weekly increase has been elected to allow rapid determination of the MSD and rapidly reach a dose level that will be beneficial for the patients involved in this trial. This is allowed by the fact that GM1 is expected to be safe and no clear AE are foreseen within this dose range. Furthermore, the 1 + 2 pattern allows one week observation of each dose increase in 1 patient before infusing 2 more.
The first dose level starts at 6 mg which will increase to 12 and 60 mg, then increasing by equal steps of 60 mg up to 720 mg. The escalation will be stopped individually according to the decision matrix detailed in the **Section 8.3 Doses modifications.** The three first steps of the dose are purposely low to account for any possible unexpected effects of the formulation.
Throughout the escalation part, all patients will be monitored twice weekly to record AEs and document them. Monitoring will take place once before each TLN infusion (beginning of the week) and a second time by the end of each week (performed by the principal investigator) to collect AE and allow enough time for the IMC to review them and take their decision on the next infusion/dose modification/delay.
At the end of the escalation, the average of the three maximum tolerated doses by the three patients will be defined as MSD for part 2. With this design, we aim to find the highest dose that generates, if any at all, at maximum only transient mild/moderate AEs. At the same time, this will allow to glean information on clinical effect for the planning of a potential phase II study.
If a patient drops out for reasons unrelated to the drug treatment, this patient will be replaced. The three MSD are necessary to establish the dose that will be used for the multiple dose administration. This is a design that allows fast escalation with minimal risks for the patients since the dose steps are small, the starting dose is very low and each increment is split in a 1 + 2 pattern. With N=3 we expect to show evidence of common AEs inherent to the TLN formulation. This design (part 1) lacks the assessment of mid-term cumulative effects of the drug at a constant dosage. Therefore, an additional phase (part 2) will be performed to complete the safety profile under constant weekly dosages and provide more insight of the tolerability over 8 repeated doses.

6.1.2 Dose consolidation (Part 2)

The MSD will then be administered repeatedly (8 times over 8 weeks) to 9 additional patients to consolidate what will be found in the escalation part.
At the first infusion, blood will be recovered for PK analysis over 96 h (see Section **9.2.2 Assessment of Secondary Outcomes**). The expected half-life is projected to be 30 h (extrapolation from pre-clinical studies). Therefore, 96 h would suffice to obtain 3 half-life periods and to observe enough tapering.
Throughout this part 2, all patients will be monitored weekly to record AE and document them. The clinical and PD assessment will be performed similarly to the dose escalation. This part will provide more information on the tolerability of the chosen MSD over repeated injections. This will complete the safety profile while avoiding to mobilize the whole cohort of patients for the dose escalation. This is still a modest number of patients but this will suffice to evidence the most common AEs associated with the treatment. Moreover, potential clinical effects on signs and symptoms of PD can be studied in view of the planning of a phase II study.
In this multiple dose administration part, patients that drop out for reasons unrelated to the treatment with TLN will be replaced due to the small number of patients involved.

6.1.3 Continued TLN infusions

Patients wishing to continue their treatment beyond the assessments in part 1 of the trial described above, can receive further weekly TLN infusions for up to 8 additional weeks at their personal MSD or at the dose level 14. A lighter schedule (one instead of two visits per week) than part 1 was elected for the patients' convenience. All patients will be monitored weekly to record AE and assessed based on details given in chapter **9. ASSESSMENTS**.

6.2 Methods of minimising bias

6.2.1 Randomisation

Not applicable.

6.2.2 Blinding procedures

Not applicable.

6.2.3 Other methods of minimising bias

For the primary endpoint, the assessments of the outcome are based on standards and norms. In this trial, the following validated instruments will be used to assess secondary endpoints: MDS-UPDRS, PDQ-39, ESS, NMSQuest, SAS, BDI, and MoCA.

7. STUDY POPULATION

7.1 Eligibility criteria

Participants fulfilling all of the following inclusion criteria are eligible for the trial:

1. Informed consent as documented by signature.
2. Male and female subjects, aged 40 to 80 years.
3. Confirmed Parkinson's disease according to British brain bank criteria[28].
4. Hoehn and Yahr Stage 0 – 2.5 on medication.
5. Stable PD treatment for 4 weeks at least.
6. Absence of dementia confirmed by cognitive testing (MoCA >25).

The presence of any one of the following exclusion criteria will lead to exclusion of the participant:

1. Contraindications to the class of drugs under study, e.g., known hypersensitivity or allergy to class of drugs or the investigational product.
2. Women who are pregnant or breast feeding, or planning to become pregnant during the course of the trial or in the 12 weeks following the trial.
3. Lack of safe contraception, defined as:
   - Female participants of childbearing potential, not willing to use double method of contraception (hormonal and mechanical) for the entire study duration. Female participants who are surgically sterilised / hysterectomised or post-menopausal for longer than 2 years are not considered as being of child bearing potential.
   - Male participants, not using and not willing to using a medically reliable method of contraception for the entire study duration, such as condoms or who are not using any other method considered sufficiently reliable by the investigator in individual cases.
4. Other clinically significant concomitant disease states (e.g., renal failure, hepatic dysfunction, cardiovascular disease etc.) that are not under stable control.
5. Known or suspected non-compliance, drug or alcohol abuse.
6. Inability to follow the procedures of the trial, e.g., due to language problems, psychological disorders etc. of the participant.
7. Participation in another trial with an investigational drug 4 weeks prior to the present trial.
8. Previous enrolment into the current trial.
9. Enrolment of the investigator, his/her family members, employees and other dependent persons.
10. Subject has an atypical parkinsonian syndrome or secondary parkinsonism.
11. Patients with comorbidity that may interfere with the course of the trial.
12. Patients who are not considered to be eligible to participate in clinical trial by the investigator.

7.2 Recruitment and screening

The patients will be identified by the PI according to the inclusion/exclusion criteria. If a patient is willing to participate in the trial, the informed consent procedure will thereafter be performed. Patients will be informed orally and in writing, that pre-screening assessments need to take place before enrolment into the trial. If patients do not meet these pre-screening criteria, they will be judged as screening failures. Patients will have up to 28 days of time to take their decision on whether to participate in the trial.

7.3 Criteria for withdrawal / discontinuation of participants

Patients will be withdrawn from the trial for any of the below listed reasons:

- Unacceptable toxicity as described in section **8.3 Dose modifications**
- Protocol treatment has to be delayed for more than 3 weeks
- Patient refuses further treatment
- Patient becomes pregnant
- Medical judgement by the treating physician
- Severe non-compliance with the trial protocol

For data and follow-up, see section **8.5 Data Collection and Follow-up for withdrawn participants.**

8. STUDY INTERVENTION

8.1 Identity of Investigational Products and administration

8.1.1 Experimental Intervention treatment

Talineuren is a liposomal formulation of GM1 ganglioside. The liposome acts as carrier for GM1 and is composed of cholesterol and sphingomyelin. For further information on the formulation of the IMP, please consult the current IB.
The experimental treatment with TLN consist of two parts:

Part 1 – Dose Escalation:
The drug will be administrated in a single ascending dose (SAD) as a once weekly i.v. infusion in 3 patients up to 14 dose levels or until unacceptable toxicity appears (see section **6.1.1 Dose escalation** for details on the sequence between patient 1 and 2/3).
The aim of this dose escalating procedure is to define the MSD in terms of the occurrence of AEs dependent on their seriousness, intensity and causality according to the standards and definitions given in this trial protocol (see section **8.3 Doses modifications**).

Dose level 1	6 mg
Dose level 2	12 mg
Dose level 3	60 mg
Dose level 4	120 mg
Dose level 5	180 mg
Dose level 6	240 mg
Dose level 7	300 mg
Dose level 8	360 mg
Dose level 9	420 mg
Dose level 10	480 mg
Dose level 11	540 mg
Dose level 12	600 mg
Dose level 13	660 mg
Dose level 14	720 mg

Part 2 – Dose consolidation:
As soon as the MSD is defined, 9 patients will receive the full MSD in the multiple dose administration part once a week for 8 weeks.

8.1.2 Control Intervention

Not applicable

8.1.3 Packaging, Labelling and Supply (re-supply)

Talineuren will be supplied as a white translucent solution prefilled in labelled glass vials with rubber stoppers. Each vial is packaged in a labelled cardboard box.
Talineuren will be provided free of charge by the sponsor and manufacturer InnoMedica AG Schweiz. Distribution to the site will be handled directly by InnoMedica Schweiz AG. An initial stock will be provided upon site activation. Thereafter, the site will be responsible for ordering additional TLN. The completed IMP order form (provided in ISF during initiation) should be sent to [redacted] no later than one working week prior to desired date of receipt.
Talineuren will be shipped temperature-controlled at temperatures between 2-8°C. For further details, please see the information on drug supply, handling and IMP deviations guideline.
Talineuren labels contain information to meet the applicable regulatory requirements. The investigational products will be labelled and handled as open-label material. Label sample is provided here below:

8.1.4 Storage Conditions

8.1.4.1 *Storage before dilution*

Talineuren is a sterile solution for i.v. infusion. The vials are for single use and must not be stored once opened. Talineuren must be stored at temperatures between 2-8°C. Storage area temperature conditions must be monitored and recorded.
Keep vial in cardbox until dilution.
In case the vial or the rubber stopper has been damaged, or any visible particles or sediments are in the vial, put the IMP in quarantine, complete a product quality complaint form and notify InnoMedica [redacted] immediately. For further details, please see the information on drug supply, handling and IMP deviations guideline.
Do not use vials after the retest date printed on each vial.

8.1.4.2 *Storage after dilution*

Diluted solution should be used immediately. If not used immediately, the solution may be stored in a refrigerator at 2-8°C for up to 24 h.
All temperature excursions outside of 2-8°C have to be documented. To report temperature deviations (during shipment and storage at the site), the product quality complaint form has to be completed by the site and forwarded to [redacted] immediately. The IMP needs to be put into quarantine until notification by the sponsor. For further details, please see the information on drug supply, handling and IMP deviations guideline.

8.2 Administration of experimental and control interventions

8.2.1 Experimental Intervention

Talineuren will be dispensed to the patient by health care professionals at the defined time points according to protocol. Blank spaces of the label (on box and vial) need to be filled in before the medication is dispensed to the patient. The experimental treatment consists of TLN and it will be administered once weekly as an intravenous infusion up to 14 weeks or until unacceptable toxicity (part 1) and up to 8 weeks or until unacceptable toxicity (part 2) or other reasons listed under section **8.3 Dose modifications**.

8.2.1.1 *Dilution*

Talineuren must never be administered undiluted. Before administration, TLN must be diluted with saline solution 0.9%. All planned doses are diluted by mixing the required dose of TLN (60 mg per 10 ml) in a 500 ml sterile bag pre-loaded with 250 ml of 0.9% saline solution.

8.2.1.2 *Administration*

After dilution, TLN is administered as intravenous infusion. In case of extravasation, infusion must immediately be stopped and restarted using a different vein. It is recommended to apply cold packs to

mitigate local irritation. In case of skin contact due to spilling or efflux of TLN solution from the infusion insertion site, rinse with water.
Administration speed is 250 ml/h using an infusion pump. During infusion, the solution should be protected from direct sunlight.

8.2.2 Control Intervention Experimental Intervention

Not applicable.

8.3 Dose modifications

Dose modifications are foreseen in cases where a causal relationship to the treatment with TLN cannot be excluded. A causal relationship in this trial is defined as a possible, probable or definite linkage to TLN according to ICH E2A (for more details see section **10. Safety**).
Any grade 4 event with causal relationship to treatment is judged as unacceptable toxicity and will lead to a stop of treatment without resuming the treatment in the affected patient.
Any SAE leads to a treatment stop for the affected patient with the following exception:
- if there is no causal relationship to TLN and the definition for an SAE is fulfilled **uniquely due to an overnight hospital stay (1 night) for an otherwise not severe or serious medical problem**, the patient can continue study participation.

In case of an SAE with no causal relationship to TLN, the patient will be replaced.

Part 1 – Dose escalation:

Causality	Intensity	Resolution until next infusion	Next patient added/ Dose increase
(unlikely/unrelated)	grade 1 - 3	yes/no	yes
Possible	grade 1	yes	yes
Possible	grade 1	no	yes
Possible	grade 2	yes	yes
Possible	grade 2	no	Delay till resolution (maximum of 3 weeks), then rechallenge at same dose level, if re-occurrence stop treatment for the affected patient
Possible	grade 3	yes/no	no (stop treatment for the affected patient but others to continue)
Probable/definite	grade 1	yes/no	yes
Probable/definite	grade 2	yes	yes
Probable/definite	grade 2	no	no (stop treatment for the affected patient but others to continue)
Probable/definite	grade 3	yes	stop (for the affected patient but others to continue)
Probable/definite	grade 3	no	Stop for all patients

Part 2 – Dose consolidation:

Causality	Intensity	Resolution until next infusion	Dose repeat
(unlikely/unrelated)	grade 1 - 3	yes/no	yes
Possible	grade 1	yes	yes
Possible	grade 1	no	yes
Possible	grade 2	yes	yes
Possible	grade 2	no	Delay till resolution (maximum of 3 weeks), then rechallenge at same level, if re-occurrence stop treatment for the affected patient

Possible	grade 3	yes/no	no (stop treatment for the affected patient but others to continue)
Probable/definite	grade 1	yes/no	yes
Probable/definite	grade 2	yes	yes
Probable/definite	grade 2	no	stop (for the affected patient but others to continue)
Probable/definite	grade 3	yes	stop (for the affected patient but others to continue)
Probable/definite	grade 3	no	stop (for the affected patient but others to continue)

Upon the PIs discretion and dependent on the general state of the patient standard PD medication can be changed during the course of the trial.

8.4 Compliance with study intervention

Patients will be treated in a clinical practice, in which the investigator can closely follow each patient. Further, the compliance will be assured by medically controlled administration of the IMP.

8.5 Data Collection and Follow-up for withdrawn participants

If a patient wishes to withdraw his/her consent and prefers to stop his/her participation in this trial (and for all patients with premature withdrawal) she/he will be proposed to undergo both a final assessment (7 days after the last TLN infusion) and follow-up visit (28 days after the last TLN infusion) according to the protocol.
In case patients withdraw their consent, all data collected until the time point of their withdrawal will be kept coded and may be analysed.

8.6 Trial specific preventive measures

As TLN is expected to have a good safety profile, no trial-specific preventive measures are taken in addition to the usual clinical preventive measures that are already in place.
Patients will receive an emergency contact card (see below) with details on their participation in this trial. Moreover, their general practitioner will receive information from the PI in order to be informed about their patients' participation in this trial. Both measures help creating preventive transparency. In addition, patients will receive a phone number (provided with the patient information and informed consent form, too), where they can reach a member of the trial team reachable 24 h/24 h.
In case of an unexpected medical emergency during a trial visit the patient would be transferred to a nearby located hospital equipped with an emergency unit.

8.7 Concomitant Interventions (treatments)

Patients are asked to continue their usual medications (including anti-parkinsonian medications). The participation in the trial is however not compatible with any other IMP. All medications used to treat PD will be documented in the patient's trial file.

8.8 Study Drug Accountability

The PI or a designee is responsible for drug accountability and record maintenance of the drug accountability log (including date, amount, batch number, expiry date and unique patient number (UPN)) for receipt, use, expiry and destruction of study drug. The drug accountability records must be kept up to date and must be available for monitoring purpose.

8.9 Return or Destruction of Study Drug

Unused or expired medication can only be destroyed at the site after the check of the monitor. Partly unused medication can be destroyed without the check of the monitor. Drug destruction has to be documented on the drug accountability log.

9. ASSESSMENTS

9.1 Table of trial procedures and assessments

Trial Periods	Screening	Baseline	Treatment (T)	Assessment (A)	T	A	T	A	T	A	T
Visit	1	2	3	4	5	6	7	8	9	10	11
Time (weeks)	-4 to -1	1	1	1	2	3	3	4	4	5	5
Time (days)	-28 to -1	0	1	4	8	11	15	18	22	25	29
Patient information & consent	x										
In-/Exclusion Criteria	x										
Pregnancy Test	x										
Medical History	x										
Clinical Assessment	x										
Vital Signs	x	x	x	x	x	x	x	x	x	x	x
MoCA	x										
Lab tests	x	x		x		x		x		x	
Levodopa Challenge Test		x									
TLN Infusion			6		12		60		120		180
MDS-UPDRS			x		x		x		x		x
PDQ-39		x									
ESS		x									
BDI		x									
SAS		x									
NMSQuest		x		x		x		x		x	
LEDD		x		x		x		x		x	
Adverse events recording			x	x	x	x	x	x	x	x	x

Trial Periods	A	T	A	T	A	T	A	T	A	T
Visit	12	13	14	15	16	17	18	19	20	21
Time (weeks)	6	6	7	7	8	8	9	9	10	10
Time (days)	32	36	39	43	45	49	52	56	59	63
Patient information & consent										
In-/Exclusion Criteria										
Pregnancy Test										
Medical History										
Clinical Assessment										
Vital Signs	x	x	x	x	x	x	x	x	x	x
MoCA										
Lab tests	x		x		x		x		x	
Levodopa Challenge Test										
TLN Infusion		240		300		360		420		480
MDS-UPDRS		x		x		x		x		x
PDQ-39										
ESS										
BDI										
SAS										
NMSQuest	x		x		x		x		x	
LEDD	x		x		x		x		x	
Adverse events recording	x	x	x	x	x	x	x	x	x	x

Trial Periods	A	T	A	T	A	T	A	T	Final assessment	Follow-up
Visit	22	23	24	25	26	27	28	29	30	31
Time (weeks)	11	11	12	12	13	13	14	14	+1	+4
Time (days)	66	70	73	77	80	84	87	91	+7	+28
Patient information & consent										
In-/Exclusion Criteria										
Pregnancy Test										
Medical History										
Clinical Assessment									x	x
Vital Signs	x	x	x	x	x	x	x	x	x	x
MoCA									x	
Lab tests	x		x		x		x		x	x
Levodopa Challenge Test									x	
TLN Infusion		540		600		660		720		
MDS-UPDRS		x		x		x		x	x	x
PDQ-39									x	
ESS									x	
BDI									x	
SAS									x	
NMSQuest	x		x		x		x		x	
LEDD	x		x		x		x		x	x
Adverse events recording	x	x	x	x	x	x	x	x	x	x

Schedule of assessment part 1:

Patients foreseen for part 1 of the trial, will ideally start their trial enrolment and treatment concomitantly. Before the first TLN infusion, patients will be screened for their participation in the trial. If all eligibility criteria are fulfilled, each of the 3 patients will start with an infusion containing 6 mg TLN (day 0). On day 4 they will be assessed according to the description given in chapter 9.3. Procedures at each visit. Only if the criteria as defined in chapter 8.3 Dose modifications are met, the patient can continue to the next dose level which he/she will receive on day 8. All patients will continue in the same manner and receive a weekly infusion of TLN until criteria are met to stop the treatment. Patients have to show up 3 times in week 1 and as of week 2 twice a week. All visits will take place at the Institute of Neurology in Konolfingen.

Trial Periods	Screening	Baseline	Treatment (T)	PK 24h	PK 48h	PK 72h	PK 96h	T	T	T	T	T	T	T	Final assessment	Follow-up
Visit	1	2	3	4	5	6	7	8	9	10	11	12	13	14	15	16
Time (weeks)	-4 to -1	1	1	1	1	1	1	2	3	4	5	6	7	8	+1	+4
Time (days)	-28 to -1	0	1	2	3	4	5	8	15	22	29	36	43	50	+7	+28
Patient information & consent	x															
In- /Exclusion Criteria	x															
Pregnancy Test	x															
Medical History	x															
Clinical Assessment	x														x	x
Vital Signs	x	x	x					x	x	x	x	x	x	x	x	x
MoCA	x														x	
Lab tests	x	x						x	x	x	x	x	x	x	x	x
Levodopa Challenge Test		x													x	
TLN Infusion			x					x	x	x	x	x	x	x		
Blood collection (PK)			x	x	x	x	x									
MDS-UPDRS			x					x	x	x	x	x	x	x	x	x
PDQ-39		x													x	
ESS		x													x	
BDI		x													x	
SAS		x													x	
NMSQuest		x						x	x	x	x	x	x	x	x	
LEDD		x						x	x	x	x	x	x	x	x	x
Adverse events recording			x	x	x	x	x	x	x	x	x	x	x	x	x	x

Schedule of assessment part 2:

In summary: Nine patients in total are foreseen to participate in part 2 of the trial. These 9 patients will all receive the MSD during 8 weeks. Between 28 days and one day before the start of the trial for them, patients will be screened if they qualify for the participation in the trial. If all eligibility criteria are fulfilled, patients will be enrolled to the trial and trial specific procedures will start. During week 1, patients have to show up every day at the CI Bern. CI Bern is a clinical trial unit providing facilities and services to support clinical trials and serves as satellite site. CI Bern was chosen in order to perform PK sampling and assessments. As of week 2, patients will only have one visit per week, where treatment (TLN infusion) and assessments will take place at the same time. These visits will take place in the Institute of Neurology in Konolfingen
All patients will continue in the same manner and receive a weekly infusion of TLN until criteria are met to stop the treatment.

Patients form the part 1 of the trial, wishing to take the option of continuing the treatment will follow the same schedule as the patients of part 2 (see schedule of assessment above) with the exception of the PK sampling, and baseline (the data will be added to part 1). All assessments will take place in the Institute of Neurology in Konolfingen.

Trial Periods	Re-consent	T	T	T	T	T	T	T	T	Final assessment	Follow-up
Visit	0	1	2	3	4	5	6	7	8	9	10
Time (weeks)	-4 to -1	1	2	3	4	5	6	7	8	+1	+4
Time (days)	-28 to 0	1	8	15	22	29	36	43	50	+7	+28
Patient information & consent	x*										
Clinical Assessment										x	x
Vital Signs		x	x	x	x	x	x	x	x	x	x
MoCA										x	
Lab tests		x	x	x	x	x	x	x	x	x	x
Levodopa Challenge Test										x	
TLN Infusion		x	x	x	x	x	x	x	x		
Blood collection (PK)											
MDS-UPDRS		x	x	x	x	x	x	x	x	x	x
PDQ-39										x	
ESS										x	
BDI										x	
SAS										x	
NMSQuest		x	x	x	x	x	x	x	x	x	
LEDD		x	x	x	x	x	x	x	x	x	x
Adverse events recording		x	x	x	x	x	x	x	x	x	x

Schedule of assessment prolongation (optional): *Patient consent for the treatment prolongation might also be collected at the final assessment or follow-up visit of the schedule of part 1.

9.2 Assessments of outcomes

9.2.1 Assessment of primary outcome

Clinical AEs, lab values and vital signs, will be assessed during each visit as reflected in the two schedules (part 1 and part 2 of the trial). In addition, patients will also be asked to contact the PI's practice if they experience any adverse effect between visits, to assess if this could be related to the treatment and would require further medical attention and if any actions are to be taken. Patients will be asked to provide as much detail as possible at the time of the visit. Details should include the time and duration of these occurrences that will be examined with the investigator to fill the weekly reporting of AE. The investigator will document the AEs in the eCRF after each visit and will notify the sponsor in case of SAEs. All AEs and SAEs will be documented as follows: time of onset, duration, resolution, action to be taken, assessment of intensity, relationship with trial treatment (for all details refer to section **10 SAFETY**). Pre-existing signs and symptoms, especially of PD, are not to be considered an AE. Parkinsonian signs and symptoms are considered an AE in this trial if they appear to be new or if a previously present non-fluctuating symptom clearly worsens. A fluctuating parkinsonian symptom will only be counted as an AE if the fluctuation clearly increases in frequency or duration or if the extent of the fluctuating symptom at its worst is clearly worse compared to the baseline condition.

In order to assess other safety issues, blood samples will be taken and analysed for routine assessments (termed full lab tests thereafter) to assess if TLN modifies the blood cell count, the electrolytes, metabolites and liver markers (for the full list of lab tests and standard values see the **Annexes 1 & 2**) or result in an elevation of anti-GM1 antibodies or apolipoprotein B (a marker of hyperlipidaemia).

The full lab tests consist of:

1- The "routine" lab analyses will be performed (Cat #: Rout-3549)
2- Anti-GM1 IgG antibodies (Cat #: gm1g 52250)
3- Apolipoprotein B (Cat #: apob-7320) assays

All required material will be provided by Viollier. 4 tubes (3 x 0.5 ml of blood serum-gel-tube and 1 x 0.5 ml of blood in EDTA tube) will be required for these tests from each patient.

Safety lab values will be assessed from blood collection performed every week during part 1 (3 days after TLN infusion) and part 2 (on the day of TLN infusion) according to the schedule. Abnormal

values will be compared to the normal range provided by the laboratory (**Annex 2**), then assessed by the PI for grading. All analyses will be performed from venous blood collection before the infusion. Samples will be collected on each sampling day by courier by Viollier and processed in their facilities.

9.2.2 Assessment of secondary outcomes

- Cognitive assessment: Montreal Cognitive Assessment (MoCA): The MoCA is a short screening test for the detection of cognitive impairment and dementia. The test will take approximatively 15 min in cognitively unimpaired participants.
- Levodopa challenge test (LCT): The LCT will be performed in a "defined off" because "worst off" cannot be obtained within reasonable time. All PD medications are stopped at least 12 h before the test. A motor assessment (MDS-UPDRS-3) is performed in this defined "off" state. Then the LED of the morning medication plus 50 mg of levodopa is given p.o. as soluble levodopa (Madopar liquid). The motor assessment is then repeated 1 h later. Usual daily medication is resumed after the LCT. In total the test lasts for maximally 2 h.
- MDS-UPDRS: The MDS-UPDRS consists of four parts: Part I (experiences of daily life - non-motor aspects), Part II (experiences of daily life - motor aspects), Part III (motor examination) and Part IV (motor complications). Part I consists of two components: Part Ia relates to various behavioural problems that the PI will evaluate using the information available from the patient and the caregiver (if available). Part Ib should be completed by the patient with or without the support of the caregiver, but independently of the PI. However, this part can be checked by the PI in order to ensure a clear and unambiguous answer to the questions. The investigator can also help to explain any uncertainties. Part II, like Part Ib, is designed as a self-questionnaire, but Part II can also be checked by the PI for completeness and freedom from errors. Part II will be assessed for best and worst condition in the previous week in patients with fluctuating parkinsonian signs and symptoms. Part III contains instructions for the PI, which are read to the patient or demonstrated directly; this part is completed by the PI as a motor examination. Part IV contains instructions for the investigator and also instructions that must be read to the patient. This part combines patient-related information with the clinical observations and assessments of the examiner and is therefore also filled out by the examiner. To investigate if the infusion has an immediate motor effect on the patient's condition, Part III will be repeated before and after the infusion. The questionnaires of the MDS-UPDRS (parts 1, 2 and 4) will take up to 1 h; for this study, these parts that refer to the previous week will be assessed in an interview with the patient to guarantee accurate responses.
- Parkinson's Disease Questionnaire 39 (PDQ-39): Difference in the PDQ-39 summary index between baseline assessment and the final assessment (1 week after the last TLN infusion). The PDQ-39 is a disease-specific questionnaire for quality of life of the affected patients. The 39 items are subdivided into 8 subscales: mobility, daily activity, emotional well-being, stigma, social support, cognition, communication, physical discomfort.
  Patients will be asked to complete the questionnaire by using the five categories for their answers that are foreseen with a coding from 0-4 (0 = «niemals»; 1 = «selten»; 2 = «manchmal»; 3 = «häufig»; 4 = «immer oder kann ich überhaupt nicht»). The completion of the questionnaire will take the patients 5 to 10 min.
- Epworth Sleepiness Scale ESS): The ESS is a questionnaire to help measure the general level of daytime sleepiness. Patients are to rate the chance that they would doze off or fall asleep during eight different routine daytime situations. Each item is rated from 0 to 3 (0 = "würde niemals einnicken"; 1 = "geringe Wahrscheinlichkeit einzunicken"; 2 = "mittlere Wahrscheinlichkeit einzunicken"; 3 = "hohe Wahrscheinlichkeit einzunicken"). The completion of the questionnaire will take the patients approximately 5 min.
- Assessment of mood using the Beck Depression Inventory (BDI): Patients will be asked to complete the 21 questions on how they felt during the last week. Four options for their answer are foreseen (e.g. "ich bin nicht traurig", "ich bin traurig", "ich bin die ganze Zeit traurig und komme nicht davon los», «ich bin so traurig oder unglücklich, dass ich es kaum noch ertrage»). The completion of the questionnaire will take the patients 5 to 10 min.
- Motivation assessment by the Starkstein Apathy Scale (SAS)[29]: Patients will be asked to complete the 14 questions concerning their interest and motivation for certain activities. Four options for their answer are foreseen ("überhaupt nicht", "ein wenig", "ziemlich viel", "sehr viel"). The completion of the questionnaire will take the patients 5 to 10 min.

- Non-Motor Symptoms Questionnaire (NMSQuest): This questionnaire enables clinicians to complete a comprehensive assessment of a diverse range of non-motor symptoms which can occur in all stages of PD. It is a patient-based screening tool designed to draw attention to the presence of non-motor symptoms in patients for further investigation or treatment. Patients will be asked to give their answer ("ja" or "nein") to 30 questions.
- Levodopa equivalent daily dose (LEDD): LEDD will be assessed at each visit and will be calculated according to Verber *et. al*[30].
- Pharmacokinetics (PK) of total GM1 in blood over the first 96 h after the first infusion (only in part 2 of the trial):
  The assessment of PK will be determined by the calculation of the Maximum Observed Drug Concentration (C_{max}) in plasma, the Time of Maximum Drug Concentration (T_{max}) in plasma, $AUC_{inf.}$ in plasma, half-life ($t_{1/2}$) Clearance (CL and the volume of distribution (Vd).
  The predicted half-life of the drug is 30 h according to our pre-clinical data obtained in animals. The blood collection for PK will therefore be performed at the following timepoints after the first infusion (only part 2 of the trial):
  - 0 min (right before the infusion, pre-dose)
  - 5 min before the end of the infusion
  - 30 min after infusion
  - 1 h after infusion
  - 4 h after infusion
  - 24 h after infusion
  - 48 h after infusion
  - 72 h after infusion
  - 96 h after infusion

  Instructions for the collection of blood and the processing and handling are provided in **Annex 3**).
- Determine the maximal suitable dose (MSD): MSD is calculated as the average dose from all maximally reached doses by the 3 patients in the dose escalating part of this trial.

9.2.3 Assessment of other outcomes of interest

Not applicable

9.2.4 Assessment of safety outcomes

Not applicable (described under section **9.2.1. Assessment of primary outcome**)

9.2.5 Assessments in participants who prematurely stop the study

See chapter **8.5 Data Collection and Follow-up for withdrawn participants**.

9.3 Procedures at each visit

All patients will be screened and enrolled by the PI (this applies for part 1 and part 2). All assessments will be done by qualified trial personnel as declared on the authorisation log. Visits can be performed +/- 1 d from the described timepoint with the exception of the first treatment visit, which can be performed +7 d from the baseline visit.

Dose escalation (part 1)

9.3.1 Visit 1: Screening (between day -28 until day -1)

All patients will be screened by the PI. Patients willing to sign the informed consent form will be informed orally and in writing in detail about the exact procedures, their rights and duties. They will especially be informed that some screening assessments need to be done in order to decide if eligibility criteria are met.

- Explanation of the trial (risk/benefit) to the patients (60-120 min). Patient information is handed out in writing.
- Checking of all in- and exclusion criteria (10 min)
- Evaluation of the patient's medical history (10 min)

If inclusion and exclusion criteria are met as far as can be ascertained so far, and if at least 24 h have passed since patient information was given, written informed consent is signed by the participant and the PI. Then the following baseline assessments are performed:

- Pregnancy test for female patients with childbearing potential (blood test hCG).

- Clinical assessment (including physical medical and neurological exam, height and weight).
- Vital signs
- MoCA (20 min)
- Full lab tests

If all inclusion and exclusion criteria are still met, the patient is included in the trial, otherwise a screening failure is declared.

9.3.2 Visit 2: Baseline (day 0)

- Vital signs
- Full lab tests
- LCT
- PDQ-39
- ESS
- BDI
- SAS
- NMSQuest
- LEDD

9.3.3 Visit 3, 5, 7, 9, 11 and every other visit until visit 29: Treatment

- Vital signs
- TLN Infusion
- MDS-UPDRS (Part III before the infusion; Part I, II and IV during the infusion; Part III again after the infusion)
- AE recording

9.3.4 Visit 4, 6, 8, 10, 12 and every other until visit 28: Assessment

- Vital signs
- Full lab test
- NMSQuest
- LEDD
- AE recording

9.3.5 Visit 30: Final assessment (7 days after last TLN infusion)

- Clinical assessment (including physical medical and neurological exam, height and weight).
- Vital signs
- MoCA
- Full lab test
- LCT
- MDS-UPDRS
- PDQ-39
- ESS
- BDI
- SAS
- NMSQuest
- LEDD
- AE recording

9.3.6 Visit 31: Follow-up (28 days after TLN infusion)

- Clinical assessment (including physical medical and neurological exam, height and weight).
- Vital signs
- Full lab test
- MDS-UPDRS
- LEDD
- AE recording

Dose consolidation (part 2)

9.3.7 Visit 1: Screening (between day -28 until day -1)

All patients will be screened by the PI. Patients willing to sign the informed consent form will be informed orally and in writing in detail about the exact procedures, their rights and duties. They will especially be informed that some screening assessments need to be done in order to decide if eligibility criteria are met.

- Explanation of the trial (risk/benefit) to the patients (60-120 min). Patient information is handed out in writing.
- Checking of all in- and exclusion criteria (10 min)
- Evaluation of the patient's medical history (10 min)

If inclusion and exclusion criteria are met as far as can be ascertained so far, and if at least 24 h have passed since patient information was given, written informed consent is signed by the participant and the PI. Then the following baseline assessments are performed:

- Pregnancy test for female patients with childbearing potential (blood test hCG).
- Clinical assessment (including physical medical and neurological exam, height and weight).
- Vital signs
- MoCA (20 min)
- Full lab tests

If all inclusion and exclusion criteria are still met, the patient is included in the trial, otherwise a screening failure is declared.

9.3.8 Visit 2: Baseline (day 0)

- Vital signs
- Full lab tests
- LCT
- PDQ-39
- ESS
- BDI
- SAS
- NMSQuest
- LEDD

9.3.9 Visit 3: Treatment (day 1 - this visit takes place at the CI Bern)

- Vital signs
- Full lab tests
- MSD Infusion of TLN
- Blood collections for PK analysis
- MDS-UPDRS (Part III before the infusion; Part I, II and IV during the infusion; Part III again after the infusion)
- AE recording

9.3.10 Visit 4 – 7: PK (days 2 to 5 - these visits take place at the CI Bern)

- Blood collections for PK analysis
- AE recording

9.3.11 Visit 8 – 14: Treatment (days 8, 15, 22, 29, 36, 43 & 50)

- Vital signs
- Full lab tests
- MSD infusion of TLN
- MDS-UPDRS (Part III before the infusion; Part I, II and IV during the infusion; Part III again after the infusion)
- NMSQuest
- LEDD
- AE recording

9.3.12 Visit 15: Final assessment (7 days after last TLN infusion)

- Clinical assessment (including physical medical and neurological exam, height and weight).
- Vital signs

- MoCA
- Full lab tests
- LCT
- MDS-UPDRS
- PDQ-39
- ESS
- BDI
- SAS
- NMSQuest
- LEDD
- AE recording

9.3.13 Visit 16: Follow-up (28 days after last TLN infusion)

- Clinical assessment (including physical medical and neurological exam, height and weight).
- Vital signs
- Full lab tests
- MDS-UPDRS
- LEDD
- AE recording

After dose escalation: optional further treatment

9.3.14 Visit 0: “Re-consent” (day 0)

- Explanation of the option of treatment prolongation (15 min).
  Patient information is handed out in writing. This procedure might also take place during the final assessment or follow-up visit of part 1. Then this visit will not take place.

9.3.15 Visit 1-8: Treatment (day 1, 8, 15, 22, 29, 36, 43 & 50)

- Vital signs
- Full lab tests
- MSD Infusion of TLN
- MDS-UPDRS (Part III before the infusion; Part I, II and IV during the infusion; Part III again after the infusion)
- NMSQuest
- LEDD
- AE recording

9.3.16 Visit 9: Final assessment (7 days after last TLN infusion)

- Clinical assessment (including physical medical and neurological exam, height and weight).
- Vital signs
- MoCA
- Full lab tests
- LCT
- MDS-UPDRS
- PDQ-39
- ESS
- BDI
- SAS
- NMSQuest
- LEDD
- AE recording

9.3.17 Visit 10: Follow-up (28 days after last TLN infusion)

- Clinical assessment (including physical medical and neurological exam, height and weight).
- Vital signs
- Full lab tests
- MDS-UPDRS
- LEDD
- AE recording

10. SAFETY (DRUG STUDY)

As sponsor of this trial, InnoMedica has established a framework of quality assurance which encompasses responsibilities and documents (e.g., SOPs) which fully cover safety aspects.
During the entire duration of the trial, all AEs and all serious adverse events (SAEs) are collected, fully investigated and documented in source documents and case report forms (CRFs). Trial duration encompassed the time from when the participant signs the informed consent until the last protocol-specific procedure has been completed, including a safety follow-up period of 28 days. The SMP provides detailed information.

10.1 Definition and assessment of (serious) adverse events and other safety related events

An **Adverse Event (AE)** is any untoward medical occurrence in a patient or a clinical investigation participant who was administered a pharmaceutical product and which does not necessarily have a causal relationship with the study procedure. An AE can therefore be any unfavourable and unintended sign (including an abnormal laboratory finding), symptom, or disease temporally associated with the use of a medicinal (investigational) product, whether or not related to the medicinal (investigational) product.
AEs (and SAEs) will be documented by the PI and collected and documented in the DB.

A **Serious Adverse Event (SAE)** is classified as any untoward medical occurrence that:

- results in death,
- is life-threatening,
- requires in-patient hospitalization or prolongation of existing hospitalisation,
- results in persistent or significant disability/incapacity, or
- is a congenital anomaly/birth defect.

In addition, important medical events that may not be immediately life-threatening or result in death, or require hospitalisation, but may jeopardise the patient or may require intervention to prevent one of the other outcomes listed above should also usually be considered serious.
SAEs should be followed until resolution or stabilisation. Participants with ongoing SAEs at study termination (including safety visit) will be further followed up until recovery or until stabilisation of the disease after termination.
In order to standardise all AEs, they will be coded according to the Medical Dictionary for Regulatory Activities (MedDRA). Adverse events and SAEs will be summarized by body system and preferred term based on the Medical Dictionary for Regulatory Activities (MedDRA).

A **Suspected Unexpected Serious Adverse Reaction (SUSAR)** will be assessed based on possible AEs.
As there are no data for TLN in humans available, the following list of possible AEs is based on literature from clinical trials in which GM1 was applied in different galenic forms and dosages. If any of these AEs would appear in the trial, they would be judged as “expected” AEs.

- Local pain
- Pruritus
- Vasovagal reaction
- Urinary tract infection
- Mild head soreness
- Nocturnal confusion
- Exfoliative dermatitis
- Mild meningitis
- Slightly increased CSF/serum albumin
- Increase in systolic blood pressure
- Hyperreflexia
- Transient rash
- Conjunctivitis
- Modest elevation in cholesterol and triglycerides
- Erythema
- Hyperthermia
- Shivering
- Confusional state

- Severe allergic reaction
- Polyradiculopathy
- GBS
- Haematuria
- Hyperlipidaemia (Cholesterol, triglycerides, Apolipoprotein B)
- Swelling nodule
- Bleeding/hematoma
- Urticaria
- Induration
- Asthenia
- Worsening of PD symptoms
- Anastomotic ulcer/stomach cancer
- Unsteadiness
- Overactivity

Assessment of Causality

Both Investigator and sponsor make a causality assessment of the event to TLN, based on the criteria listed in the ICH E2A guidelines:

Relationship	Description
Definitely	Temporal relationship<br>Improvement after dechallenge*<br>Recurrence after rechallenge<br>(or other proof of drug cause)
Probably	Temporal relationship<br>Improvement after dechallenge<br>No other cause evident
Possibly	Temporal relationship<br>Other cause possible
Unlikely	Any assessable reaction that does not fulfil the above conditions
Not related	Causal relationship can be ruled out
*Improvement after dechallenge only taken into consideration, if applicable to reaction	

Unexpected Adverse Drug Reaction (UADR)

An “unexpected” adverse drug reaction is an adverse reaction, the nature or intensity of which is not consistent with the list of AEs provided above in this chapter.

Suspected Unexpected Serious Adverse Reactions (SUSARs)

InnoMedica evaluates any SAE that will be reported regarding seriousness, causality and expectedness. If the event is related to the investigational product and is both serious and unexpected and not listed as possible AE, it will be classified as a SUSAR.

Assessment of Intensity

Grade	Type	Description
1	mild	Awareness of a sign or symptom that does not interfere with the trial participant’s usual activity or is transient, resolved without treatment and with no sequelae
2	moderate	Interferes with the trial participant’s usual activity and/or requires symptomatic treatment.
3	severe	Medically significant, but not life-threatening. Symptom(s) causing severe discomfort and significant impact of the trial participant’s usual activity and requires treatment.
4	Life-threatening	Emergency intervention needed.
5	lethal	Death due to SAE.

10.1.1 Reporting of SAE and other safety related events

Reporting of AEs
Any AE will be recorded by the PI in the patient's case report file during the assessment visits and if reported by the patient between visits. All AEs will be assessed by the IMC on a weekly basis afterwards.

Reporting of SAEs
All SAEs must be reported immediately and within a maximum of 24 h to the sponsor. The sponsor will re-evaluate all reported SAEs in regard to seriousness and causality and, in case of a causal relationship, is given, to expectedness, and inform the site in case of discrepancies to the initial assessment done by the PI.
SAEs resulting in death are reported by the sponsor to the CEC via BASEC within 7 days.

Reporting of SUSARs
If a SAE is categorised as SUSAR, it will be reported by the sponsor to the CEC via BASEC and to Swissmedic within 7 days if the event is fatal, or within 15 days (all other SUSARs).

Reporting of Safety Signals
All suspected new risks and relevant new aspects of known adverse reactions that require safety-related measures, i.e. so called safety signals, must be reported to the sponsor within 24 h. The sponsor will report the safety signals within 7 days to the CEC via BASEC and to Swissmedic.

Reporting and Handling of Pregnancies
Pregnant participants must immediately be withdrawn from the clinical trial. Any pregnancy during the treatment trial and within 28 days after discontinuation of TLN has to be reported to InnoMedica within 24 h. The course and outcome of the pregnancy will be followed up carefully. In case a female partner of a trial participant gets pregnant during the trial, she will be asked by the PI to give her consent in order to collect data regarding the outcome of the mother or the child. Any abnormal outcome regarding the mother or the child should be documented and reported.

Periodic reporting of safety
An ASR will be submitted once a year by the sponsor to the CEC and to Swissmedic.

10.1.2 Follow up of (S)AEs

Patients presenting AEs will be followed by the sponsor until the last visit of the patient.
If further support is needed, then the patient will be treated according to the PIs discretion according to clinical practice.

11. STATISTICAL METHODS

The present section outlines the statistical principles for the planned analyses of this phase I trial. The analysis will be exploratory and primarily employ descriptive statistical methods. Inferential methods will be used to highlight interesting aspects of the data. Unless otherwise specified statistical tests will be two sided and conducted at the 5% significance level. Corresponding 95%-confidence intervals will be presented and no correction for multiplicity will be applied.
Additional analyses may be defined before DB lock at the end of the treatment phase or during the execution of the planned analysis or after its completion.

11.1 Hypothesis

There is not statistical hypothesis for this descriptive phase I trial.

11.2 Determination of Sample Size

As the primary objective of this trial is the assessment of safety and tolerability, formal statistical calculations were not used to determine the sample size. The planned number of 3 patients for the dose escalation and 9 for the multiple dosing is considered adequate in the context of the overall trial objectives.

11.3 Statistical criteria of termination of trial

There are no statistical stopping rules for this trial. Guidance regarding the discontinuation of the trial subjects, cohorts or the entire trial based on reported AEs are provided in chapter **8.3 Dose modifications**.

11.4 Planned Analyses

11.4.1 Datasets to be analysed, analysis populations

Three main analysis populations will be employed

- **Safety Analysis Set**: all subjects who received at least part of a dose of the study medication
- **PK Analysis Sets**: subjects from the safety analysis set who provide sufficient data for the key PK evaluations and who do not violate any relevant selection criteria or have protocol violations affecting the assessment of PK.
- **Efficacy Analysis Set**: subjects from the safety analysis set who provide sufficient data for the key efficacy evaluations and who do not violate any relevant selection criteria or have protocol violations affecting efficacy assessments.

For all analyses, subjects will be grouped according to the cohort in which they were treated and/or the dosage they received. The composition of these analysis sets and the dose group or cohort will be finalized before DB lock for the primary who provide sufficient data for the key PK evaluations.

11.4.2 Primary Analysis

Selected safety data, in particular AE reports will be presented periodically by means of descriptive statistics in the Safety Analysis Set.
The primary safety analysis will be performed after completion of the assessments for the last patient. Safety will be tabulated by cohort and where applicable, dose level.
Key data include AE and SAE (incidence, nature, intensity, relationship to study drug), reasons for withdrawal from the study, laboratory data (including Anti-GM1 antibodies), ECG, concomitant medications, physical examination results and vital signs. Adverse events and SAEs will be coded and summarized by body system and preferred term based on the Medical Dictionary for Regulatory Activities (MedDRA).

11.4.3 Secondary Analyses

Efficacy analyses will be conducted in the Efficacy Analysis Set. In addition to descriptive summaries of efficacy parameters over time by dose/cohort, for patients in the multiple dose part, a statistical comparison of the change from baseline to the last visit will be performed, e.g., by means of the Wilcoxon signed-rank test for continuous variables. The evolution over time will also be analysed: the choice for the specific model (e.g., mixed-models for repeated measures or random coefficients

models) will be made at the time of analysis-based considerations such as the distribution and timing of observations or missing data patterns.
PK analyses will be descriptive and PK parameters will be derived directly from the plasma concentration-time curves or calculated using non-compartmental methods.

11.4.4 Interim analyses

No formal interim analysis is planned for this trial. Safety data will be periodically reviewed by the team, e.g., in the context of dose escalation or treatment continuation decisions.

11.4.5 Safety analysis

See section **11.4.2 Primary analysis**.

11.4.6 Deviation(s) from the original statistical plan

Deviations from the analysis principles outlined in this section will be documented and justified in the Clinical Study Report.

11.5 Handling of missing data and drop-outs

Unless otherwise indicated in the context of specific analyses (e.g., imputation rules for partial dates for determining baseline characteristics, or for levels under the lowest quantification limit of specific assays for PK evaluations) missing data will not be imputed.

12. QUALITY ASSURANCE AND CONTROL

InnoMedica runs a quality management system (QMS) which covers all relevant aspects of GCP and GMP.

12.1 Data handling and record keeping / archiving

12.1.1 Case Report Forms

For each enrolled trial participant, an eCRF will be maintained. All data will be entered in a coded way in the eCRF at the local site. eCRFs must be kept current to reflect subject status at each phase during the course of the trial and are part of the central acquisition. Study-related data of the patient will be collected in a coded manner. The names of the patients will not be disclosed. A code will be attributed to each patient registered. Coded identification for each patient will be as follow: [trial-ID]-[consecutive number]. Authorized to enter data into the eCRF are the local trial team staff according to the authorization list. Authorized persons will be identified by their usernames. The PI is responsible for proper training and instruction of the trial personnel filling data into the eCRF.

12.1.2 Specification of source documents

Source documents for each trial participant, including original trial related documents, medical treatment and medical history, will be available at the site of the PI and will be stored in the investigator site file (ISF). Original source data from CI Bern will be transported by the PI to his site in Konolfingen. It is the responsibility of the PI to guarantee data protection. The CI Bern will keep certified copies of source data at their site.

List of source documents:

- Informed consent form
- Patient screening, enrolment and identification log
- Patient records and medical history including reports from treating physician
- Documentation of the patient's medical treatment
- Drug accountability log
- Trial questionnaires and scores: MoCA, MDS-UPDRS, PDQ-39, ESS, SAS, BDI, NMSQuest
- Nurse working sheets
- Lab reports
- Any other relevant record to document AEs and SAEs

List of CRFs that are considered source documents:

- MoCA, MDS-UPDRS, PDQ-39, ESS, SAS, BDI, NMSQuest

12.1.3 Record keeping / archiving

All trial data will be archived for a minimum of 10 years after trial termination or premature termination of the clinical trial at InnoMedica at a secure location.

12.2 Data management

12.2.1 Data Management System

The eCRFs in this trial are implemented electronically using a dedicated electronic data capturing (EDC) system (secuTrial[redacted]). SecuTrial[redacted] is a HRA compliant database and fulfils all requirements according to ClinO Art. 18. The EDC system is activated for the trial only after successfully passing a formal test procedure. All data entered in the eCRFs are stored on a Linux server in a dedicated Oracle database (DB).
Responsibility for hosting the EDC system and the DB lies with Inselspital Bern.

12.2.2 Data security, access and back-up

The server hosting the EDC system and the DB is kept in a locked server-room. Only the system administrators have direct access to the server and back-up tapes. A role concept with personal passwords (site investigator, statistician, monitor, administrator etc.) regulates permission for each user to use the system and DB as he/she requires.
All data entered into the eCRFs are transferred to the DB using Transport Layer Security (TLS) encryption. Each data point has attributes attached to it identifying the user who entered it with the exact time and date. Retrospective alterations of data in the DB are recorded in an audit table. Time, table, data field, original value and altered value, and the user are recorded (audit trail).
A multi-level back-up system is implemented.

12.2.3 Analysis and archiving

At the final analysis, data files will be extracted from the DB into statistical packages to be analysed. The status of the DB at this time will be recorded in special archive tables.
The trial DB with all archive tables will be securely stored by CTU Bern. InnoMedica will also keep the TMF and final reports for at least 10 years.

12.2.4 Electronic and central data validation

Data is checked by the EDC system for completeness and plausibility. Furthermore, selected data points are cross-checked for consistency with previously entered data for that participant. In addition, central data reviews will be performed by the sponsor on a regular basis to ensure completeness of the data collected and accuracy of the primary outcome data.
Before DB lock the local PI will validate the collected data with his signature.

12.3 Monitoring

InnoMedica is following a risk-adapted monitoring strategy developed according to the concept elaborated by the ADAMON group [31] and the TransCelerate position paper[32]. The different monitoring activities as well as the frequency of the visits are described in the trial-specific monitoring plan based on this risk assessment.
All source data must be accessible for auditing and monitoring. Monitors and auditors will maintain patient confidentiality. All questions from the monitors shall be answered during the monitoring visits.

12.4 Audits and Inspections

Authorities have the right to perform inspections, and InnoMedica has the right to perform on-site auditing during working hours upon reasonable prior notice. The auditor/inspector must have access to all medical records, the investigator's trial related files and correspondence, and the informed consent documentation that is relevant for this clinical trial.
PIs will allow the persons responsible for the audit or the inspection to have access to the source data/documents and PIs will answer any questions arising. All involved parties will keep the patient data strictly confidential.

12.5 Confidentiality, Data Protection

The information contained in this protocol is copyright protected by InnoMedica. This information is given for the needs of the trial and must not be disclosed to persons outside of the trial community without prior written consent of InnoMedica.
Trial-related data of the patient will be provided in a coded way. Unencrypted data from patients will not be disclosed to persons outside of a participating site nor to third parties. A unique patient identification number will be attributed to each patient registered into the trial (for more details see section 12.1.1). Identification of patients must be guaranteed using the patient screening, enrolment and identification list. In order to avoid identification errors, patient’s identification number and the year of birth have to be provided on the eCRF. Patient confidentiality will be maintained according to applicable legislation.
Direct access to source documents will be permitted for purposes of monitoring, audits and inspections. The monitoring institution (InnoMedica) and the local authorities will have access to all information necessary for such tasks during and after the trial.
Patients are informed (orally by the PI and in written with the patient information documents), that the PI can request information concerning their medical condition from their treating general practitioner after the trial treatment.

12.6 Storage of biological material and related health data

All samples of the routine laboratory assessments and of the PK analysis will immediately be destroyed after analyses. No samples will be stored.

13. PUBLICATION AND DISSEMINATION POLICY

The trial protocol will be published and all primary and secondary outcome results will be published in a peer-reviewed journal. Final decision on publishing the results will be made by InnoMedica. Authors of the publication are persons who designed, planned, and conducted the trial.
All data belong to InnoMedica. An executive board that includes the PI as chair will decide on authorship, the order of authors, journals for publication, as well as partial results and partial aspects of the final evaluation.

14. FUNDING AND SUPPORT

14.1 Funding

The trial is fully funded by InnoMedica AG Switzerland.

14.2 Other Support

No other support is to be declared here.

15. INSURANCE

InnoMedica will indemnify patients for any damage they may suffer due to participation in this trial. For this purpose, the sponsor has taken out a special insurance for clinical trials with insurance company Chubb (Police number: CHLSCA14476).
A copy of the certificate is filed in the ISF and the TMF.

16. REFERENCES

1. Mesa-Herrera, F., Taoro-González, L., Valdés-Baizabal, C., Diaz, M. & Marín, R. Lipid and lipid raft alteration in aging and neurodegenerative diseases: A window for the development of new biomarkers. *International Journal of Molecular Sciences* vol. 20 (2019).
2. Benskey, M. J., Perez, R. G. & Manfredsson, F. P. The contribution of alpha synuclein to neuronal survival and function - Implications for Parkinson's disease. *Journal of Neurochemistry* vol. 137 331–359 (2016).
3. Tan, E. K. *et al.* Parkinson disease and the immune system — associations, mechanisms and therapeutics. *Nature Reviews Neurology* vol. 16 303–318 (2020).
4. Forsayeth, J. & Hadaczek, P. Ganglioside metabolism and Parkinson's disease. *Front. Neurosci.* **12**, 1–8 (2018).
5. Chiricozzi, E. *et al.* GM1 ganglioside is a key factor in maintaining the mammalian neuronal functions avoiding neurodegeneration. *Int. J. Mol. Sci.* **21**, 1–29 (2020).
6. Ledeen, R. W. & Wu, G. The multi-tasked life of GM1 ganglioside, a true factotum of nature. *Trends in Biochemical Sciences* vol. 40 407–418 (2015).
7. Mutoh, T., Tokuda, A., Miyadai, T., Hamaguchi, M. & Fujiki, N. Ganglioside GM1 binds to the Trk protein and regulates receptor function. *Proc. Natl. Acad. Sci. U. S. A.* **92**, 5087–5091 (1995).
8. Mutoh, T., Tokuda, A., Inokuchi, J. I. & Kuriyama, M. Glucosylceramide synthase inhibitor inhibits the action of nerve growth factor in PC12 cells. *J. Biol. Chem.* **273**, 26001–26007 (1998).
9. Martinez, Z., Zhu, M., Han, S. & Fink, A. L. GM1 specifically interacts with α-synuclein and inhibits fibrillation. *Biochemistry* **46**, 1868–1877 (2007).
10. Wang, J. *et al.* Cross-Linking of GM1 Ganglioside by Galectin-1 Mediates Regulatory T Cell Activity Involving TRPC5 Channel Activation: Possible Role in Suppressing Experimental Autoimmune Encephalomyelitis. *J. Immunol.* **182**, 4036–4045 (2009).
11. Wu, G. *et al.* Mice deficient in GM1 manifest both motor and non-motor symptoms of Parkinson's disease; successful treatment with synthetic GM1 ganglioside. *Exp. Neurol.* **329**, 113284 (2020).
12. Wu, G., Lu, Z. H., Kulkarni, N., Amin, R. & Ledeen, R. W. Mice lacking major brain gangliosides develop Parkinsonism. *Neurochem. Res.* **36**, 1706–1714 (2011).
13. Huebecker, M. *et al.* Reduced sphingolipid hydrolase activities, substrate accumulation and ganglioside decline in Parkinson's disease. *Mol. Neurodegener.* **14**, 1–21 (2019).
14. Morgan, J. & Sethi, K. D. Levodopa and the progression of Parkinson's disease. *Curr. Neurol. Neurosci. Rep.* **5**, 261–262 (2005).
15. Schneider, J. S., Sendek, S., Daskalakis, C. & Cambi, F. GM1 ganglioside in Parkinson's disease: Results of a five year open study. *J. Neurol. Sci.* **292**, 45–51 (2010).
16. Schneider, J. S. *et al.* Parkinson's disease improved function with GM1 ganglioside treatment in a randomized placebo-controlled study. *Neurology* **50**, 1630–1636 (1998).
17. Schneider, J. S. *et al.* A randomized, controlled, delayed start trial of GM1 ganglioside in treated Parkinson's disease patients. *J. Neurol. Sci.* **324**, 140–148 (2013).
18. Ghidoni, R. *et al.* Uptake, cell penetration and metabolic processing of exogenously administered GM1 ganglioside in rat brain. *Neurochem. Int.* **15**, 455–465 (1989).
19. CORNELLI, U. *et al.* Pharmacokinetics of GM1 Ganglioside Following Parenteral Administration. *J. Pharm. Pharmacol.* **42**, 708–711 (1990).
20. Svennerholm, L. *et al.* Parenteral administration of GM1 ganglioside to presenile Alzheimer patients. *Acta Neurol. Scand.* **81**, 48–53 (1990).
21. Roberts, J. W., Hoeg, J. M., Maral Mouradian, M., Linfante, I. & Chase, T. N. Iatrogenic hyperlipidaemia with GM1 ganglioside. *Lancet* **342**, 115 (1993).
22. Govoni, V., Granieri, E., Manconi, M., Capone, J. & Casetta, I. Is there a decrease in Guillain-Barré syndrome incidence after bovine ganglioside withdrawal in Italy? A population-based study in the Local Health District of Ferrara, Italy. *J. Neurol. Sci.* **216**, 99–103 (2003).
23. Govoni, V. *et al. Exogenous gangliosides and Guillain-Barré syndrome An observational study in the Local Health District of Ferrara, Italy*. *Brain* vol. 120 (1997).
24. Gallo, P. *et al.* Effect of parenteral administration of GM1 on cytokines and anti-ganglioside antibody patterns. Preliminary report in normal human individuals. *J. Neuroimmunol.* **36**, 81–86 (1992).
25. Schneider, J. S., Kean, A. & DiStefano, L. GM1 ganglioside rescues substantia nigra pars compacta neurons and increases dopamine synthesis in residual nigrostriatal dopaminergic neurons in MPTP-treated mice. *J. Neurosci. Res.* **42**, 117–123 (1995).

26. Augustinsson, L. E. *et al.* Intracerebroventricular administration of gm1 ganglioside to presenile alzheimer patients. *Dement. Geriatr. Cogn. Disord.* **8**, 26–33 (1997).
27. Svennerholm, L. *et al.* Alzheimer disease - effect of continuous intracerebroventricular treatment with GM1 ganglioside and a systematic activation programme. *Dement. Geriatr. Cogn. Disord.* **14**, 128–136 (2002).
28. Hughes, A. J., Daniel, S. E., Kilford, L. & Lees, A. J. Accuracy of clinical diagnosis of idiopathic Parkinson's disease: A clinico-pathological study of 100 cases. *J. Neurol. Neurosurg. Psychiatry* **55**, 181–184 (1992).
29. Starkstein, S. E. *et al.* Reliability, validity, and clinical correlates of apathy in Parkinson's disease. *J. Neuropsychiatry Clin. Neurosci.* **4**, 134–139 (1992).
30. Verber, D., Novak, D., Borovič, M., Dugonik, J. & Flisar, D. EQUIDopa: A responsive web application for the levodopa equivalent dose calculator. *Comput. Methods Programs Biomed.* **196**, (2020).
31. Brosteanu, O. *et al.* Risk-adapted monitoring is not inferior to extensive on-site monitoring: Results of the ADAMON cluster-randomised study. *Clin. Trials* **14**, 584–596 (2017).
32. Based, R. & Initiative, M. Risk Based Monitoring Initiative POSITION PAPER : RISK-BASED MONITORING METHODOLOGY.

17. APPENDICES

Annex 1: Lab analyses List
Annex 2: Lab values
Annex 3: Instructions for PK sampling

Annex 1: Lab analyses List

Routine labs		
Cystatine C	cystc0	Serum
Albumine/globulines	agqu	Calculation
Albumine	alb	Serum
Protéines totales	eiw	Serum
Créatinine (GFR inclus)	krea	Serum
Urée	hst	Serum
Urates	hsr	Serum
Phosphates	p	Serum
Calcium	ca	Serum
Potassium	k	Serum
Sodium	na	Serum
Ferritine	fer	Serum
Lactate deshydrogénase (LDH)	ldh	Serum
Protéine C-réactive	crp	Serum
Cholestérol LDL calculé	ldlc	Serum
Cholestérol / HDL	hdlq	Calculation
Cholestérol HDL	hdl	Serum
PA (phosphatase alcaline) totale	ap	Serum
gamma-Glutamyltransférase (gamma-	ggt	Serum
ALT (GPT)	gpt	Serum
AST (GOT)	got	Serum
Bilirubine totale	bili	Serum (protected from light)
Triglycérides	trig	Serum
Cholestérol total	chol	Serum
HbA1c (IFCC)	ghb1	Blood EDTA
HbA1c (NGSP)	ghb0	Blood EDTA
Amylase pancréatique	pamyl	Serum
MCHC (CCMH)	mchc	Blood EDTA
MCH (TCMH)	hbe	Blood EDTA
MCV (VGM)	mcv	Blood EDTA
Hématocrite	hkt	Blood EDTA
Hémoglobine	hb	Blood EDTA
Erythrocytes: RDW	rdw	Blood EDTA
Erythrocytes	ec	Blood EDTA
Basophiles	ba1	Blood EDTA
Eosinophiles	eo1	Blood EDTA
Monocytes	mo1	Blood EDTA
Lymphocytes	ly1	Blood EDTA
Neutrophiles	ne1	Blood EDTA
Leucocytes	lc	Blood EDTA

	Thrombocytes	thro	Blood EDTA
Anti-GM igG			
	Ganglioside GM1 IgG	gm1g	Serum
Apolipoprotein B			
	Apolipoprotein B	apob	Serum

Annex 2: Lab values

Referenzwerte der Analysen rout / 3549, gm1g / 52250 und apob / 7320

rout/3549 beinhaltet folgende Blöcke:	Niere / Elektrolyte / Proteine<br>Leber / Lipide / Pankreas<br>Hämatogramm V

Einzelanalysen Block: Niere / Elektrolyte / Proteine:

Analyse	Wert	Alter	Sex
Cystatin C	0.64 – 1.23 mg/L		f+m
Albumin/Globulin	1.30 – 2.50 g/g		f+m
Albumin	37 – 51 g/L		f+m
Proteine gesamt	57 – 82 g/L		f+m
Kreatinin (inkl. GFR)	27 - 88 µmol/L	0 - 1 M	f+m
	16 - 39 µmol/L	2 - 12 M	f+m
	15 - 31 µmol/L	1 - 2 Y	f+m
	23 - 37 µmol/L	3 - 4 Y	f+m
	25 - 42 µmol/L	5 - 6 Y	f+m
	30 - 48 µmol/L	7 - 8 Y	f+m
	28 - 57 µmol/L	9 - 10 Y	f+m
	37 - 63 µmol/L	11 - 12 Y	f+m
	40 - 72 µmol/L	13 - 14 Y	f+m
	40 - 66 µmol/L	>=15 Y	f
	55 - 96 µmol/L	>=15 Y	m
Harnstoff	3.2 – 8.2 mmol/L		f+m
Harnsäure	59 - 271 µmol/L	0 - 30 D	f
	65 - 319 µmol/L	31 - 365 D	f
	106 - 295 µmol/L	1 - 3 Y	f
	118 - 301 µmol/L	4 - 6 Y	f
	106 - 325 µmol/L	7 - 9 Y	f
	148 - 348 µmol/L	10 - 12 Y	f
	130 - 378 µmol/L	13 - 15 Y	f
	142 - 389 µmol/L	16 - 18 Y	f
	184 - 464 µmol/L	>= 19 Y	f
	71 - 230 µmol/L	0 - 30 D	m
	71 - 330 µmol/L	31 - 365 D	m
	124 - 330 µmol/L	1 - 3 Y	m
	106 - 325 µmol/L	4 - 6 Y	m
	106 - 319 µmol/L	7 - 9 Y	m
	130 - 342 µmol/L	10 - 12 Y	m
	183 - 413 µmol/L	13 - 15 Y	m

	124 - 448 µmol/L	16 - 18 Y	m
	220 - 547 µmol/L	>= 19 Y	m
Phosphat	1.13 - 2.10 mmol/L	0 - 14 Y	f+m
Analyse	Wert	Alter	Sex
	0.81 - 1.61 mmol/L	>= 15 Y	+
Calcium	2.18 – 2.60 mmol/L		f+m
Kalium	3.5 – 5.1 mmol/L		f+m
Natrium	136 – 145 mmol/L		f+m
Ferritin	40 - 540 µg/L	0 - 14 D	f+m
	15 - 375 µg/L	15 - 30 D	f+m
	15 - 375 µg/L	1 - 5 M	f+m
	13 - 192 µg/L	6 - 11 M	f+m
	10 - 56 µg/L	1 - 15 Y	f+m
	30 - 300 µg/L	>= 16 Y	f+m

Einzelanalysen Block: Leber / Lipide / Pankreas:

Analyse	Wert	Alter	Sex
Laktatdehydrogenase (LDH)	120 – 246 U/L		f+m
C-reaktives Protein	< 10 mg/L		f+m
Cholesterin LDL berechnet	< 3.00 mmol/L		f+m
Cholesterin / HDL (Berechnung)	< 5.00 mmol/mmol		f+m
Cholesterin HDL	> 1.00 mmol/L		f+m
AP (alkalische Phosphatase) gesamt	U/L	1 - 17 Y	f+m
	46 - 116 U/L	>= 19 Y	f+m
	65 - 272 U/L	0 - 1 D	f
	66 - 295 U/L	2 - 2 D	f
	67 - 317 U/L	3 - 3 D	f
	68 - 340 U/L	4 - 4 D	f
	70 - 387 U/L	5 - 5 D	f
	73 - 411 U/L	6 - 6 D	f
	76 - 433 U/L	7 - 7 D	f
	80 - 453 U/L	8 - 8 D	f
	86 - 472 U/L	9 - 9 D	f
	92 - 490 U/L	10 - 10 D	f
	98 - 509 U/L	11 - 30 D	f
	138 - 527 U/L	2 - 6 M	f
	131 - 486 U/L	7 - 12 M	f
	112 - 410 U/L	1 - 2 Y	f
	123 - 344 U/L	3 - 10 Y	f
	116 - 392 U/L	11 - 12 Y	f
	74 - 354 U/L	13 - 14 Y	f
	47 - 220 U/L	15 - 16 Y	f

	39 - 119 U/L	17 - 18 Y	f
	60 - 266 U/L	0 - 1 D	m
	64 - 299 U/L	2 - 2 D	m
	68 - 333 U/L	3 - 3 D	m
Analyse	Wert	Alter	Sex
	75 - 400 U/L	4 - 4 D	m
	79 - 434 U/L	5 - 5 D	m
	83 - 467 U/L	6 - 6 D	m
	87 - 498 U/L	7 - 7 D	m
	92 - 527 U/L	8 - 8 D	m
	96 - 553 U/L	9 - 9 D	m
	101 - 575 U/L	10 - 10 D	m
	140 - 621 U/L	11 - 30 D	m
	143 - 562 U/L	2 - 6 M	m
	134 - 497 U/L	7 - 12 M	m
	123 - 406 U/L	1 - 2 Y	m
	121 - 320 U/L	3 - 10 Y	m
	129 - 387 U/L	11 - 12 Y	m
	119 - 443 U/L	13 - 14 Y	m
	88 - 410 U/L	15 - 16 Y	m
	57 - 242 U/L	17 - 18 Y	m
gamma-Glutamyltransferase (gamma-GT)	< 38 U/L		f
	< 73 U/L		m
ALT (GPT)	7 – 40 U/L		f+m
AST (GOT)	13 – 40 U/L		f+m
Bilirubin gesamt	1. Tag:< 85 μmol/L	0 - 1 M	f+m
	1.-2. Tag:< 154 μmol/	0 - 1 M	f+m
	3.-5. Tag:< 205 μmol/L	0 - 1 M	f+m
	> 1. Monat:< 26 μmol/L	0 - 1 M	f+m
	< 26 μmol/L	0 - 11 M	f+m
	< 21 μmol/L	1 - 99 Y	f+m
Triglyzeride	< 1.14 mmol/L	0 - 12 Y	f+m
	< 1.71 mmol/L	13 - 19 Y	f+m
Cholesterin gesamt	< 5.00 mmol/L		f+m
HbA1c (IFCC)	< 42.0 mmol/mol		f+m
HbA1c (NGSP)	< 6.1 %		f+m
Amylase pankreasspezifisch	13 – 53 U/L		f+m

Einzelanalysen Hämatogramm V:

Analyse	Wert	Alter	Sex
Mittlere Hb-Konzentration	300 - 360 g/L	0 - 3 D	f+m
	290 - 360 g/L	4 - 7 D	f+m
	280 - 360 g/L	8 - 30 D	f+m
	290 - 360 g/L	1 - 3 M	f+m
	300 - 360 g/L	4 - 6 M	f+m
	310 - 360 g/L	7 - 12 M	f+m
	310 - 360 g/L	1 - 18 Y	f+m
	330 - 358 g/L	>= 19 Y	f+m
Hb pro Erythrozyt	31.0 - 37.0 pg	0 - 3 D	f+m
	28.0 - 40.0 pg	4 - 30 D	f+m
	26.0 - 34.0 pg	1 - 3 M	f+m
	25.0 - 30.0 pg	4 - 6 M	f+m
	23.0 - 31.0 pg	7 - 24 M	f+m
	24.0 - 30.0 pg	2 - 6 Y	f+m
	25.0 - 33.0 pg	7 - 12 Y	f+m
	25.0 - 35.0 pg	13 - 18 Y	f+m
	26.0 - 34.0 pg	>= 19 Y	f+m
Mittleres Ery-Volumen	98 - 118 fL	0 - 1 D	f+m
	95 - 121 fL	2 - 3 D	f+m
	88 - 126 fL	4 - 7 D	f+m
	86 - 124 fL	8 - 15 D	f+m
	85 - 123 fL	16 - 30 D	f+m
	77 - 115 fL	1 - 3 M	f+m
	74 - 108 fL	4 - 6 M	f+m
	70 - 86 fL	7 - 24 M	f+m
	75 - 87 fL	2 - 6 Y	f+m
	77 - 95 fL	7 - 12 Y	f+m
	80 - 100 fL	>= 19 Y	f+m
	78 - 102 fL	13 - 18 Y	f
	78 - 98 fL	13 - 18 Y	m
Hämatokrit	0.420 - 0.600 L/L	0 - 1 D	f+m
	0.450 - 0.600 L/L	2 - 3 D	f+m
	0.420 - 0.660 L/L	4 - 7 D	f+m
	0.390 - 0.630 L/L	8 - 15 D	f+m
	0.310 - 0.550 L/L	16 - 30 D	f+m
	0.280 - 0.420 L/L	1 - 3 M	f+m
	0.290 - 0.410 L/L	4 - 6 M	f+m
	0.330 - 0.390 L/L	7 - 24 M	f+m
	0.340 - 0.400 L/L	2 - 6 Y	f+m
	0.350 - 0.450 L/L	7 - 12 Y	f+m

Analyse	Wert	Alter	Sex
	0.360 - 0.460 L/L	>= 13 Y	f
	0.370 - 0.490 L/L	13 - 18 Y	m
	0.410 - 0.530 L/L	>= 19 Y	m
Hämoglobin	135 - 195 g/L	0 - 1 D	f+m
	145 - 225 g/L	2 - 3 D	f+m
	135 - 215 g/L	4 - 7 D	f+m
	125 - 205 g/L	8 - 15 D	f+m
	100 - 180 g/L	16 - 30 D	f+m
	90 - 140 g/L	1 - 3 M	f+m
	95 - 135 g/L	4 - 6 M	f+m
	105 - 135 g/L	7 - 24 M	f+m
	115 - 135 g/L	2 - 6 Y	f+m
	115 - 155 g/L	7 - 12 Y	f+m
	120 - 160 g/L	13 - 18 Y	f
	120 - 160 g/L	>= 19 Y	f
	130 - 160 g/L	13 - 18 Y	m
	135 - 175 g/L	>= 19 Y	m
Erythrozyten: RDW	11.0 – 16.0 %		f+m
Erythrozyten	4.30 - 5.10 T/L	0 - 1 D	f+m
	4.00 - 6.60 T/L	2 - 3 D	f+m
	3.90 - 6.30 T/L	4 - 7 D	f+m
	3.60 - 6.20 T/L	8 - 15 D	f+m
	3.00 - 5.40 T/L	16 - 30 D	f+m
	2.70 - 4.90 T/L	1 - 3 M	f+m
	3.10 - 4.50 T/L	4 - 6 M	f+m
	3.70 - 5.30 T/L	7 - 24 M	f+m
	3.90 - 5.30 T/L	2 - 6 Y	f+m
	4.00 - 5.30 T/L	7 - 12 Y	f+m
	4.10 - 5.10 T/L	13 - 18 Y	f
	4.00 - 5.10 T/L	>= 19 Y	f
	4.50 - 5.30 T/L	13 - 18 Y	m
	4.50 - 5.90 T/L	>= 19 Y	m
Basophile	< 0.20 G/L		f+m
Eosinophile	< 0.70 G/L		f+m
Monozyten	0.16 – 0.95 G/L		f+m
Lymphozyten	2.00 - 11.00 G/L	0 - 3 D	f+m
	2.00 - 17.00 G/L	4 - 30 D	f+m
	3.00 - 16.00 G/L	1 - 24 M	f+m
	1.50 - 8.00 G/L	2 - 12 Y	f+m
	1.50 - 4.00 G/L	>= 13 Y	f+m
Neutrophile	5.00 - 28.00 G/L	0 - 3 D	f+m

Analyse	Wert	Alter	Sex
	2.00 - 10.00 G/L	4 - 30 D	f+m
	1.00 - 9.00 G/L	1 - 24 M	f+m
	1.50 - 8.50 G/L	2 - 12 Y	f+m
	1.40 - 8.00 G/L	>= 13 Y	f+m
Leukozyten	9.0 - 38.0 G/L	0 - 3 D	f+m
	5.0 - 21.0 G/L	4 - 7 D	f+m
	5.0 - 20.0 G/L	8 - 30 D	f+m
	6.0 - 17.0 G/L	1 - 24 M	f+m
	4.5 - 13.5 G/L	2 - 16 Y	f+m
	4.5 - 11.5 G/L	>= 17 Y	f+m
Thrombozyten	150 – 450 G/L		f+m

Gangliosid GM 1 IgG:

Analyse	Wert	Alter	Sex
Gangliosid GM 1 IgG	< 10 Ak Ratio		f+m
Grenzbereich:	<15 Ak Ratio		f+m

Apolipoprotein B

Analyse	Wert	Alter	Sex
Apolipoprotein B	0.41 - 1.05 g/L	0 - 12 M	f+m
	0.44 - 1.12 g/L	1 - 3 Y	f+m
	0.58 - 1.04 g/L	4 - 5 Y	f
	0.57 - 1.13 g/L	6 - 11 Y	f
	0.53 - 1.19 g/L	12 - 19 Y	f
	0.63 - 1.32 g/L	20 - 29 Y	f
	0.59 - 1.32 g/L	30 - 39 Y	f
	0.70 - 1.36 g/L	40 - 49 Y	f
	0.75 - 1.68 g/L	50 - 59 Y	f
	0.75 - 1.73 g/L	60 - 69 Y	f
	0.79 - 1.68 g/L	>= 70 Y	f
	0.58 - 1.03 g/L	4 - 5 Y	m
	0.56 - 1.05 g/L	6 - 11 Y	m
	0.55 - 1.10 g/L	12 - 19 Y	m
	0.59 - 1.30 g/L	20 - 29 Y	m
	0.63 - 1.43 g/L	30 - 39 Y	m
	0.71 - 1.52 g/L	40 - 49 Y	m
	0.75 - 1.60 g/L	50 - 59 Y	m
	0.81 - 1.56 g/L	60 - 69 Y	m
	0.73 - 1.52 g/L	>= 70 Y	m

Annex 3: PK SAMPLES – INSTRUCTIONS FOR BLOOD COLLECTION, PROCESSING AND STORAGE

Overview of PK sampling schedule

Sample number	Scheduled time-points		
	**Week**	**Day**	**Time (min or h)**
1	1	1	0 (pre-dose)
2	1	1	5 min
3	1	1	30 min
4	1	1	1 h
5	1	1	4 h
6	1	1	8 h
7	1	2	24 h
8	1	3	48 h
9	1	4	72 h
10	1	5	96 h

PK sample preparation

WHENEVER POSSIBLE, THE BLOOD AND PLASMA SAMPLES SHOULD BE PROTECTED FROM LIGHT!

- Label the cryotube with trial name, sponsor name, corresponding sample number (see overview of PK sampling schedule), UPN and actual date and time.
- Actual date and time should as well be completed in the study documentation. These measures will be recorded on the CRF.
- Collect venous blood should into K3-EDTA tubes.
- Store tubes in an upright position on ice until centrifugation (maximum time of 30 minutes).
- The centrifugation will be done for 20 minutes at 2000 g and 5°C.
- After centrifugation, transfer the supernatant (approx. 1ml plasma) to the labelled 1ml cryotube.

PK sample storage and shipment

PK samples should always be placed for storage in an upright position in the provided cryobox at ≤ -70 °C. PK samples should be kept at the site until request for shipment by the sponsor. All PK samples will be shipped at once after samples from all patients in the part 2 of the trial were collected.

Material

All necessary material will be provided by the sponsor at the beginning of part 2 of the trial:

- 2.7 ml K3-EDTA tubes
- 1 ml cryotubes
- Cryo-box
- Labels for tubes
