# Supplementary material for: Safety and tolerability of intravenous liposomal GM1 in patients with Parkinson disease: A single-center open-label clinical phase I trial (NEON trial)
Source: PLoS Med. 2025 May 13;22(5):e1004472. doi: 10.1371/journal.pmed.1004472 (PMC12101738; doi:10.1371/journal.pmed.1004472)
Supplement: S1 Text — (PDF) [file pmed.1004472.s003.pdf]

S1 Text: Laboratory analysis list

Sodium, potassium, calcium, phosphate, creatinine, urea, albumin, total protein, cystatin C, lactate dehydrogenase, ferritin, HbA1c, ALT, AST, total bilirubin, pancreatic amylase, alkaline phosphatase, cholesterol (total, LDL, HDL), apolipoprotein B, differentiated red and white blood cell count, thrombocytes, ganglioside GM1 IgG antibodies
