# Supplementary material for: Safety and tolerability of intravenous liposomal GM1 in patients with Parkinson disease: A single-center open-label clinical phase I trial (NEON trial)
Source: PLoS Med. 2025 May 13;22(5):e1004472. doi: 10.1371/journal.pmed.1004472 (PMC12101738; doi:10.1371/journal.pmed.1004472)
Supplement: S1 Table — (PDF) [file pmed.1004472.s004.pdf]

SBQ-no	PatID	Sample no.	Day	Time [h]	GM1 [ng/mL]
s0001	PNB1k	1	1	0	58.5
s0002	PNB1k	2	1	0.083	11800
s0003	PNB1k	3	1	0.5	66100
s0004	PNB1k	4	1	1	138000
s0005	PNB1k	5	1	4	225000
s0006	PNB1k	6	1	8	200000
s0007	PNB1k	7	2	24	76800
s0008	PNB1k	8	3	48	13400
s0009	PNB1k	9	4	72	2460
s0010	PNB1k	10	5	96	1140
s0011	PNB2j	1	1	0	< 50.0
s0012	PNB2j	2	1	0.083	12400
s0013	PNB2j	3	1	0.5	41300
s0014	PNB2j	4	1	1	63200
s0015	PNB2j	5	1	4	91900
s0016	PNB2j	6	1	8	82500
s0017	PNB2j	7	2	24	26300
s0018	PNB2j	8	3	48	8450
s0019	PNB2j	9	4	72	1660
s0020	PNB2j	10	5	96	667
s0021	PNB6v	1	1	0	65.8
s0022	PNB6v	2	1	0.083	4690
s0023	PNB6v	3	1	0.5	23000
s0024	PNB6v	4	1	1	50600
s0025	PNB6v	5	1	4	83900
s0026	PNB6v	6	1	8	52500
s0027	PNB6v	7	2	24	35600
s0028	PNB6v	8	3	48	9800
s0029	PNB6v	9	4	72	4160
s0030	PNB6v	10	5	96	2640
s0031	PNB4d	1	1	0	< 50.0
s0032	PNB4d	2	1	0.083	7970
s0033	PNB4d	3	1	0.5	34200
s0034	PNB4d	4	1	1	117000
s0035	PNB4d	5	1	4	144000
s0036	PNB4d	6	1	8	124000
s0037	PNB4d	7	2	24	44700
s0038	PNB4d	8	3	48	103000
s0039	PNB4d	9	4	72	2400
s0040	PNB4d	10	5	96	478
s0041	PNB9a	1	1	0	61.0
s0042	PNB9a	2	1	0.083	19500
s0043	PNB9a	3	1	0.5	93600
s0044	PNB9a	4	1	1	199000
s0045	PNB9a	5	1	4	173000
s0046	PNB9a	6	1	8	145000
s0047	PNB9a	7	2	24	46800
s0048	PNB9a	8	3	48	5380
s0049	PNB9a	9	4	72	1120
s0050	PNB9a	10	5	96	453
s0051	PNB3z	1	1	0	65.4
s0052	PNB3z	2	1	0.083	11600
s0053	PNB3z	3	1	0.5	65100
s0054	PNB3z	4	1	1	122000
s0055	PNB3z	5	1	4	147000
s0056	PNB3z	6	1	8	145000
s0057	PNB3z	7	2	24	72300
s0058	PNB3z	8	3	48	13200
s0059	PNB3z	9	4	72	4890
s0060	PNB3z	10	5	96	1550
s0061	PNB2w	1	1	0	71.1
s0062	PNB2w	2	1	0.083	10800
s0063	PNB2w	3	1	0.5	91400
s0064	PNB2w	4	1	1	165000
s0065	PNB2w	5	1	4	582000
s0066	PNB2w	6	1	8	222000
s0067	PNB2w	7	2	24	119000
s0068	PNB2w	8	3	48	31300
s0069	PNB2w	9	4	72	9620
s0070	PNB2w	10	5	96	4090
s0071	PNB8t	1	1	0	69.2
s0072	PNB8t	2	1	0.083	26700
s0073	PNB8t	3	1	0.5	108000
s0074	PNB8t	4	1	1	216000
s0075	PNB8t	5	1	4	290000
s0076	PNB8t	6	1	8	277000
s0077	PNB8t	7	2	24	198000
s0078	PNB8t	8	3	48	120000
s0079	PNB8t	9	4	72	73700
s0080	PNB8t	10	5	96	36300
s0081	PNB5h	1	1	0	84.5
s0082	PNB5h	2	1	0.083	11500
s0083	PNB5h	3	1	0.5	51200
s0084	PNB5h	4	1	1	105000
s0085	PNB5h	5	1	4	146000
s0086	PNB5h	6	1	8	121000
s0087	PNB5h	7	2	24	31400
s0088	PNB5h	8	3	48	6790
s0089	PNB5h	9	4	72	1480
s0090	PNB5h	10	5	96	774
