# Supplementary material for: Safety and tolerability of intravenous liposomal GM1 in patients with Parkinson disease: A single-center open-label clinical phase I trial (NEON trial)
Source: PLoS Med. 2025 May 13;22(5):e1004472. doi: 10.1371/journal.pmed.1004472 (PMC12101738; doi:10.1371/journal.pmed.1004472)
Supplement: S3 Table — (PDF) [file pmed.1004472.s006.pdf]

PatID	Mild	Moderate	Severe	**total**
PNB7y	72	3	0	75
PNB9c	48	4	0	52
PNB0r	32	3	1	36
PNB1k	13	0	0	13
PNB2j	21	1	0	22
PNB6v	12	0	1	13
PNB4d	10	0	0	10
PNB9a	5	2	3	10
PNB3z	3	3	0	6
PNB2w	9	2	0	11
PNB8t	31	10	0	41
PNB5h	11	4	0	15
**total**	**267**	**32**	**5**	**304**
mean	22.25	2.67	0.42	25.33
range	3 - 72	0-10	0-3	6 - 75
