# Supplementary material for: Safety and tolerability of intravenous liposomal GM1 in patients with Parkinson disease: A single-center open-label clinical phase I trial (NEON trial)
Source: PLoS Med. 2025 May 13;22(5):e1004472. doi: 10.1371/journal.pmed.1004472 (PMC12101738; doi:10.1371/journal.pmed.1004472)
Supplement: S4 Table — (PDF) [file pmed.1004472.s007.pdf]

PatID	not related	unlikely	possibly	probably	definitely	**total**
PNB7y	2	22	49	2	0	75
PNB9c	3	18	17	6	8	52
PNB0r	4	23	9	0	0	36
PNB1k	6	3	4	0	0	13
PNB2j	2	11	4	2	3	22
PNB6v	1	7	4	1	0	13
PNB4d	0	5	3	2	0	10
PNB9a	2	2	2	0	4	10
PNB3z	5	0	0	0	1	6
PNB2w	4	6	1	0	0	11
PNB8t	9	16	6	4	6	41
PNB5h	3	10	1	0	1	15
**total**	**41**	**123**	**100**	**17**	**23**	**304**
mean	3.42	10.25	8.33	1.42	1.92	25.33
range	0-9	0-22	0-49	0-6	0-8	6 - 75
