# Supplementary material for: Safety and tolerability of intravenous liposomal GM1 in patients with Parkinson disease: A single-center open-label clinical phase I trial (NEON trial)
Source: PLoS Med. 2025 May 13;22(5):e1004472. doi: 10.1371/journal.pmed.1004472 (PMC12101738; doi:10.1371/journal.pmed.1004472)
Supplement: S5 Table — (PDF) [file pmed.1004472.s008.pdf]

Variable	Week	Median (min,max)	Median change from Baseline (min, max)	p-value (Wilcoxon)	Mean (SD)	Mean change from Baseline (SD)
Total cholesterol	Baseline (week 1 assessment)	5.81 (4.20, 6.27)	-	-	5.44 (0.71)	-
(mmol/L)	Week 2	5.64 (3.79, 6.14)	-0.13 (-0.89, 0.40)	0.398	5.32 (0.84)	-0.12 (0.37)
	Week 3	5.35 (3.83, 6.35)	-0.09 (-0.85, 0.69)	0.577	5.36 (0.89)	-0.08 (0.48)
	Week 4	5.15 (3.90, 6.62)	-0.34 (-1.44, 0.67)	0.147	5.13 (0.86)	-0.31 (0.59)
	Week 5	5.25 (3.80, 6.46)	-0.13 (-0.88, 0.54)	0.24	5.27 (0.90)	-0.18 (0.44)
	Week 6	5.56 (3.80, 6.18)	-0.25 (-0.66, 0.36)	0.056	5.23 (0.83)	-0.21 (0.32)
	Week 7	5.61 (3.85, 6.80)	-0.15 (-0.83, 0.88)	0.533	5.40 (0.92)	-0.05 (0.43)
	Week 8	5.78 (3.96, 6.58)	0.03 (-0.50, 0.61)	0.638	5.50 (0.89)	0.05 (0.34)
	Final Assessment	5.48 (4.05, 6.56)	0.10 (-0.41, 0.64)	0.7	5.50 (0.85)	0.05 (0.40)
	Follow up	5.12 (4.01, 6.49)	-0.46 (-1.84, 0.92)	0.278	5.15 (0.78)	-0.30 (0.75)
LDL cholesterol	Baseline (week 1 assessment)	3.44 (2.46, 4.22)	-	-	3.35 (0.64)	-
(mmol/L)	Week 2	3.27 (2.00, 4.62)	-0.12 (-0.84, 0.69)	0.359	3.21 (0.87)	-0.14 (0.46)
	Week 3	3.20 (2.28, 4.74)	-0.17 (-0.62, 0.88)	0.898	3.40 (0.83)	0.05 (0.54)
	Week 4	3.10 (1.73, 4.54)	-0.35 (-0.95, 0.61)	0.147	3.09 (0.79)	-0.26 (0.52)
	Week 5	3.07 (1.90, 4.80)	-0.11 (-1.00, 0.87)	0.24	3.24 (0.90)	-0.11 (0.46)
	Week 6	3.28 (2.04, 4.12)	-0.11 (-0.50, 0.47)	0.413	3.26 (0.66)	-0.09 (0.31)
	Week 7	3.72 (1.95, 4.33)	0.01 (-0.95, 0.83)	0.898	3.36 (0.83)	0.01 (0.48)
	Week 8	3.50 (1.73, 4.41)	0.05 (-1.12, 0.61)	0.898	3.27 (0.89)	-0.08 (0.56)
	Final Assessment	3.72 (1.94, 4.57)	0.25 (-0.55, 0.89)	0.24	3.55 (0.75)	0.20 (0.51)
	Follow up	2.90 (2.48, 4.52)	-0.20 (-1.25, 0.69)	0.824	3.25 (0.70)	-0.10 (0.55)
HDL cholesterol	Baseline (week 1 assessment)	1.22 (0.90, 2.76)	-	-	1.35 (0.50)	-
(mmol/L)	Week 2	1.08 (0.72, 2.47)	-0.14 (-0.29, -0.05)	0.004	1.20 (0.45)	-0.15 (0.07)
	Week 3	1.13 (0.79, 2.59)	-0.16 (-0.27, -0.02)	<0.001	1.19 (0.49)	-0.16 (0.07)
	Week 4	1.05 (0.72, 2.23)	-0.18 (-0.53, -0.10)	<0.001	1.14 (0.40)	-0.21 (0.12)
	Week 5	1.09 (0.70, 2.24)	-0.18 (-0.52, -0.05)	<0.001	1.15 (0.40)	-0.20 (0.13)
	Week 6	1.04 (0.72, 2.20)	-0.18 (-0.56, -0.10)	0.004	1.11 (0.39)	-0.24 (0.15)
	Week 7	0.97 (0.72, 2.53)	-0.23 (-0.39, 0.00)	0.006	1.13 (0.50)	-0.22 (0.11)
	Week 8	1.13 (0.72, 2.52)	-0.18 (-0.36, 0.05)	0.005	1.18 (0.48)	-0.17 (0.10)
	Final Assessment	1.14 (0.76, 2.41)	-0.14 (-0.35, 0.09)	0.021	1.21 (0.44)	-0.13 (0.14)
	Follow up	1.14 (0.77, 2.45)	-0.11 (-0.34, 0.05)	0.008	1.22 (0.44)	-0.13 (0.12)
Triglycerides	Baseline (week 1 assessment)	1.54 (0.59, 2.71)	-	-	1.64 (0.71)	-
(mmol/L)	Week 2	1.44 (0.82, 3.60)	0.37 (-0.81, 1.56)	0.123	2.00 (1.13)	0.35 (0.69)
	Week 3	1.77 (0.72, 2.90)	0.09 (-0.94, 1.10)	0.563	1.71 (0.75)	0.06 (0.58)
	Week 4	1.35 (0.64, 4.52)	0.05 (-0.60, 2.98)	0.625	1.99 (1.22)	0.35 (1.04)
	Week 5	1.89 (0.70, 3.03)	0.23 (-0.76, 1.29)	0.175	1.92 (0.79)	0.28 (0.62)
	Week 6	1.72 (0.82, 3.26)	0.08 (-0.17, 1.59)	0.13	1.92 (0.81)	0.28 (0.50)
	Week 7	2.05 (1.15, 4.85)	0.44 (-0.46, 2.14)	0.042	2.24 (1.11)	0.59 (0.80)
	Week 8	2.13 (0.78, 4.47)	0.31 (-0.62, 2.99)	0.102	2.29 (1.32)	0.65 (1.08)
	Final Assessment	1.45 (0.54, 2.98)	-0.18 (-1.38, 0.67)	0.765	1.61 (0.71)	-0.03 (0.59)
	Follow up	1.12 (0.61, 2.95)	-0.20 (-1.62, 1.21)	0.32	1.49 (0.79)	-0.15 (0.69)
Apolipoprotein B	Baseline (week 1 assessment)	0.91 (0.71, 1.11)	-	-	0.94 (0.13)	0.00 (0.00)
(g/L)	Week 2	0.83 (0.66, 1.13)	0.02 (-0.25, 0.08)	>0.999	0.92 (0.17)	-0.02 (0.10)
	Week 3	0.87 (0.70, 1.16)	0.02 (-0.21, 0.11)	0.638	0.94 (0.16)	0.01 (0.09)
	Week 4	0.88 (0.74, 1.20)	0.02 (-0.17, 0.09)	>0.999	0.93 (0.16)	-0.01 (0.09)
	Week 5	0.94 (0.72, 1.24)	0.02 (-0.19, 0.22)	0.126	0.97 (0.18)	0.03 (0.10)
	Week 6	0.96 (0.74, 1.18)	0.06 (-0.17, 0.15)	0.24	0.98 (0.16)	0.04 (0.10)
	Week 7	1.01 (0.74, 1.22)	0.08 (-0.17, 0.30)	0.036	1.02 (0.16)	0.08 (0.12)
	Week 8	1.08 (0.80, 1.32)	0.10 (-0.11, 0.27)	0.032	1.04 (0.17)	0.11 (0.13)
	Final Assessment	1.12 (0.88, 1.38)	0.14 (-0.03, 0.27)	0.008	1.07 (0.16)	0.14 (0.11)
	Follow up	0.96 (0.79, 1.25)	0.04 (-0.13, 0.19)	0.168	0.98 (0.14)	0.05 (0.10)
Total/HDL cholesterol	Baseline (week 1 assessment)	4.42 (2.14, 5.15)	-	-	4.29 (0.84)	-
(mmol/mmol)	Week 2	4.85 (2.49, 5.85)	0.50 (-0.57, 1.14)	0.024	4.72 (1.06)	0.43 (0.46)
	Week 3	5.28 (2.38, 5.96)	0.55 (-0.18, 1.54)	0.002	4.83 (1.02)	0.53 (0.41)
	Week 4	4.87 (2.59, 6.09)	0.39 (-0.20, 1.67)	0.01	4.78 (1.06)	0.48 (0.55)
	Week 5	5.05 (2.53, 6.15)	0.57 (-0.45, 1.43)	0.01	4.85 (1.06)	0.55 (0.53)
	Week 6	5.24 (2.64, 6.39)	0.49 (-0.11, 2.04)	0.002	4.99 (1.06)	0.70 (0.58)
	Week 7	5.35 (2.69, 6.73)	0.79 (0.21, 2.11)	<0.001	5.15 (1.05)	0.86 (0.49)
	Week 8	5.25 (2.54, 6.08)	0.54 (-0.01, 1.86)	0.002	4.99 (1.01)	0.70 (0.55)
	Final Assessment	4.99 (2.72, 6.13)	0.55 (-0.47, 1.51)	0.01	4.80 (0.96)	0.51 (0.55)
	Follow up	4.57 (2.23, 5.82)	0.08 (-0.36, 1.40)	0.365	4.52 (1.08)	0.22 (0.58)
