# Supplementary material for: Safety and tolerability of intravenous liposomal GM1 in patients with Parkinson disease: A single-center open-label clinical phase I trial (NEON trial)
Source: PLoS Med. 2025 May 13;22(5):e1004472. doi: 10.1371/journal.pmed.1004472 (PMC12101738; doi:10.1371/journal.pmed.1004472)
Supplement: S6 Table — (PDF) [file pmed.1004472.s009.pdf]

PatID	visit_name	UPDRS I score	UPDRS II score best	UPDRS II score worst	UPDRS IV score	UPDRS III score off	UPDRS III score on	UPDRS total score best off	LEDD total	MoCA score	PDQ39-SI	PDQ39 activities	PDQ39 bodily	PDQ39 cognitive	PDQ39 communication	PDQ39 mobility	PDQ39 social support	PDQ39 stigma	PDQ39 emotional	ESS	BDI	NMSQ	SAS
PNB7y	Baseline	9	10	10	0	17	3	36	1010	28	28.49	8.33	58.33	37.5	25	17.5	8.33	43.75	29.17	19	13	9	3
PNB7y	Final Assessment	7	3	3	0	8	5	18	1010	29	22.19	0	41.67	50	16.67	2.5	8.33	50	8.33	16	15	8	4
PNB9c	Baseline	6	2	2	0	8	4	16	375	28	14.9	0	16.67	0	0	2.5	16.67	37.5	45.83	5	15	5	14
PNB9c	Final Assessment	1	1	1	0	10	9	12	375	26	10.68	0	16.67	12.5	0	0	8.33	31.25	16.67	8	11	1	10
PNB0r	Baseline	11	4	4	0	10	4	25	550	30	15.26	29.17	41.67	18.75	0	7.5	0	12.5	12.5	3	10	15	4
PNB0r	Final Assessment	9	3	3	0	1	1	13	550	30	12.87	12.5	41.67	6.25	16.67	5	0	12.5	8.33	3	10	6	10
PNB1k	Baseline	4	0	0	0	6	5	10	450	29	16.25	0	41.67	0	25	5	16.67	12.5	29.17	6	5	2	15
PNB1k	Final Assessment	2	0	0	0	3	3	5	525	25	13.59	0	16.67	12.5	8.33	2.5	16.67	18.75	33.33	8	7	1	14
PNB2j	Baseline	5	7	7	0	17	4	29	750	26	15.26	29.17	33.33	12.5	0	20	8.33	6.25	12.5	3	12	7	15
PNB2j	Final Assessment	1	4	4	0	8	2	13	750	25	17.03	25	33.33	18.75	16.67	17.5	16.67	0	8.33	4	5	1	16
PNB6v	Baseline	14	17	17	3	17	10	51	1275	29	33.91	33.33	50	37.5	50	15	33.33	31.25	20.83	9	4	7	12
PNB6v	Final Assessment	11	18	18	3	19	7	51	1300	28	28.65	20.83	50	18.75	75	12.5	8.33	31.25	12.5	9	8	7	16
PNB4d	Baseline	17	23	25	12	36	10	90	1095	29	30.47	29.17	41.67	56.25	66.67	12.5	25	0	12.5	14	9	15	12
PNB4d	Final Assessment	13	21	22	11	12	9	58	1095	30	27.55	12.5	58.33	18.75	75	22.5	16.67	0	16.67	10	6	10	10
PNB9a	*Baseline*	*14*	*5*	*5*	*3*	*15*	*9*	*37*	*395*	*27*	*20.89*	*8.33*	*25*	*43.75*	*33.33*	*2.5*	*0*	*12.5*	*41.67*	*14*	*16*	*11*	*12*
PNB3z	Baseline	2	3	3	1	22	9	28	750	26	11.09	25	0	31.25	0	7.5	0	12.5	12.5	4	10	6	19
PNB3z	Final Assessment	2	3	3	1	18	10	24	750	29	9.48	4.17	8.33	31.25	0	5	0	6.25	20.83	12	3	6	18
PNB2w	Baseline	3	4	4	3	9	2	19	340	29	19.17	0	16.67	0	0	20	8.33	50	58.33	3	19	8	15
PNB2w	Final Assessment	7	2	2	0	6	1	15	340	29	15.52	0	0	0	0	20	0	62.5	41.67	2	21	1	16
PNB8t	Baseline	11	11	11	2	17	8	41	500	29	9.69	8.33	33.33	25	8.33	2.5	0	0	0	8	8	11	8
PNB8t	Final Assessment	7	2	2	0	7	2	16	500	27	9.17	4.17	25	25	8.33	2.5	0	0	8.33	1	5	5	10
PNB5h	Baseline	4	7	7	3	18	12	32	1110	27	26.77	12.5	50	31.25	25	22.5	16.67	18.75	37.5	11	11	10	11
PNB5h	Final Assessment	5	0	13	1	11	17	30	1100	24	22.55	16.67	41.67	18.75	25	7.5	8.33	25	37.5	13	11	2	8
